# Molecular-level observation of the self-assembly of a virus-like particle

**DOI:** 10.1038/s41586-026-10948-z

**Published:** 2026-09-16

**Authors:** Roi Asor, Dan Loewenthal, Diana Melnyk, Tiong Kit Tan, Philipp Kukura

**Affiliations:** 1 Physical and Theoretical Chemistry Laboratory, Department of Chemistry, University of Oxford, Oxford, UK; 2 Kavli Institute for Nanoscience Discovery, University of Oxford, Oxford, UK; 3 MRC Translational Immune Discovery Unit, Weatherall Institute of Molecular Medicine, University of Oxford, Oxford, UK; 4 Chinese Academy of Medical Sciences Oxford Institute, Nuffield Department of Medicine, University of Oxford, Oxford, UK

**Keywords:** Single-molecule biophysics, Biological physics, Supramolecular assembly, Thermodynamics, Kinetics

## Abstract

Biomolecular assembly is a cornerstone of cellular organization. Revealing its underlying principles is essential for understanding biological function^1,2^ and malfunction in disease^3,4^. Viral capsid assembly is the archetypal self-assembly system^5–7^, which has been central in establishing the fundamental principles underpinning biomolecular assembly and the development of new biomaterials^8–10^ and therapeutics^11,12^. Yet, despite decades of experimental efforts, observation and quantification of virus self-assembly pathways and dynamics have remained elusive^13^. Here we combine mass photometry (MP)^14^ with a single-molecule trapping method to monitor the real-time assembly of individual virus-like particles (VLPs) with molecular resolution. We show that weak and reversible multivalent interactions control the assembly process by facilitating stochastic selection of a limited set of on-path, topologically closed intermediate structures. Assembly is finely tuned by the transition rates between these intermediates, proceeding through a sequence of effectively irreversible first-passage events. The corresponding first-passage times arise from the VLP symmetry, creating temporal separation between the formation of the first topologically closed intermediate and subsequent elongation. This results in a nucleation-and-growth mechanism that yields an equilibrium distribution consistent with the law of mass action, despite the overall irreversibility of assembly. Characterization of the thermodynamics and kinetics of the process reveals how the system specifically assembles into one final structure with high fidelity despite thousands of available assembly intermediates. More broadly, our approach provides a general framework for visualizing and quantifying the dynamics of multimeric biological machines at the molecular level.

## Abstract (web summary)

Using mass photometry combined with a single-molecule trapping method allows real-time monitoring of the assembly of individual virus-like particles with molecular resolution, enabling observation and quantification of their self-assembly pathways and dynamics.

## Main

Protein self-assembly is crucial for biological function, enabling emergent properties such as structural stability, cooperativity and dynamic response that proteins cannot achieve individually. Driven by a set of non-covalent homo- and hetero-interaction principles^2,15^, self-assembly spans several scales and dimensions^16^, from soluble oligomers to surface-associated clusters on membranes. In solution, it underpins the dynamic organization of the cytoskeleton, the formation of bacterial microcompartments, viral capsids and membraneless organelles^5,17–19^, whereas on membranes, it mediates signalling, immune recognition and virus–host interactions^20–24^. Studying and quantifying assembly across spatial and temporal scales is thus essential for explaining how structure, symmetry and dynamics are coupled to enable biological function, how they are dysregulated in disease^3,4^ and how they may be used for therapeutic intervention.

An archetypal example for biomolecular self-assembly processes are simple spherical viruses, in which identical protein subunits interact to form multivalent building blocks that further assemble into highly regular symmetric, closed capsid structures, encapsulating the viral genetic material^6,7,25,26^. This process showcases self-assembly dynamics finely tuned to distinct phases of the viral life cycle, yet capable of proceeding spontaneously with high yield, enabling its recapitulation in relatively simple in-vitro experimental systems across several viruses^13^. Acquiring pathway-level understanding of the underlying molecular dynamics is critical for the rational design of new artificial capsid-like protein nanocages^9,10^, as well as the design and development of antiviral treatments^27,28^.

The fundamental challenge of visualizing the molecular details of simple spherical capsid assembly comes down to two factors. First, the self-limiting free energy landscape results in a well-defined, finite and closed complex and involves weak and reversible pairwise interactions. These properties make assembly stochastic, whereas key intermediates are typically short-lived and of low abundance^29–33^. Second, the involvement of tens to thousands of subunits results in an exponentially increasing number of molecular assembly paths and a high degree of heterogeneity in solution^34,35^. As a result, ensemble-based approaches have struggled with this heterogeneity, relying on complex analysis or modelling^29,34,36–39^.

Single-molecule approaches have been applied to overcome such heterogeneity: charge detection mass spectrometry and resistive pulse sensing provide ensemble-averaged snapshot observations of a subset of stable intermediates but struggle with the temporal resolution required to capture dynamics, with charge detection mass spectrometry additionally not operating in solution^40–42^. Single-molecule fluorescence yields dynamics but struggles in quantifying the degree of oligomerization^43,44^, whereas atomic force spectroscopy reveals structure and interactions at the protein subunit scale only in 2D, in which full capsids cannot assemble^45,46^. In terms of monitoring capsid assembly, interferometric scattering microscopy has been used to monitor the formation of capsids on an RNA template^47^, yielding information on nucleation and elongation at the single-particle level. This study, however, did not have the temporal resolution and sensitivity required to reveal molecular-level details of the assembly process. As a result, despite decades of dedicated study, no experimental approach has been successful in visualizing the molecular mechanism underlying capsid assembly^13^. Thus, although theoretical and computational models for assembly are established^48–52^, fundamental questions remain, such as how assembly navigates  a highly heterogeneous configurational space, what molecular mechanisms lead to or avoid kinetic traps and what sequence of molecular events result in capsid polymorphism.

Overcoming these limitations requires a new experimental framework. Such a framework must be able to: (1) quantify the thermodynamic and kinetic parameters that govern the fundamental, pairwise interactions between multivalent subunits; (2) monitor particle assembly dynamics at the ensemble level; (3) identify transient intermediates and map their relative abundances as a function of time; (4) monitor the assembly progression of single complexes by capturing the sequence of microscopic states and the transition rates between them. Here we establish such a framework by combining MP^14^ with a single-molecule trapping approach^53^, enabling complete, molecular-level characterization of the assembly process of a VLP.

## Assembly is gated by weak pairwise interactions

**Fig. 1:**
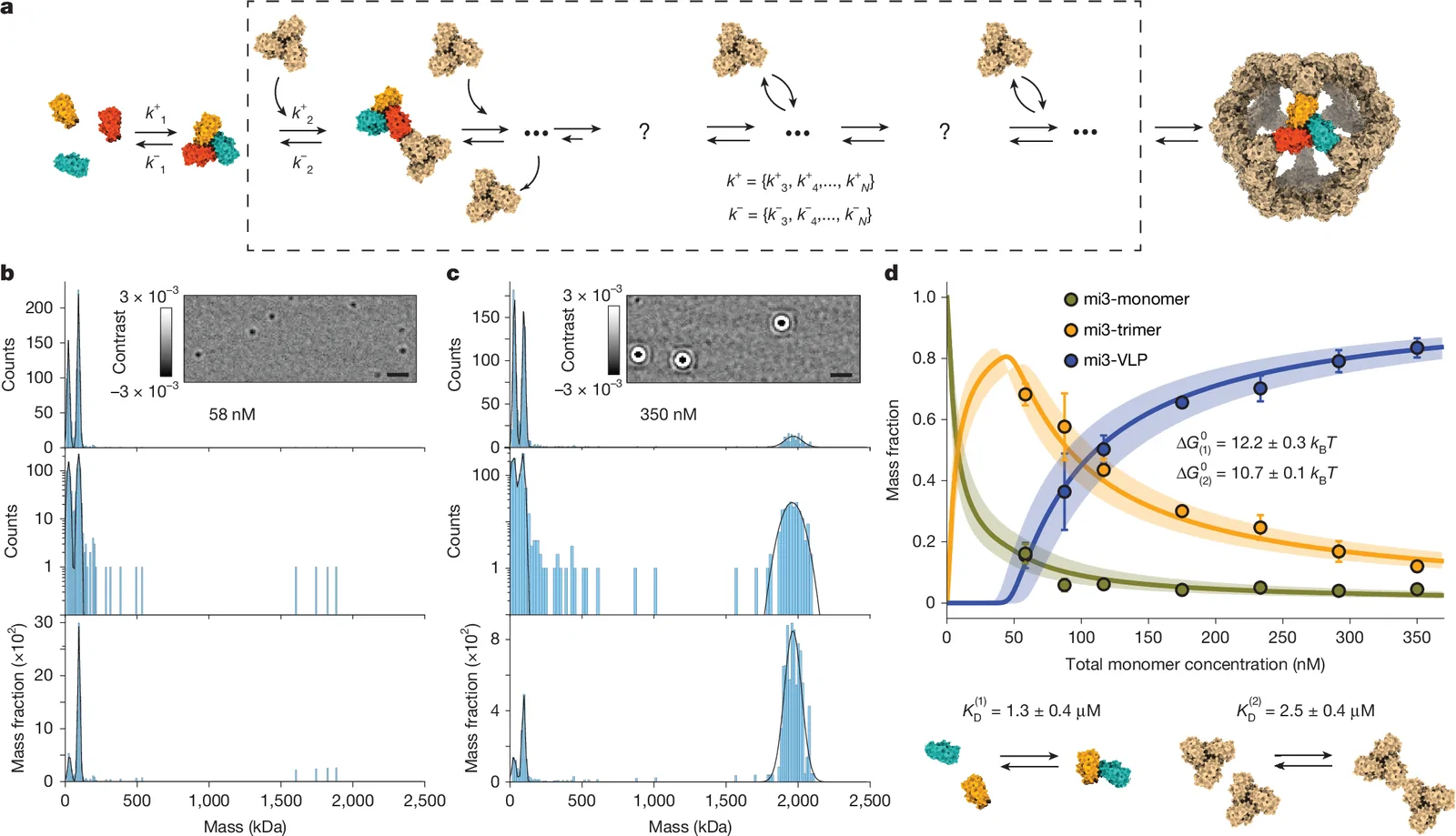
Equilibrium characterization of the self-assembly process. **a**, Illustration of the self-assembly process for the mi3-based dodecahedral VLP. **b**,**c**, Equilibrium solution mass distributions at two different mi3-monomer concentrations. Insets show representative MP images. Scale bars, 1 µm. Mass histograms are shown on linear (top), semilogarithmic (middle) and mass fraction (bottom) scales. Black lines correspond to the sum of three fitted Gaussian functions for the mi3-monomer, mi3-trimer and VLP. **d**, Experimental mass fractions for the mi3-monomer (green), mi3-trimer (orange) and assembled mi3-VLP (blue) as a function of the total protein concentration. Symbols and error bars correspond to the average and standard deviation, respectively, of three technical repeats per concentration. Solid lines correspond to a global fit (Supplementary Note 2) varying the standard free energy change for pairwise interactions between monomers ($\Delta {G}_{(1)}^{0}$) and trimers ($\Delta {G}_{(2)}^{0}$), and shaded colours and fitting errors correspond to 95% confidence interval of the fit. The expected linear relation between the total mi3-monomer concentration and the total detected mi3-monomers (Supplementary Fig. 4) is maintained, suggesting similar detection probability for all oligomeric species.

To enable an analytical understanding of the stoichiometries and topologies involved in the assembly pathway of empty VLPs, we selected an engineered, self-assembling VLP (mi3-VLP (ref. ^54^); Methods) under evaluation at present as a multivalent vaccine platform^12,55^. The mi3-VLP consists of 60 identical mi3-monomers, hierarchically assembled into a dodecahedron from 20 homotrimeric building blocks (mi3-trimers), yielding a particle with 12 pentagonal faces, sharing molecular similarity with *T* = 1 icosahedral native viral capsids (Fig. 1a). Although the geometry is dodecahedral rather than the triangulated icosahedral lattice found in viruses, we expect that the fundamental biophysical principles governing the assembly of empty capsids are conserved. The exact capsid and assembly mechanism, symmetry and local conformations, however, will govern the specific pattern of locally stable intermediates (Fig. 1a), the degeneracy values of microstates and the exact dynamics of how assembly pathways branch^31,38^.

We begin by characterizing the assembly process in solution using standard MP, in which individual proteins and complexes bind non-specifically from solution to a glass surface. At 58 nM total mi3-monomer concentration, the solution distribution is dominated by mi3-monomers and mi3-trimers at 38 ± 2 kDa (see also Supplementary Fig. 1) and 108 ± 1 kDa, respectively (Fig. 1b, top and Supplementary Figs. 2 and 3). Plotting the mass distribution on a logarithmic scale (Fig. 1b, middle) shows the extremely low abundance of intermediates in the 150–500-kDa mass range, as well as particles near the measured mass of complete mi3-VLPs (2,030 ± 10 kDa; Supplementary Note 1 and Supplementary Fig. 2b). Conversion of particle abundances into mass fractions (Fig. 1b, bottom, in which each species was weighted according to its oligomeric state) shows that mi3-monomers are predominantly found in trimeric form (65 ± 4%), followed by fully assembled VLPs (15 ± 4%), monomers (11 ± 3%) and small oligomers (9 ± 3%) (Fig. 1b, bottom). At a higher total monomer concentration (350 nM), most of the monomers are now found in assembled VLPs (82 ± 5%, at 1,982 ± 25 kDa) (Fig. 1c). The small deviation of the average mass of assembled VLPs (1,982 versus 2,030 kDa) could be caused by a fraction of particles missing one trimeric subunit owing to slow kinetics associated with capsid closure^56^. By globally fitting the law of mass action across several total protein concentrations, we can convert these relative abundances of the three dominant molecular states (mi3-monomer, mi3-trimer and VLP) into the affinities governing the assembly process^34,50^ (Fig. 1d and Supplementary Note 2), namely, ${K}_{{\rm{D}}}^{(1)}=1.3\pm 0.4\,{\rm{\mu }}{\rm{M}}$ and ${K}_{{\rm{D}}}^{(2)}=2.5\pm 0.4\,{\rm{\mu }}{\rm{M}}$ (Fig. 1d, bottom). The key pairwise interactions are thus weak relative to the pseudocritical concentrations needed for assembly (about 50 nM), which emphasizes the importance of multivalency in stabilizing these interactions.

## Reduced dimensionality drives closed-face assembly

**Fig. 2:**
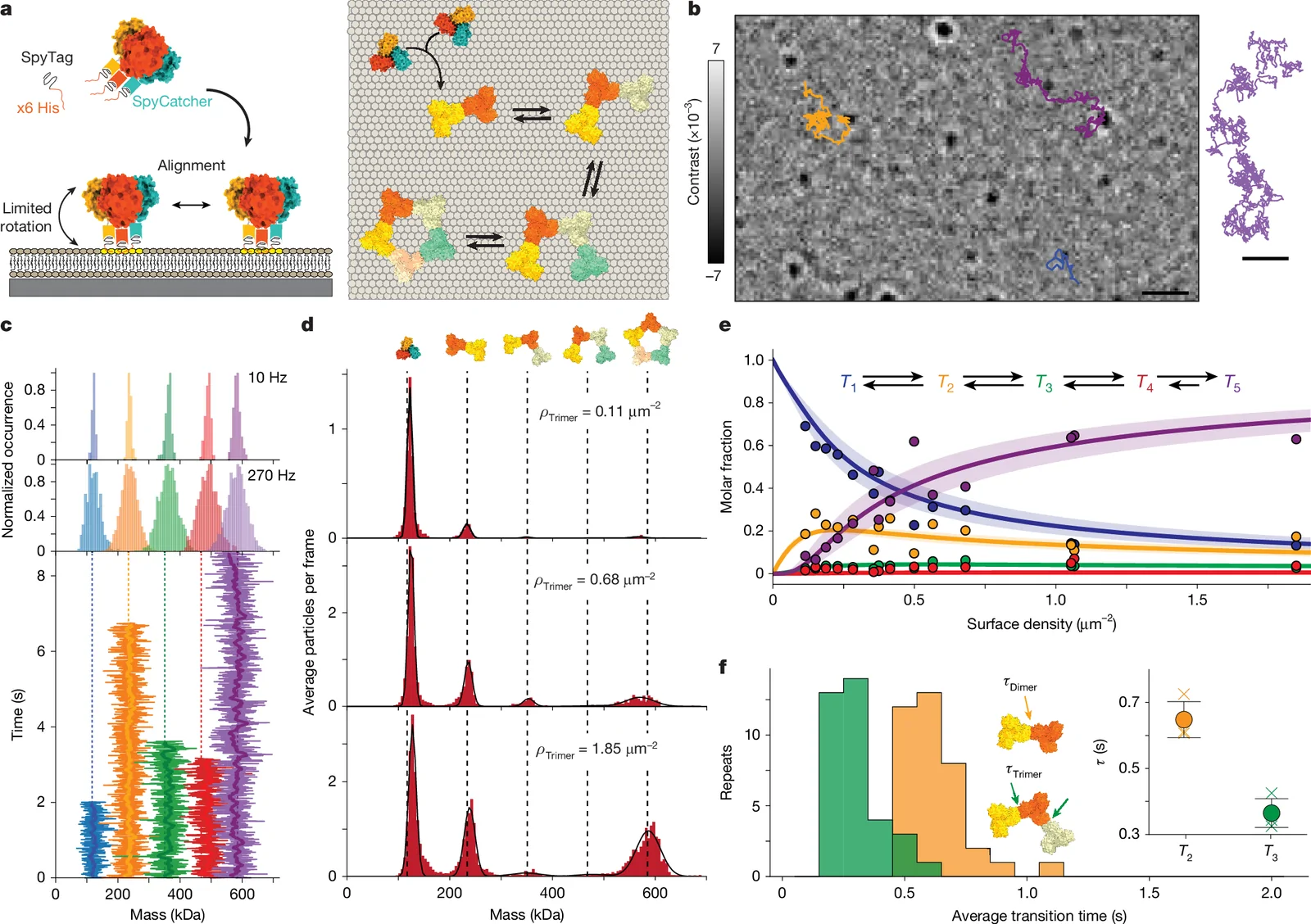
Dimensionality reduction induces the assembly of pentamers of trimers on an SLB. **a**, Tethering of mi3-subunits to an SLB using 6xHis-tag Ni-NTA lipid linking. **b**, Individual proteins and their complexes are imaged, tracked and mass monitored as they diffuse and self-assemble on the SLB. Left, representative MP image acquired at 270 Hz (Methods). Representative partial trajectories are shown for a trimer (blue), dimer of trimers (orange) and pentamer of trimers (purple). Right, an 8-s trace of a diffusing pentamer. Scale bars, 1 µm. **c**, Representative mass traces of different oligomeric states, measured at the raw frame rate (270 Hz) and after 28-frame averaging (about 10 Hz), showing the achievable mass resolution at roughly 100 ms integration time (Supplementary Fig. 7). **d**, Surface mass distributions as a function of trimer surface density (Methods). Vertical lines and illustrations show the expected masses of the different oligomers of trimers. Each histogram contains cumulative data from between 3 (for high density) and 15 (for lower density) MP measurements. Black lines correspond to the sum of the best fitted Gaussian functions used for fitting each mass peak. **e**, Mole fractions as a function of trimer surface density (symbols) and corresponding thermodynamic model (lines) (Supplementary Note 3.1 and Supplementary Fig. 6). **f**, Representative average transition time histograms for dimer (orange) and trimer (green) disassembly at a surface density of 0.72 μm^−2^. Inset, circles indicate the averaged values for the dimer (orange) and the trimer (green) over different surface densities, crosses indicate the average values at three different surface densities (averaged values for each surface density based on analysing 30 1-min-long MP movies) and the error bars represent standard deviations (see Extended Data Fig. 1c).

A notable challenge for characterizing self-limiting assembly in solution is the low abundance of assembly intermediates. In our case, intermediate states are those originating from assembly of mi3-trimers into larger oligomers, on-path to the full VLP. One approach to make higher-order assembly more favourable is to reduce the entropic penalty associated with complex formation. Here we achieve this by tethering hexahistidine-tagged (6xHis-tag) trimeric subunits to supported lipid bilayers (SLBs), where they are able to freely diffuse in two dimensions (Methods). In this 2D configuration, rotational degrees of freedom are constrained, pre-aligning trimers for oligomerization, thus increasing the interaction free energy gain per interaction^57,58^. Furthermore, surface confinement results in an increased effective concentration and therefore promotes assembly (Fig. 2a). We validated that mi3-subunits do not interact with the SLB in the absence of a tag (Supplementary Fig. 5) and therefore we expect that tethering to the 2D surface maintains the solution structure and conformation.

In contrast to the experiments shown in Fig. 1, in which we characterize solution distributions from non-specific binding of proteins to a glass surface, the data now consist of individual proteins diffusing in 2D on an SLB^21,59,60^ (Fig. 2b). Different oligomers of mi3-trimers appear at their respective mass multiples (Fig. 2c) and are resolvable by mass even at the raw frame rate of our measurement (270 Hz). In analogy to a solution-based experiment, in which increasing the solution concentration leads to a higher fraction of assembled capsids (Fig. 1c), raising the surface density of mi3-trimers increases the abundance of higher-order oligomers (Fig. 2d). We observe the expected exponential decay in higher oligomer abundance with the exception of the pentamer of mi3-trimers, which is stabilized by the further interaction associated with ring formation, as observed also for *T* = 1 icosahedral capsids^61^. Larger assemblies are not formed, as the concentration of mi3-subunits in solution is well below the pseudocritical concentration for assembly (<5 nM), whereas surface tethering prevents out-of-plane interactions. In this way, we arrest the assembly and characterize the formation of the smallest stable intermediate, a pentagonal face of mi3-trimers.

Resolving the oligomeric distribution while directly measuring the mi3-trimer surface density allows us to construct a complete thermodynamic model for the 2D assembly. Here we require only one parameter: the pairwise, standard 2D association free energy change, $\Delta {G}^{0}(2{\rm{D}})$ (Supplementary Note 3.1 and Supplementary Fig. 6). Fitting the oligomeric occupancies as a function of surface density (Fig. 2e) yields a 2D interaction free energy gain of 6.75 ± 0.2 *k*_B_*T* per contact that corresponds to ${K}_{{\rm{D}}}^{2{\rm{D}}}=0.74\pm 0.15\,{{\rm{\mu }}{\rm{m}}}^{-2}$ for mi3-trimeric subunits.

We can now use the ratio of the 2D and 3D dissociation constants, which defines a characteristic confinement length $h=\frac{{K}_{{\rm{D}}}(2{\rm{D}})}{{K}_{{\rm{D}}}(3{\rm{D}})}$, to reveal the origin of affinity enhancement on surface confinement (Supplementary Note 3.2). In our case, *h* = 0.5 ± 0.1 nm, falling within the estimated values for enhancement arising from restricted translational and rotational degrees of freedom in two dimensions^58^. By contrast, a substantial membrane-induced enthalpic stabilization would result in values that are orders of magnitude smaller. Therefore, we conclude that, thermodynamically, our surface-based measurement is representative of measurements in solution, the only exception being a different scaling originating from the reduction in dimensionality.

Thermodynamic analysis (Figs. 1d and 2e) suggests that the formation of this pentagonal face on the SLB, and the formation of the complete VLP in solution, are consistent with a single trimer–trimer contact free energy. Accordingly, we expect that any transiently stabilized intermediates or variability in transition times between states are unlikely to arise from cooperativity or alternative conformations, which would result in different fundamental interaction parameters. Instead, any assembly heterogeneity is topology driven: as assembly proceeds, the local coordination (number of intertrimer contacts per subunit) increases and ring closure adds further stabilization, thereby governing intermediate lifetimes and transition kinetics.

**Extended Data Fig. 1:**
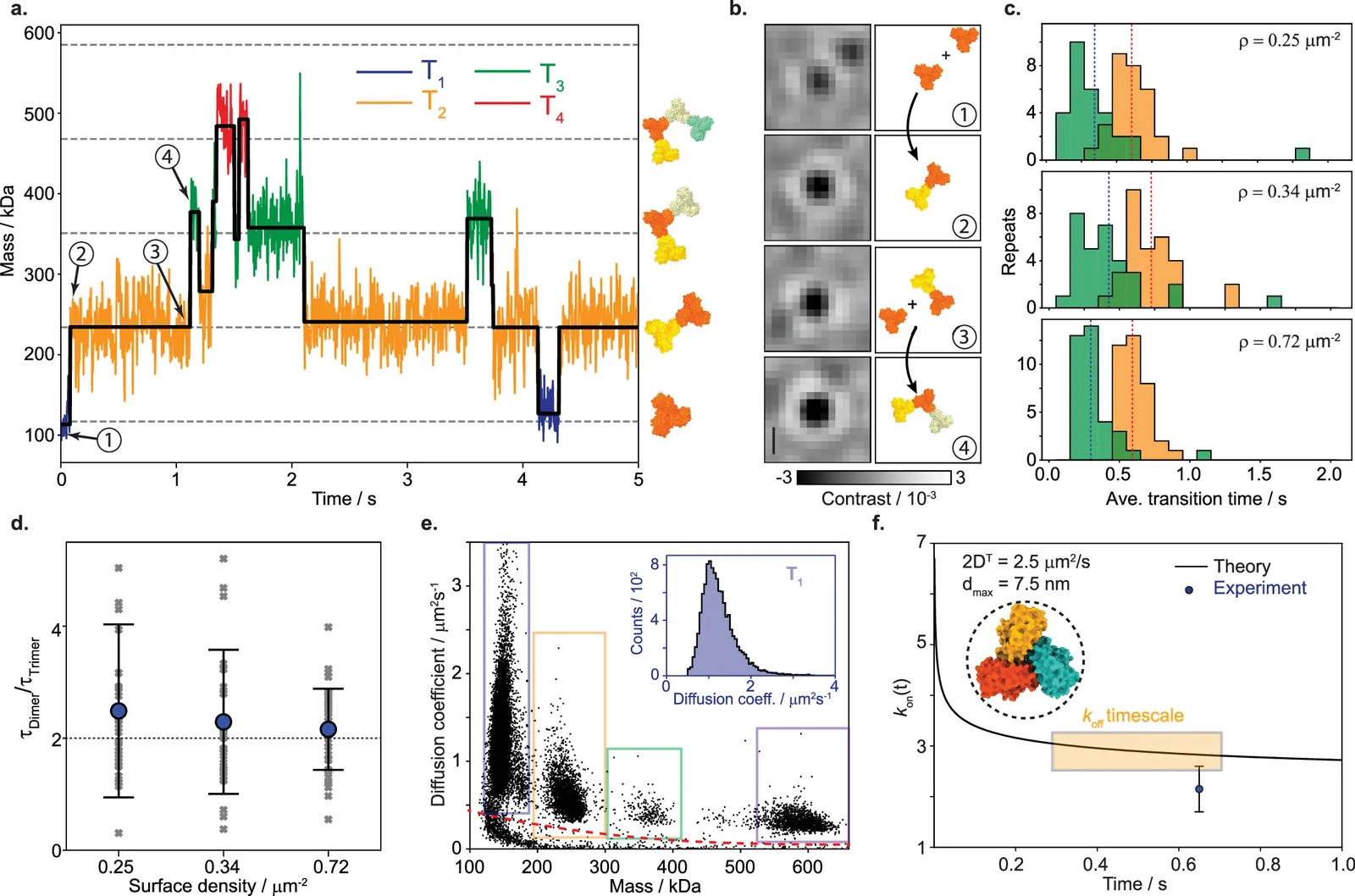
Diffusion-limited and independent pairwise interactions guide the assembly of the pentamer of mi3-trimers. **a**, Continuous mass monitoring during individual trajectories (coloured; Supplementary Video 1) and corresponding outcome of a step-finding algorithm (black curve; Methods, Supplementary Note 4 and Extended Data Fig. 2). **b**, Zoom of the ratiometric video showing dimer and trimer formation. Scale bar, 300 nm. **c**, Histograms of the average transition time for disassembly representing 30 independent 1-min dynamic MP videos. The average surface particle density of each set of experiments is indicated. Orange and green histograms correspond to the dimer and the trimer, respectively. **d**, The ratio between the averaged transition time of the dimer with respect to the trimer within the same measured video, calculated at different surface densities. Crosses indicate the measured ratio within individual videos and circles and error bars represent the averages and standard deviations, respectively. **e**, The molecular mass and diffusion coefficient measured for individual oligomers (scattered symbols). The area below the dashed red line indicates noise contributions from the SLB. Inset, the diffusion coefficient distribution measured for mi3-trimers. **f**, The mobility information together with the calculated dimensions of the molecular species (PDB ID: 7b3y) yields the theoretical, diffusion-limited, two-dimensional forward reaction rate constant, ${k}_{{\rm{on}}}^{2{\rm{D}}}(t)$ for the formation of dimers of trimers (Supplementary Note 3.3).

**Extended Data Fig. 2:**
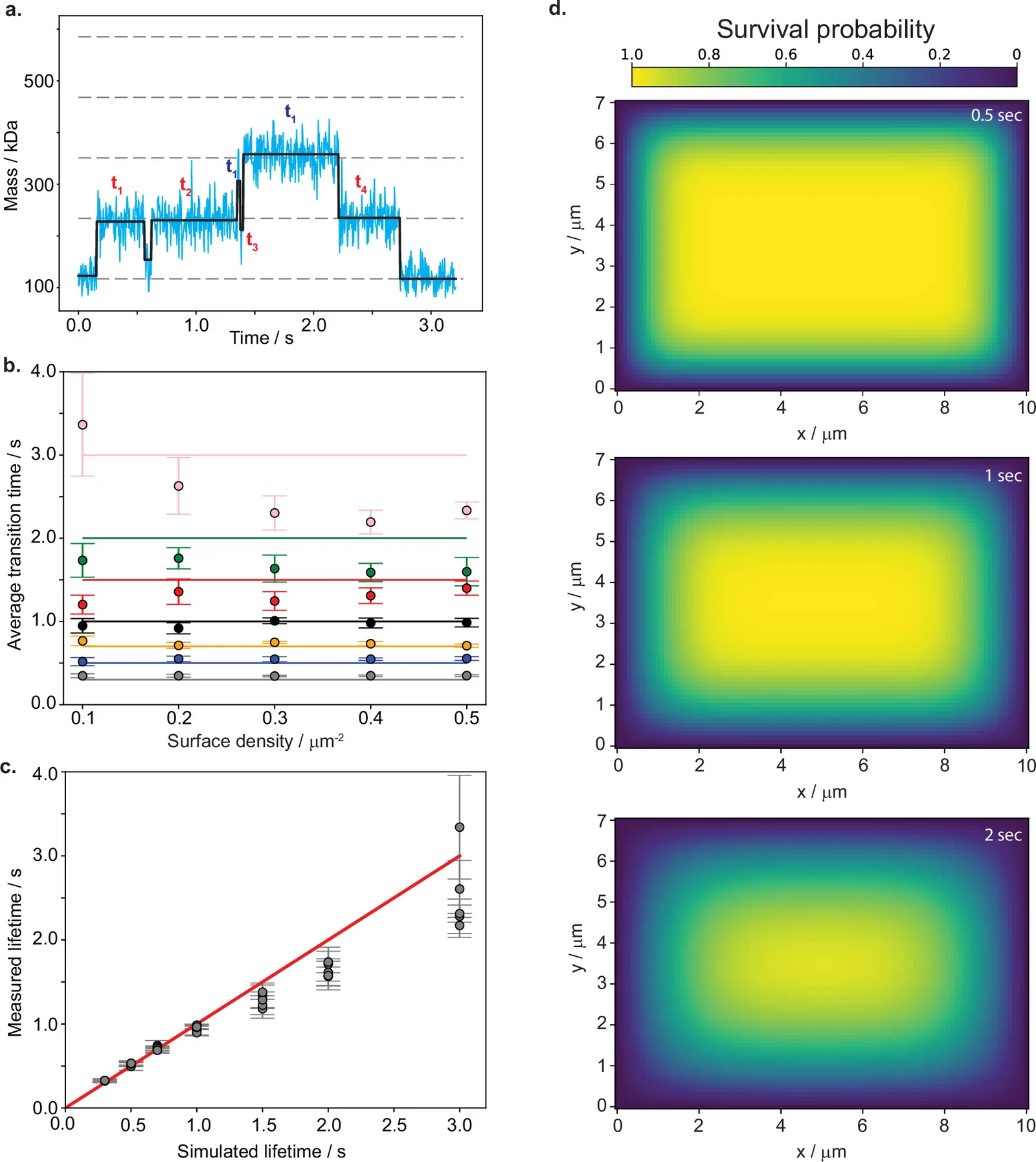
Step detection and validation of the average transition times with simulations. **a**, Representative example of segmenting a molecular mass trajectory (blue curve) into discrete molecular states (black curve). Red and blue time indices indicate the dwell times for the dimer and trimer of mi3-trimers, respectively. Horizontal dashed lines indicate the expected masses of the oligomeric states from mi3-trimer to the pentagonal ring. **b**, Average transition times extracted from simulated dynamic MP videos (Supplementary Note 4) with defined transition rates for dissociation of dimers of mi3-trimers at different surface densities. Coloured horizontal lines indicate the simulated transition times and the scattered symbols and error bars represent the averages and standard deviations, respectively, of values extracted from analysing five simulated videos (Supplementary Note 4). The colours of the scattered symbols correspond to the horizontal line colours. **c**, Analysed average lifetime as a function of the simulated lifetimes. Scattered symbols and error bars indicate the average and standard deviation per surface density. **d**, Calculation of the probability of a dimer of mi3-trimers to stay in the field of view for the indicated (at the top right) time period as a function of its initial position. The diffusion coefficient used for the calculations was set to the measured value of 0.6 μm^2^ s^−1^. The survival probability limits the accuracy of the calculated dwell time at longer timescales.

SLB tethering of subunits enables continuous monitoring of the molecular masses, positions, mobilities and interactions between individual subunits (Extended Data Fig. 1a,b), a measurement that cannot be performed in solution owing to rapid diffusion. Simultaneous tracking of molecular mass and position allows each association and dissociation event to be identified directly, providing both the reaction coordinate and the molecular state. We measured the average dissociation dwell times for the dimers and trimers of mi3-trimers, with transition times of 0.65 ± 0.05 s and 0.36 ± 0.04 s, respectively (Fig. 2f, Extended Data Figs. 1 and 2 and Supplementary Note 4). The factor of 2 between the dissociation times strengthens the suggested independence between the pairwise interactions (Extended Data Fig. 1d). Combining these measurements with the measured mobilities (Supplementary Fig. 8) and the equilibrium constant obtained from our thermodynamic analysis yields the complete elementary reaction dynamics governing the formation of the pentameric face, including the bimolecular association rate constants (Extended Data Fig. 1e,f and Supplementary Note 3.3). The values obtained show that association between the trimeric subunits is essentially diffusion-limited (Extended Data Fig. 1f), with no appreciable energetic barriers for association, and, as a result, that sampling the correct orientation between the two trimeric subunits is most likely the limiting factor.

## Assembly proceeds via topologically stable states

**Fig. 3:**
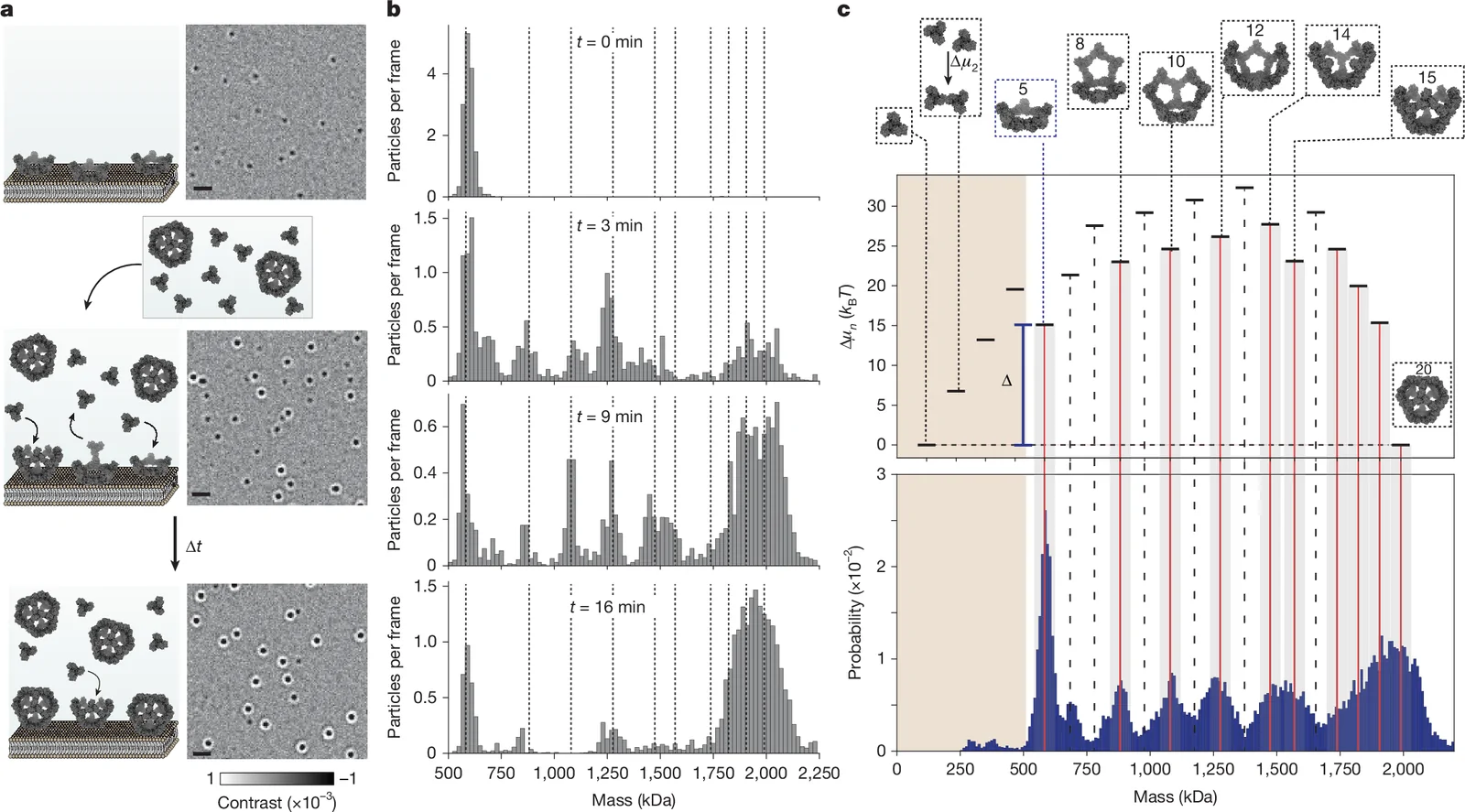
Stable intermediates reveal the grand canonical free energy landscape of VLP assembly. **a**, Driven VLP assembly on SLBs. Surface-assembled pentameric rings (top) are put in contact with equilibrated mi3-trimers and VLPs in solution (middle), which leads to VLP assembly on the SLB (bottom). Scale bars, 1 µm. **b**, Representative mass histograms as a function of time after addition of 44 nM mi3-subunits in solution. Dashed vertical lines correspond to the masses of closed-structure intermediates. Mass broadening, owing to particle size, for masses >1,750 kDa prevents resolving individual molecular states. Mass histograms arise from 507, 850, 823 and 1,111 molecular trajectories that were further segmented, weighted by their observation time and normalized by the total observation time per video (Methods). **c**, Time-averaged mass distribution (between 4 and 15 min) and comparison with the calculated grand-canonical free energy landscape using interaction parameters from Fig. 1. Solid red lines correspond to the masses of the topologically closed structures, also illustrated based on PDB ID: 7b3y. Dashed lines, unstable configurations. The structures shown for the 12-mer and 14-mer illustrate the corresponding topological state. Different specific molecular arrangements (with identical topology) are possible, owing to their overall similar mass. The averaged mass distribution originates from 15 different videos yielding a total of 19,785 trajectories.

**Extended Data Fig. 3:**
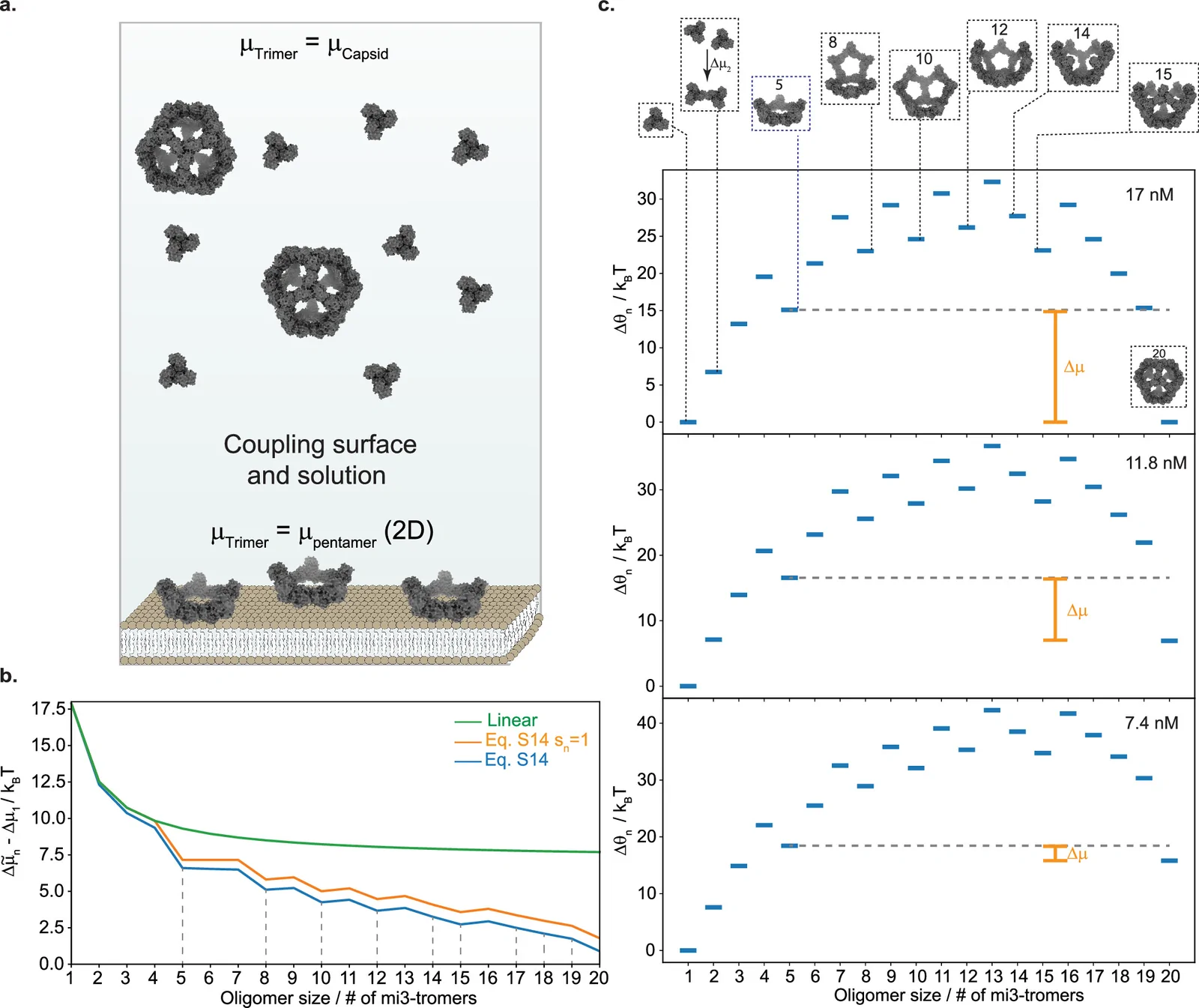
Calculation of the grand canonical free energy landscape. **a**, Illustration of the experimental procedure used to generate the driving force for the assembly reaction. The subunits on the SLB occupy their equilibrium distributions composed predominantly of pentagonal rings in chemical equilibrium with surface-tethered mi3-trimers. On addition of the equilibrated solution mixture, composed of soluble mi3-monomers, mi3-trimers and complete VLPs, assembly from the pentameric state becomes energetically favourable and the pentagonal rings on the surface are elongated through binding of mi3-trimers from solution. **b**, The chemical potential difference between an mi3-trimer within an oligomer of size *n* and a free mi3-trimer in solution, shown as a function of oligomer size for linear oligomers (green), VLPs without the degeneracy factor (orange) and VLPs oligomers with the degeneracy factor (blue) (see equation (S14) in the Supplementary Information). **c**, The normalized grand canonical free energy potential, ΔΘ_*n*_ (equation (S15) in the Supplementary Information) at three concentrations of mi3-trimers in solution, corresponding to the experimental mi3-monomer concentrations of 88, 44 and 29 nM. Although there is no driving force for assembly between the mi3-trimeric state and the complete VLP, coupling the solution and surface systems and initiating from the pentameric ring introduces a small driving force (orange). Illustrations depict the molecular structures of the topologically closed configurations.

By separating the assembly process into two phases, solution and SLB (Fig. 3a), we can create a modest driving force for assembly, while keeping the assembly conditions close to equilibrium. We achieve this by producing an equilibrated surface mixture of oligomers that contains closed pentameric rings, which serve as a nucleus for assembly. They represent the smallest stable oligomeric state with a higher probability to elongate than to dissociate. In solution, untagged mi3-subunits form an equilibrated mixture in which mi3-monomers, mi3-trimers and complete VLPs coexist (Fig. 1d). Because each phase by itself is equilibrated, there is no net driving force for assembly within each phase. However, coupling the two equilibrated systems introduces a chemical potential difference between the pentagonal ring and the complete VLP, mediated by the pseudocritical concentration of free mi3-trimers. In this coupled configuration, the chemical potential difference (Extended Data Fig. 3 and Supplementary Note 5) between the surface pentameric rings and the complete VLPs is constant, because the bulk concentration is effectively constant.

**Extended Data Fig. 4:**
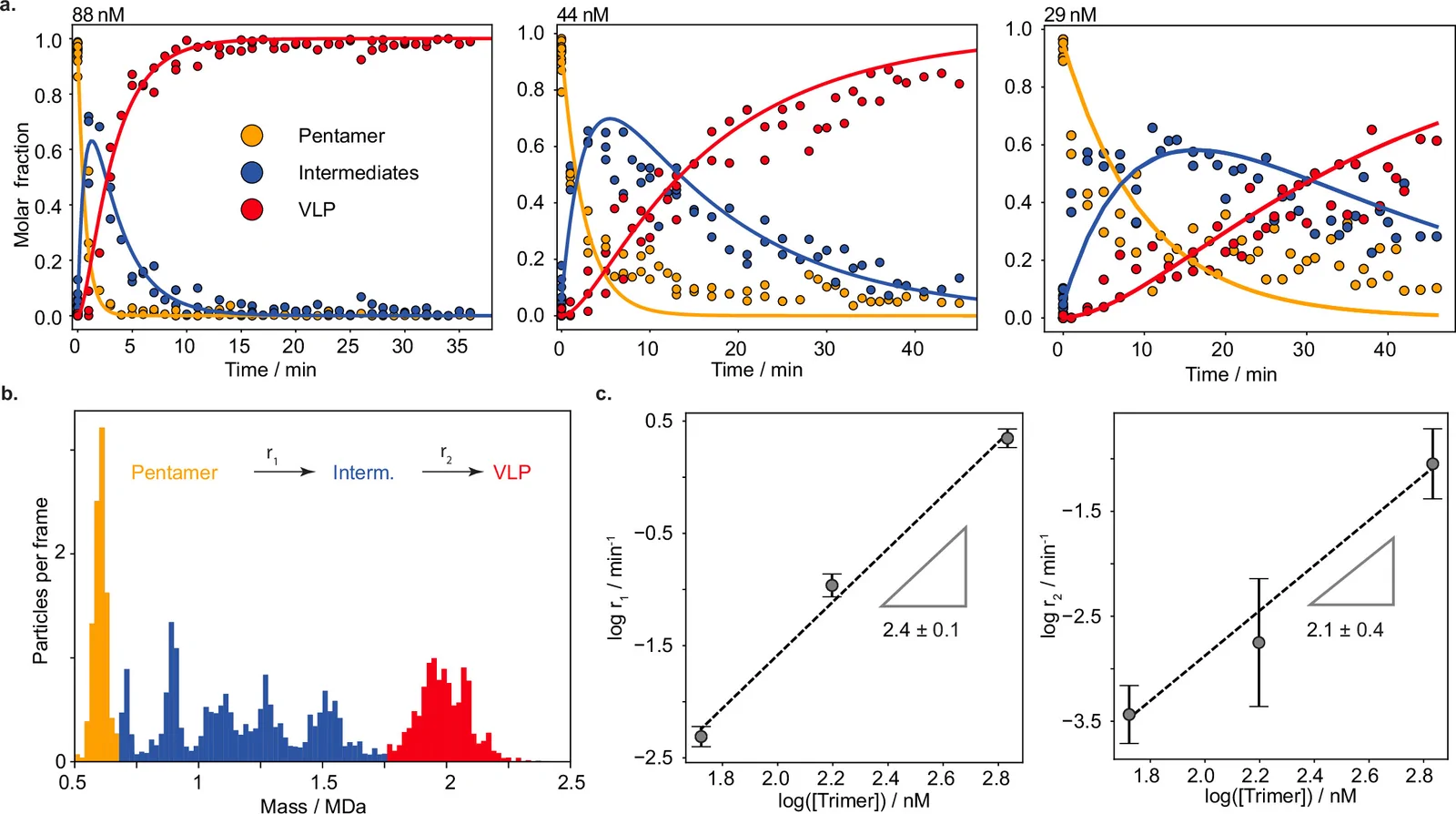
Clustering the bulk assembly dynamics into three states and fitting an irreversible kinetic model. **a**, Surface particle fractions as a function of time for the bulk assembly experiments described in Fig. 3 and Supplementary Note 6. Normalized particle fractions (symbols) were clustered into three molecular states: the initial pentagonal ring (‘Pentamer’, orange), intermediate structures (‘Intermediates’, blue) and complete/near complete VLPs (‘VLP’, red). The solution total concentrations of mi3-monomers were 88 nM (left), 44 nM (centre) and 29 nM (right). Curves at the corresponding colours indicate the best fitted kinetic model, considering two effectively irreversible consecutive reactions connecting these three states, assuming constant protein concentration in solution. **b**, Representative example of the clustering of the dynamic MP mass distribution presented in Fig. 3b and Supplementary Figs. 10–17 into the three molecular states. Legends indicate the expected irreversible kinetic model assuming constant protein concentration in solution and therefore constant effective rates. **c**, Scaling of the measured reactions rates (*r*_1/2_) with subunit solution concentration, shown on a logarithmic scale. The slope of the curve is indicated. Symbols and error bars correspond to the averaged fitted rates and their standard deviations based on 2–3 replicates for each assembly condition.

**Extended Data Fig. 5:**
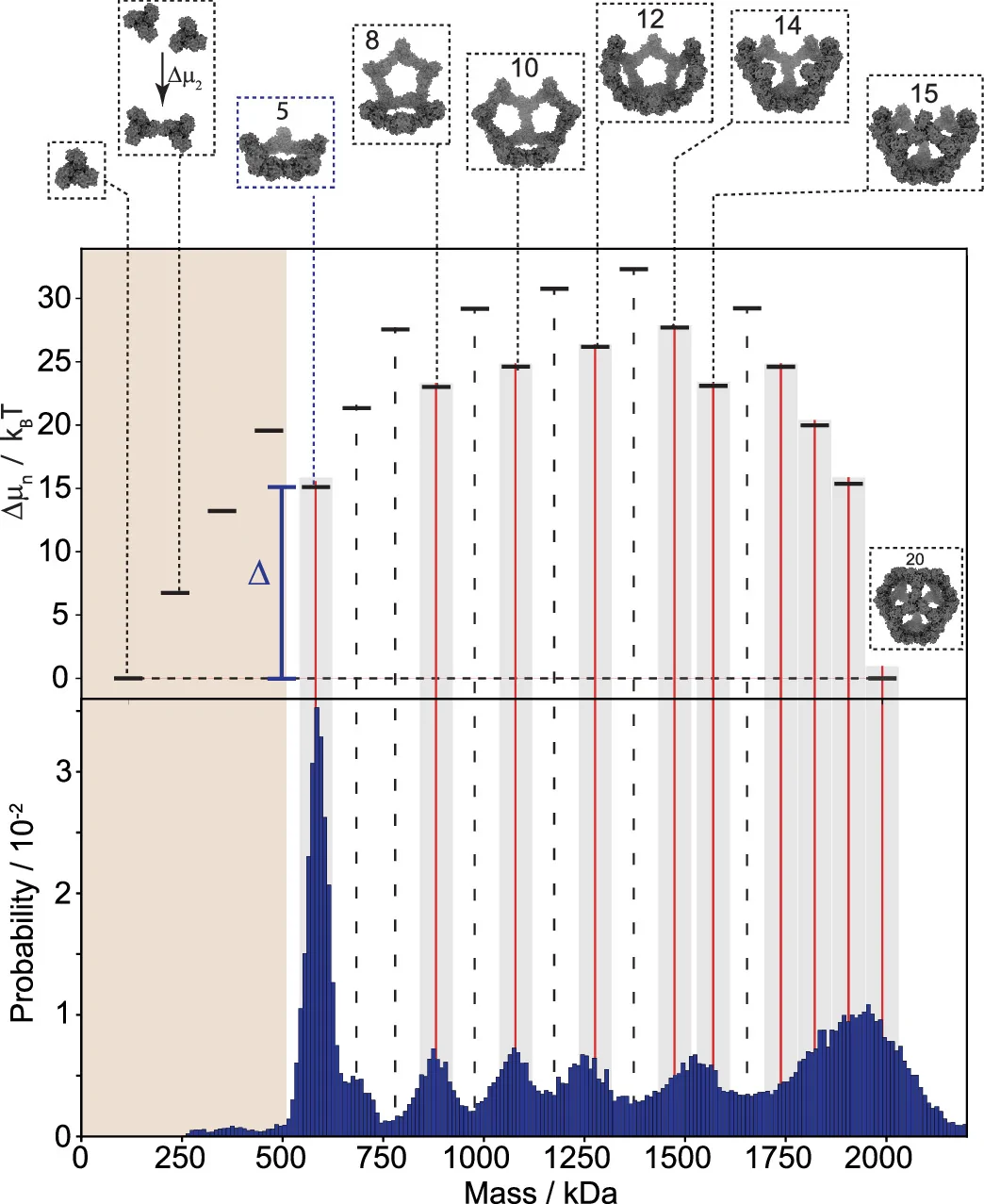
Comparing the bulk distribution of states for assembly at 29 nM mi3-monomer concentration with the free energy potential. Top part shows the same minimum grand-canonical free energy landscape shown in Fig. 3c. Topologically closed structures that occupy the local free energy minima are shown on the basis of the atomic structure of the VLP (PDB ID: 7b3y), indicated also by solid red vertical lines, whereas unstable configurations are indicated by dashed black vertical lines. The bottom part corresponds to summing the measured mass histograms (Supplementary Fig. 16) at intermediate time points along the assembly reactions (4–30 min) and for two technical repeats at 29 nM total protein concentration in solution.

The resulting small driving force (about 15 *k*_B_*T*), together with diffusion-limited and reversible inter-mi3-trimer interactions, result in a slow assembly reaction, enabling near-equilibrium sampling of the assembly propagation pathway. Notably, subunits in solution lack the histidine tag and therefore do not interact with the SLB (Supplementary Fig. 5). Any observed mass additions thus arise from subunits from solution binding to SLB-bound pentamers (Fig. 3b and Supplementary Figs. 10–18). Therefore, the assembly propagation is dictated by the 3D, solution free-energy landscape. Repeating the experiment at decreasing solution concentrations slows down the assembly timescale, ranging from about 10 min for complete assembly at 88 nM to about 20 min at 44 nM (Fig. 3b) and 40 min at 29 nM (Extended Data Fig. 4 and Supplementary Figs. 10–17). Although the timescales differ, the intermediate states (indicated in Fig. 3b, black vertical lines) are consistent across different concentrations. Averaging distributions across technical repeats during time periods in which relatively little change in intermediates is observed improves the statistics and reveals well-resolved peaks and valleys as a function of mass (Fig. 3c and Extended Data Fig. 5).

Overlaying the calculated free energy landscape of the lowest free energy path between the mi3-trimeric subunit and the complete VLP reveals the driving force for assembly following the formation of the pentagonal ring (Fig. 3c, top). It also shows that oligomeric states that accumulate along the assembly reaction match local minima in the free energy landscape, whereas low abundances correspond to local maxima (Fig. 3c and Extended Data Fig. 5). Examining the associated stoichiometries shows that stable intermediates are those corresponding to closed pentagonal faces (or closed rings), stabilized by a larger average trimer–trimer coordination number. It is important to note that the equilibrated grand canonical free energy landscape, representing the equilibrated solution, does not change when the two systems are coupled, only the starting point for assembly. Therefore, the measured distribution of oligomeric species on the SLB propagates in time according to the molecular transitions governed by the equilibrated underlying free energy potential.

## Single-particle dynamics show irreversible assembly 

**Fig. 4:**
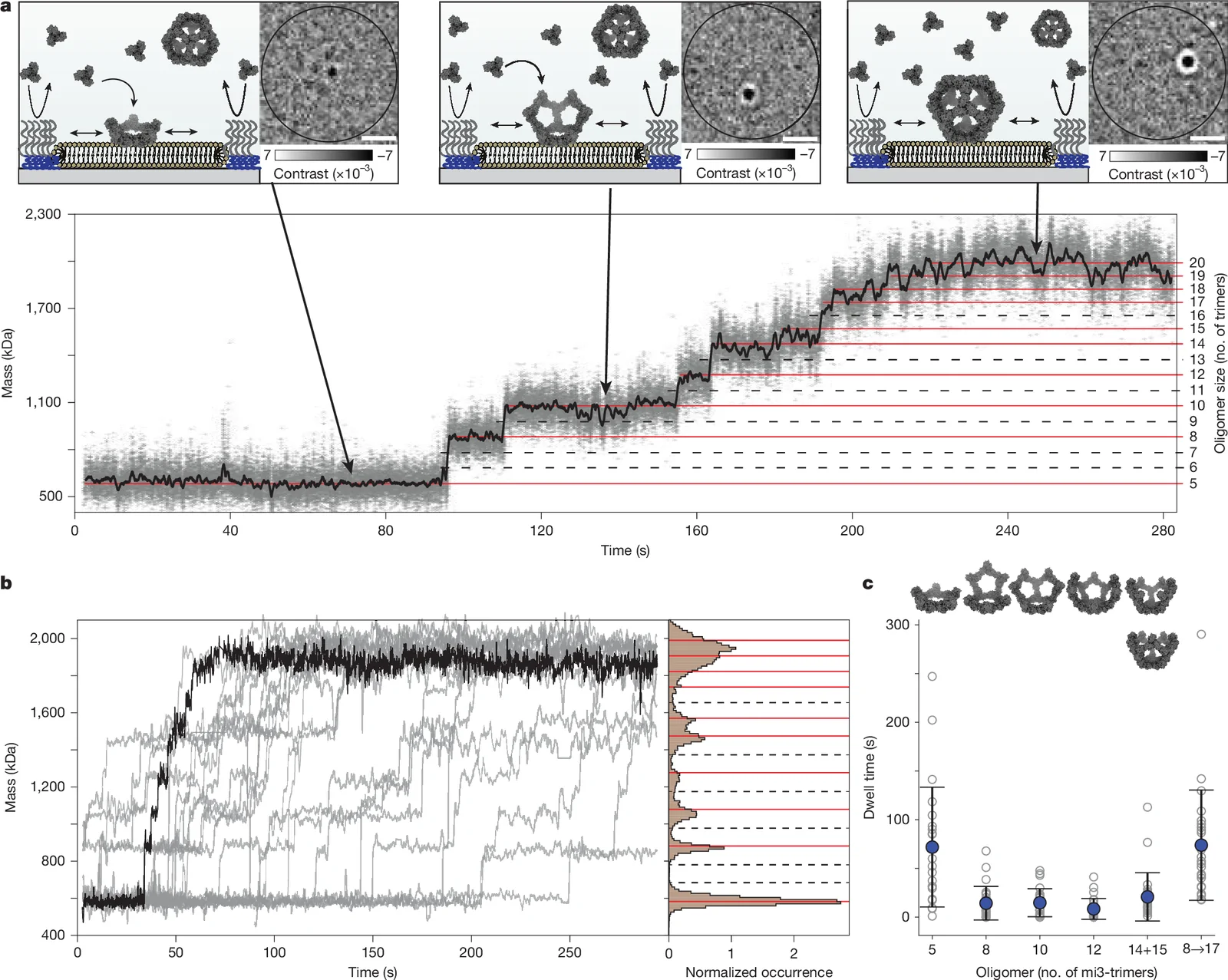
Single-molecule observation of self-assembly pathways and dynamics. **a**, Top, illustration of single complex MP by SLB traps. Scale bars, 1 µm. Bottom, mass trace for the assembly of a single VLP. Grey symbols show the measured mass at 250 Hz imaging frame rate (Supplementary Video 2) and the black line is a running median with a symmetric window size of 100 frames (400 ms). Solid red (topologically closed intermediates) and dashed black (unstable intermediates) horizontal lines denote the expected masses of complexes along the lowest free energy path from the pentameric state to the assembled VLP. **b**, Twenty-five superimposed mass traces capturing the assembly of individual VLPs. Traces are shown following the application of a running median with a window size of 100 frames (400 ms). Gaps in the mass traces correspond to transient immobilization of the particles (Supplementary Fig. 21). The histogram to the right shows the average normalized distribution of the sampled mass states. Horizontal lines correspond to the same states as in **a**. **c**, Observed dwell times for the most stable intermediates along the assembly path and the time interval between the formation of the first intermediate structure (8-mer) and the formation of a particle that is equal to or larger than 17 subunits (representing the transition through all intermediate states for the assembly process). The data were obtained from the 25 single-molecule trajectories shown in **b** (Supplementary Fig. 19). Grey symbols indicate the individual values from each trajectory and the blue symbols and error bars represent the averages and standard deviations, respectively.

**Extended Data Fig. 6:**
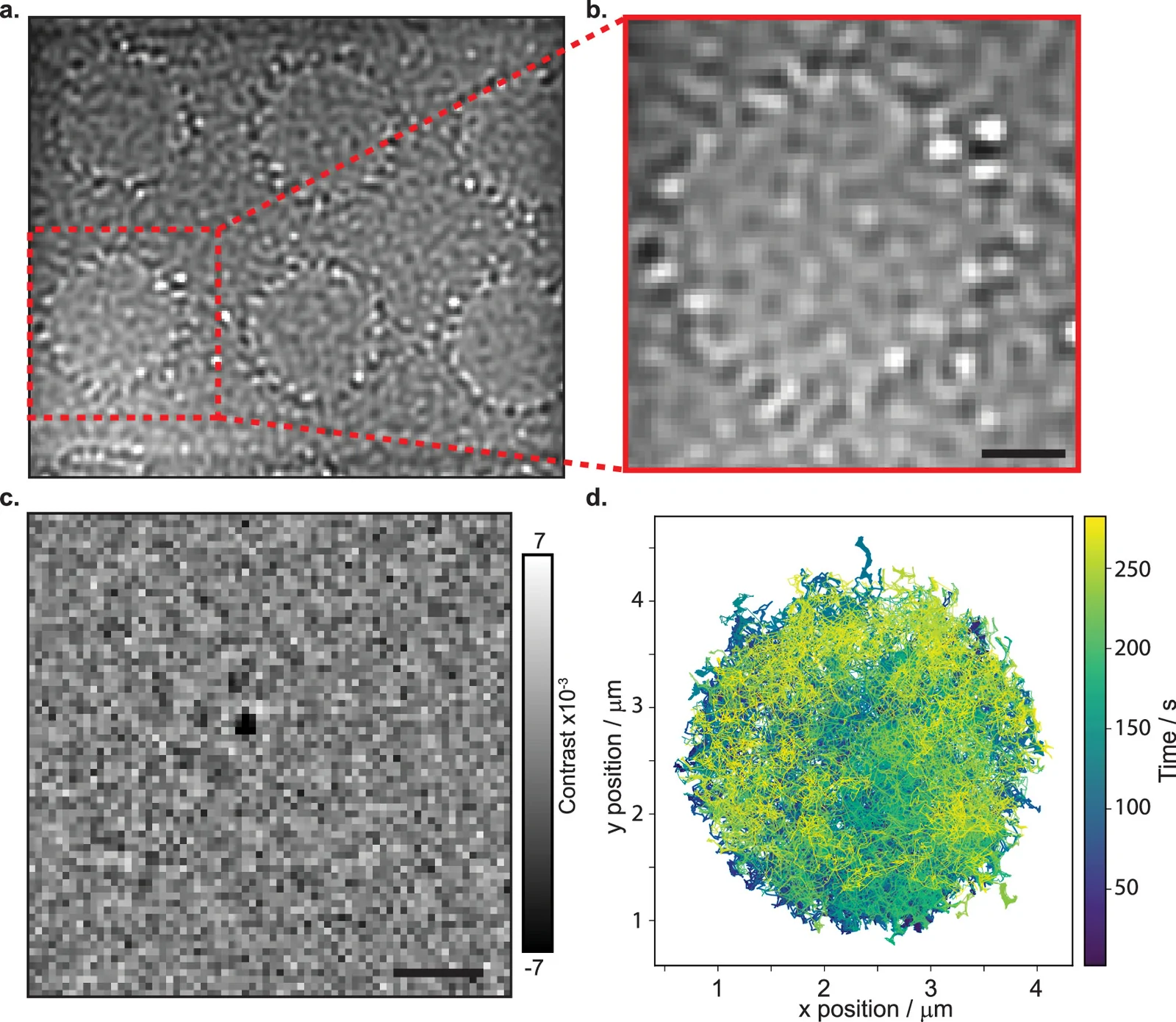
MP using confined SLBs. **a**, MP image of the confined SLB traps measured with a field of view of 15.4 × 13.2 μm^2^. **b**, MP image of an individual circular trap. Scale bar, 1 μm. **c**, Median processed MP image of the same region, showing a diffusing mi3 pentamer of trimers in its centre. **d**, Trace of the *x*,*y* positions of a single diffusing assembling complex during 5 min of measurement at 250 Hz.

Population-level measurements highlight overrepresented states and thus hint at key assembly intermediates. From an ergodic standpoint, repeated measurements of one complex should converge to the ensemble probability distribution. However, the ensemble does not provide information on the sequence of steps or the transition dynamics. We therefore combined MP with a single-molecule confinement approach^53^ to observe the real-time assembly dynamics of VLPs at the level of individual complexes with molecular resolution. To achieve this, we create circular SLBs of 5 µm diameter by lithographic removal of a dense poly-L-lysine polyethylene glycol (Fig. 4a, top) layer. Any species bound to the SLB are now confined and can thus be observed and mass measured for extended periods of time (Extended Data Fig. 6).

We form SLB-bound pentameric rings by adding 6xHis-tagged mi3-subunits as before but now to SLB traps, followed by approximately 30 min of equilibration (Methods). Subsequently, we add an equilibrated solution of untagged mi3-subunits at total protein concentrations between 88 and 100 nM. Mi3-trimeric subunits from solution bind to tethered pentameric rings, which we monitor in real time by continuous mass measurement (Fig. 4a and Supplementary Video 2). The resulting mass traces reveal the oligomeric states that are populated for extended time periods, separated by sharp mass changes. Intermediate masses match those of topologically closed structures (red lines), which also accumulate in ensemble experiments (Fig. 3c). We observed these features irrespective of assembly rate, for which both slower (Fig. 4a, top) or faster (Fig. 4b, black curve) molecular mass trajectories exhibited similar features.

Superimposing multiple trajectories (Fig. 4b) confirms these observations, for which the normalized distribution exhibits features similar to the ensemble results (Fig. 3c), linking the single complex dynamics experiments (Fig. 4b) to population mass distributions (Fig. 3c). Although the rapid off-rate combined with the small mass change (108 kDa) associated with pairwise trimer–trimer interactions (Fig. 1) is beyond the capabilities of our present experimental approach, we can observe and identify the key states with longer dwell times, that is, those representing closed structures. Notably, we do not observe disassembly in any of the measured trajectories.

**Extended Data Fig. 7:**
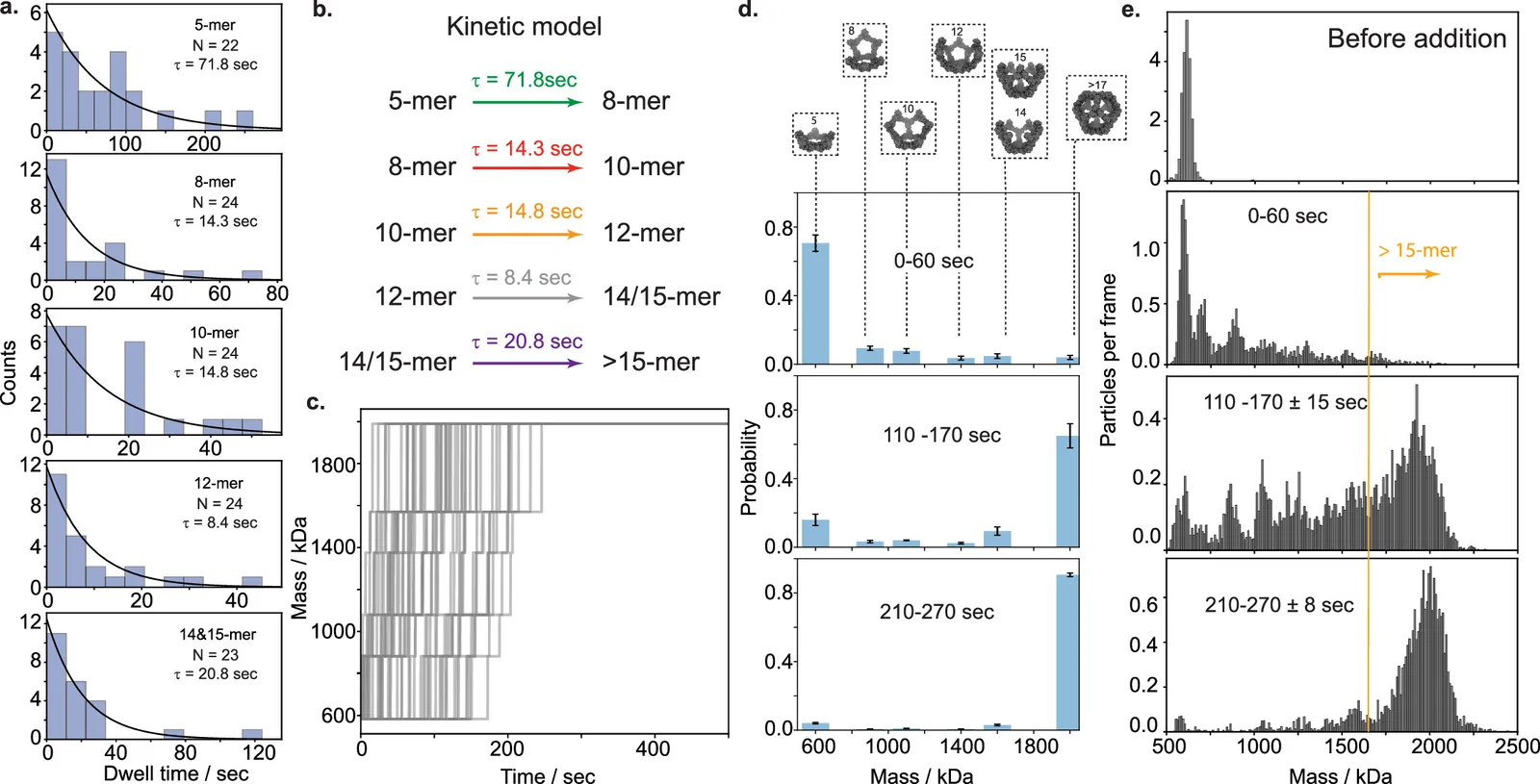
Single-complex transition dwell times. **a**, Measured state-dependent dwell times distributions extracted from the assembly traces of individual mi3-VLPs. For each state, the number of trajectories and the averaged dwell time are given. The black curve corresponds to the expected exponential distribution given the calculated average. **b**, Representation of the six states model, kinetic Monte Carlo simulation used to simulate the relative occupancies of the different assembly states as a function of time, given the measured averaged transition time. **c**, An example of the simulation outcome, showing the stochastic propagation of states for 50 tested trajectories. **d**, The expected probability to sample each state during 60-s intervals at different times during the process. Probabilities were calculated on the basis of 500 traces (ten independent simulations rounds, containing 50 trajectories each). **e**, Assembly reaction on continuous SLBs performed at 88 nM of subunits in solution (see Fig. 4 for experimental description and Supplementary Figs. 10–12 for the complete time trace at 88 nM solution concentration). Histograms represent averaged distributions over three independent measurements at similar time points following the addition of solution subunits.

When evaluating the lifetimes associated with different intermediates (Supplementary Figs. 19 and 20), the pentameric ring exhibited the longest dwell time, whereas other species showed broadly similar dwell times (Fig. 4c and Extended Data Fig. 7a). This is readily explained from a molecular perspective: transitioning from the pentagonal ring to the next stable state (8-mer) requires binding of three weakly interacting mi3-trimers to form an extra topological face before any of them dissociates. The subunit concentration in solution determines the collision frequency and therefore the probability that three subunits bind in the correct configuration before unbinding. By contrast, for subsequent transitions up to the 14-mer, closing each new face requires only two more subunits, leading to shorter transition times. Using the measured dwell times (Fig. 4c), we simulated the expected time-dependent oligomeric distribution of the closed configurations at a mi3-monomer concentration of 88–100 nM. The resulting population evolution is in good agreement with the independently measured population-level distribution acquired at a comparable concentration (Extended Data Fig. 7b,c), showing a direct connection between the stochastic dynamics of individual assembly trajectories and the behaviour of the ensemble. We note that the reported pentamer dwell time may be affected by the approximately 20 s gap between subunit addition and the start of acquisition, as well as the maximum observation time of 300 s (Supplementary Fig. 22). Because the 14- and 15-mers are both closed structures, we report the cumulative dwell time (top of Fig. 4c).

## Discussion

**Extended Data Fig. 8:**
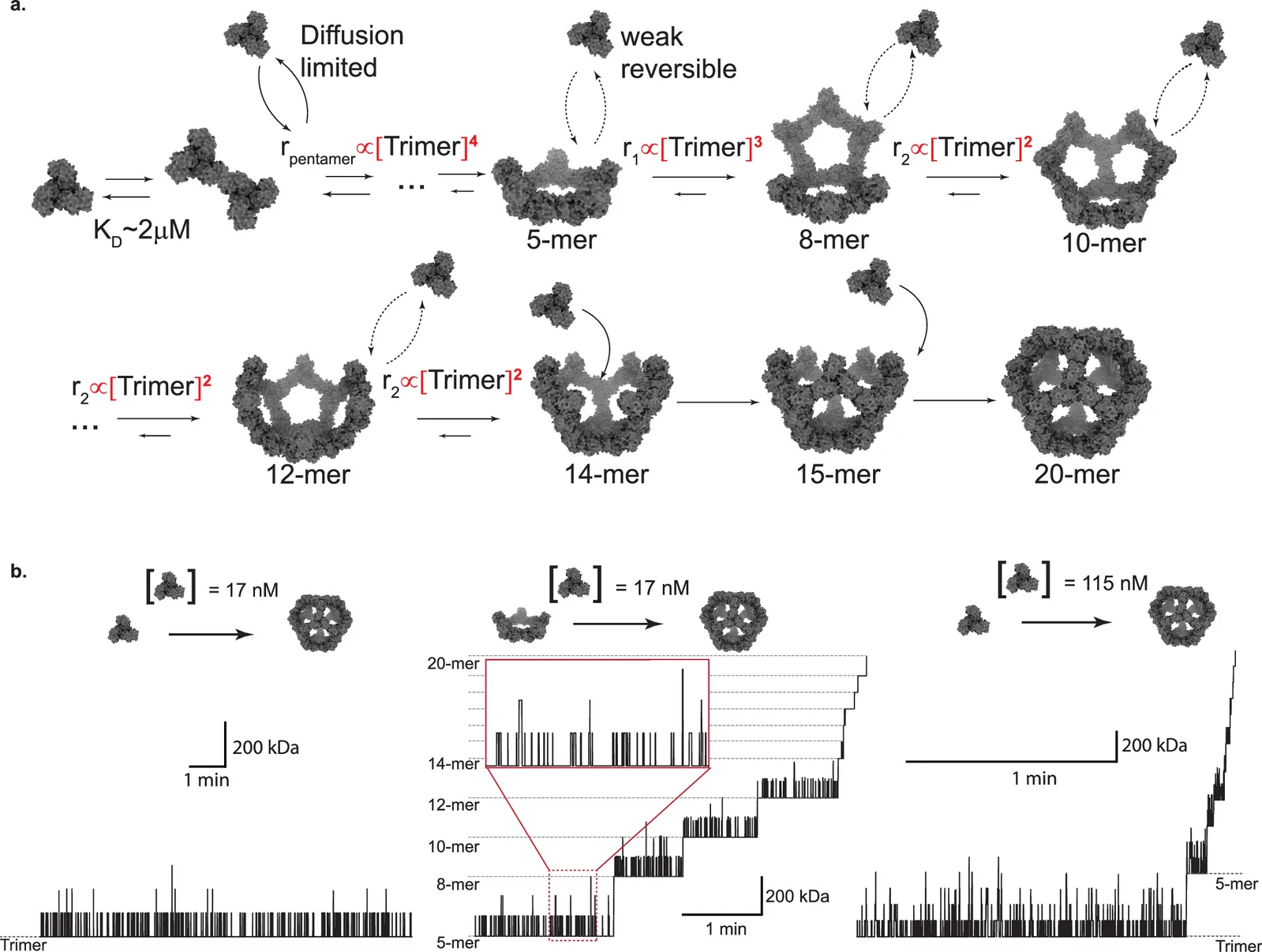
Molecular mechanism of VLP assembly. **a**, Quantitative illustration of the assembly mechanism based on the combined bulk and single-molecule results. **b**, Stochastic simulation of the assembly process assuming the parameters *k*_on_ = 10^7^ M^−1^ s^−1^ and *k*_D_ = 1 μM. Left, representative simulated trace starting from a trimeric subunit, at a trimer solution concentration of 17 nM. Middle, starting from a pentagonal face, individual subunits from a solution containing 17 nM of trimers bind weakly and reversibly until they randomly bind in a configuration that allows the formation of a ring structure that cannot disassemble. The assembly process proceeds through these stable states. Right, kinetics of VLP assembly with the same molecular parameters. This time, the starting point is a trimeric subunit and the concentration in solution is 115 nM.

Our measurements reveal the molecular, energetic and kinetic details of a topology-driven, self-limiting, VLP assembly pathway (Extended Data Fig. 8a). Trimeric subunits engage in weak pairwise interactions (*K*_D_ ≈ 2 μM) allowing for reversible encounters, which stochastically explore molecular configurations until a topologically closed stable arrangement is reached. Notably, the short lifetime of individual interactions prevents the persistence of loosely connected structures. Once a subunit attains a coordination number ≥2, the associated stabilization results in a much slower off-rate than the on-rate for new subunits arriving. Repetition of this process throughout the growth of the dodecahedron enables an efficient and well-defined assembly process.

Our results provide a molecular-level explanation to the kinetic origin of the difference between nucleation and elongation, the hallmark of a two-state assembly process (Fig. 1) and characteristic of many capsids and VLPs^32,33,62^, for which the nucleus was suggested to be a small polygon intermediate^48^. Here the smallest stable intermediate is a pentagonal ring, formed after four trimer additions. The subsequent transition (5-mer to 8-mer) requires three additions, whereas later steps up to 14-mer require only two. Our measurements (Fig. 2) suggest that the interactions are independent and therefore we expect that the corresponding reaction rates scale with the fourth, third and second powers of the mi3-trimer concentration, respectively (Extended Data Fig. 8a). Owing to the irreversibility of the closed topologies, transitions between these configurations can be viewed as a series of first-passage stochastic events. Because the first-passage time is highly sensitive to the number of additions, formation of the first pentagonal face is the critical step: once formed, it enables propagation of the entire assembly process, with sequentially increasing rates. The distinction between nucleation and elongation thus arises directly from the difference in the number of subunits required to complete topological rings, corresponding to a sequence of first-passage events.

Observation of single VLP assembly events shows that, within the timescale of assembly, the reaction proceeds through a sequence of irreversible, specific steps. This creates an apparent contradiction between microscopic irreversibility that predicts total consumption of mi3-trimeric subunits, whereas our solution data (Fig. 1) shows coexistence between subunits and complete VLPs, consistent with the law of mass action^63,64^. Key here is that, although specific states can be considered as irreversible, the pairwise interactions leading to these states are weak and reversible. Thus, while the assembly reaction proceeds and the concentration of free subunits decreases, the timescale for creating the pentameric intermediate rapidly increases. At the pseudocritical monomer solution concentration, which is eventually reached independent of the starting concentration as VLPs are assembled, this timescale becomes either so long that assembly is practically irrelevant or comparable with the timescale of ring disassembly (longer than our observation window). In both cases, experimentally, the result complies with the expected observation of the law of mass action, in which subunits and complete VLPs coexist in solution.

We can now understand the assembly process in full detail, represented by a simple stochastic kinetic simulation (Supplementary Note 7). At sufficiently low concentrations, lower or equal to the pseudocritical mi3-trimer concentration (17 nM; Fig. 1d), the probability of five trimers coming together before at least one of them departs becomes vanishingly low. As a result, the pentagonal ring is never formed and thus, also, no VLPs (Extended Data Fig. 8b, left). If, however, we skip this low-probability initial pentagonal ring formation step, here by lowering the barrier using an SLB, assembly does become possible at the same solution concentration (Extended Data Fig. 8b, middle and Fig. 3). Finally, at concentrations that result in a high assembly yield, the timescale difference between ring formation and subsequent propagation makes intermediates so transient that, at equilibrium, only free subunits and complete VLPs will coexist (Extended Data Fig. 8b, right and Fig. 1b,c).

Our work provides experimental access to fundamental assembly mechanisms of empty VLPs, showing how weak, reversible, multivalent interactions lead to nucleation-like behaviour through a restricted set of highly coordinated, productive intermediates. Further regulatory factors, including conformational regulation and shell elasticity are nevertheless expected to further shape assembly pathways of native capsids and capsid-like structures. These have been proposed to play important roles in capsid polymorphism^65^ and in affecting assembly outcomes in response to small-molecule assembly modulators^27^ and will be the subject of future work using our approach. Beyond the implications for our understanding of the fundamental principles driving and controlling self-assembly, our approach represents a framework for studying biomolecular assembly and dynamics more broadly. The approach of characterizing pairwise interactions, quantifying equilibrium distributions and monitoring individual assembly events and dynamics is general: here it was applied to a simple, homo-oligomeric analyte, but owing to the universality of mass measurement, our approach can be applied to a broad range of multimolecular systems, opening up new avenues for studying biomolecular dynamics and mechanisms in the context of biological function and regulation.

## Methods

### Expression and purification of SC003-mi3 VLP

The mi3-VLP (also known as SC003-mi3) was derived from i3-01 (ref. ^66^) by introducing two mutations that remove surface-exposed cysteines to prevent aggregation and by genetically fusing a SpyCatcher domain to the N-terminus to enable plug-and-play attachment of SpyTagged proteins^54^. The plasmid pET28a-SpyCatcher003(SC003)-mi3 (gift from Professor Mark Howarth, Cambridge University) was transformed into *Escherichia coli* BL21(DE3) RIPL cells (Agilent) and plated on Luria-Bertani (LB) agar supplemented with 50 µg ml^−1^ kanamycin. After incubation for 16 h at 37 °C, a single colony was used to inoculate 10 ml LB media containing 50 µg ml^−1^ kanamycin and grown overnight at 37 °C with shaking at 200 rpm. This starter culture was transferred into 1 l LB medium with the same antibiotic and incubated at 37 °C, 200 rpm, until the OD600 reached about 0.6. Protein expression was then induced with 0.42 mM IPTG and cultures were grown for a further 16 h at 22 °C with shaking (200 rpm). Cells were collected by centrifugation at 4,000 × *g* for 15 min. Cell pellets were resuspended in 40 ml lysis buffer (20 mM Tris-HCl, 300 mM NaCl, pH 8.5 at 4 °C) containing 0.1 mg ml^−1^ lysozyme, cOmplete EDTA-free protease inhibitor cocktail (Roche, 1 mg ml^−1^) and 1 mM PMSF. The suspension was passed through a high-pressure homogenizer (Constant Systems) at 30,000 psi, with 2–3 passes on ice. The lysate was clarified by centrifugation at 35,000 × *g* for 45 min at 4 °C and the supernatant was collected. Ammonium sulfate was then added at 170 mg/ml of lysate and the mixture was incubated at 4 °C for 1 h with agitation (220 rpm) to precipitate the particles. Following centrifugation at 30,000 × *g* for 35 min at 4 °C, the pellet was resuspended in 8 ml buffer (25 mM Tris-HCl, 150 mM NaCl, pH 8.0 at 4 °C) and passed through 0.22-µm filters (Croning). The filtrate was dialysed overnight against a 500-fold excess of the same buffer at 4 °C. Dialysed material was centrifuged at 17,000 × *g* for 30 min at 4 °C to remove insoluble aggregates and filtered again (0.22 µm). Purification was completed by size-exclusion chromatography on a HiPrep Sephacryl S-500 HR 16/60 column (GE Healthcare) equilibrated in 25 mM Tris-HCl, 150 mM NaCl, pH 8.0 at 4 °C, using an ÄKTA Pure 25 system (GE Healthcare). Elution was performed at 1 ml min^−1^, collecting 1-ml fractions. Fractions containing SpyCatcher003-mi3 nanoparticles were pooled, concentrated and dialysed into TBS 25 mM Tris-HCl, 150 mM NaCl, pH 8.0 at 4 °C using a 100-kDa MWCO centrifugal filter (Millipore) and stored at −80 °C. Final protein concentration was determined by BCA assay (Pierce, Thermo Fisher Scientific).

#### Solution self-assembly experiments

It was shown previously^66^ that mi3-VLPs can reversibly disassemble and reassemble at guanidinium thiocyanate concentration of 2.5 M. To perform the assembly experiments, we started with a solution containing purified mi3-VLPs (Supplementary Fig. 1) at an mi3-monomer concentration of 84 μM and disassembled the VLPs using 2.5 M of guanidinium thiocyanate at varying mi3-monomer concentrations ranging from 35 to 5.8 μM. Following an incubation time of 30 to 60 min at room temperature, assembly was initiated by rapidly diluting the protein solution 100-fold in assembly buffer (20 mM HEPES pH 7.4, 138 mM NaCl) to a final mi3-monomers concentration ranging between 350 and 58 nM. The assembly reactions equilibrated at room temperature for an extra 30 min. We quantified the distribution of masses of the reassembled VLPs at the chosen concentrations using a standard MP landing assay.

#### MP measurements in solution

The equilibrated assembly reactions (Fig. 1 and Supplementary Figs. 2 and 3) were measured using a commercial mass photometer (TwoMP, Refeyn Ltd.) using an imaging field of view of 4.3 × 10.9 μm^2^. Measurements were conducted on microscope glass coverslips (24 × 50 mm, Menzel Gläser, VWR 630-2603) that were pre-cleaned by three consecutive 5-min cycles of bath sonication in acetone, 50% isopropanol in Milli-Q water (18.2 MΩ cm) and Milli-Q. Cleaned coverslips were then dried using nitrogen flow and 3-mm silicone gaskets (GBL103250, Grace Bio-Labs) were attached to the coverslip surface. The gasket was prefilled with 15 μl of buffer and the focus position was adjusted for maximum contrast before adding 5 μl of protein solution. Measurements were performed at a frame rate of 500 Hz followed by frame binning of 2, resulting in an effective frame rate of 250 Hz. We analysed data using DiscoverMP v2024R1 (Refeyn Ltd.), in which rolling ratiometric videos were generated using an averaging window size of 20 frames (80 ms). Threshold parameters for particle detection were set to the default values of 1.5 (threshold 1) and 0.25 (threshold 2). For each dataset, a calibration of ratiometric contrast to mass was performed using a protein standard while using the same acquisition parameters, similar to a previously reported procedure^60,67^.

#### SLB preparation

SLBs were prepared using a similar procedure as previously reported, with small modifications^21^. In short, phospholipid stocks in chloroform were mixed to form a 5 mM stock solution with a molar composition of 0.05 mM DGS-NTA, 0.1 mM 18:1 PEG550 and 4.85 mM POPC. The stock solution was stored at −20 °C. Before use, 50 μl of the lipid stock solution was added to 200 μl of chloroform in a clean glass tube. The chloroform was evaporated by manually rotating the tube while applying a weak flow of nitrogen, followed by 1 h of evaporation under vacuum. Lipids were hydrated by adding 0.5 ml of buffer (20 mM HEPES pH 7.4, 150 mM KCl), followed by two cycles of 20-min incubation in a 40 °C water bath, mixing between each cycle. The sealed tube was left at ambient room temperature for at least 2 h or overnight. The hydrated lipids were tip-sonicated in a 1.5-ml Eppendorf tube using a 2-mm tip probe at 30% power and 1 s pulse duration separated by 3 s waiting time for a total of 10 min sonication time (Vibra-Cell, Sonics & Materials). During sonication, the tube was kept in ice water. The sonicated lipids were centrifuged at 21,130 × *g* for 30 min at 4 °C, before taking 0.4 ml of the supernatant. Cleaned coverslips were treated with oxygen plasma for 5 min at 40% power and 0.6 mbar oxygen pressure (Zepto plasma cleaner, Diener Electronic). Immediately after plasma cleaning, a silicon gasket (GBL103280, Grace Bio-Labs) was placed at the centre of the coverslip and 30 μl of buffer (20 mM Tris pH 7.8, 150 mM NaCl, 2 mM MgCl_2_) followed by 20 μl of lipids were added and thoroughly mixed in the gasket and SLB formation was allowed for about 20 min. After examining the SLB integrity, excess vesicles were washed from the surface with assembly buffer.

#### Preparation of the histidine tag mi3-VLPs for measurements on SLB

Spytag-polyhistidine peptide (Spy-hist) at a concentration of 270 μM in DPBS was mixed with an 84-μM solution of mi3-VLP (total mi3-monomer concentration) at a volume ratio of 2:1, resulting in a large excess of the Spy-hist peptide (180 μM versus 27 μM) and the mixture was incubated on ice for 3 h. Following incubation, the solution was filtered through a 4-ml, 100-kDa MWCO centrifugal filter (Amicon) at 4,000 × *g* eight times to remove the excess peptide. For each round of centrifugation, the 4-ml initial solution was concentrated to 0.1 ml. This resulted in an estimated dilution factor for the excess of Spy-hist peptide of 10^9^. To tether the subunits to the SLBs, the tagged VLPs were disassembled by diluting 1 μl of the tagged VLP solution into 50 μl of 2.5 M of guanidinium thiocyanate. After about an hour, the disassembled VLPs were rapidly diluted 100-fold into 20 mM HEPES pH 7.4, 138 mM NaCl (assembly buffer). The final concentration of tagged mi3-monomers is estimated to be 5 nM, which is much lower than the critical concentration for VLP formation. MP measurements validated the existence of only mi3-monomers and mi3-trimers in solution, before addition as a solution on top of the SLBs.

#### Dynamic MP acquisition and data analysis (Figs. 2 and 3)

*Data acquisition*. Dynamic MP measurements of the two-dimensional assembly reactions of the pentagonal face were performed on a commercial mass photometer (OneMP, Refeyn Ltd.). We used the ‘medium’ field of view (6.3 × 9.9 μm^2^) at the maximum frame rate of 540 Hz and a metapixel size of 77.35 nm after 4 × 4-pixel binning. After frame averaging (two frames), the effective frame rate was 270 Hz. Following the formation of the SLB, 2 nM of tagged subunits were added to the gasket. Through different incubation times, the density of trimeric mi3-subunits was controlled. When the desired density of particles was obtained, the solution was replaced with assembly buffer at least five times, washing away soluble mi3-subunits, and the system was allowed to equilibrate for 30 min. For characterizing the thermodynamics (Fig. 2), after equilibration, between 3 and 15 60-s-long MP videos were acquired to collect enough statistics of particle trajectories, depending on the particle surface density. Each video was recorded at a different area of the SLB. Before each acquisition, the microscope stage was adjusted to the optimal focus position. Before the acquisition of each dataset, a protein standard was measured to calibrate the contrast to mass conversion using the same acquisition parameters.

*Image analysis*. Videos were analysed using a custom-written Python package modified from a previously published version^21^. In short, video processing is divided into three steps: image processing, particle detection and contrast fitting. For image processing, each frame of the 60-s video at 270 Hz was normalized to the total detected photoelectron count. To detect the local reflectivity changes originating from light scattered by the diffusing proteins on the SLBs, we subtracted the constant background of the underlying glass roughness by applying a moving median ratiometric imaging analysis approach^21,60^. We chose a 2.2-s time window for the moving median, suitable for the expected masses and diffusion coefficients of the tethered proteins. To suppress low-spatial-frequency intensity modulations, originating from rapid laser scanning of the imaged area, we convoluted each frame with a spatial median kernel of size 15 × 15 pixels and divided the ratiometric frame accordingly. The results of these image-processing operations are images similar to the representative frame in Fig. 2b. For particle detection, individual particles were detected above the intrinsic noise of the SLB by cross-correlating each frame with a 13 × 13-pixel kernel of the experimentally obtained point spread function (ePSF) of individual proteins. The ePSF was calculated by averaging individual PSFs of multiple glass binding events of a monodisperse protein solution. Template detection was applied using the match_template function from the scikit-image Python package. A cut-off value for template matching was set to 0.4. Only pixels whose value was higher than the cut-off values and that were identified as local maxima within a spatial window of 4 × 4 pixels were considered as detection events. For contrast fitting, each candidate pixel then serves as the centre of a region of interest (ROI) of size 11 × 11 pixels and the initial guess for the fitting procedure. The contrast of the detected particle was extracted by fitting the *x*,*y* coordinates of the centre of the experimentally normalized (to 1) interpolated ePSF. The *x*,*y* positions were found by minimizing the square difference between the defined ROI around the detected particle and the ePSF shifted to the *x*,*y* position. The minimized function is given by

1 $${R}^{2}=\mathop{\Sigma }\limits_{i,j}{(c(x,y)\times {\rm{ePSF}}{(x,y)}_{i,j}-{{\rm{ROI}}}_{i,j})}^{2},$$

in which *i*,*j* are the indices of the *ij*th pixel of the ROI and *c*(*x*,*y*) is a scaling factor of the normalized ePSF that minimizes the *R*^2^ value at a given *x*,*y* position. *c*_min_(*x*_min_,*y*_min_) is the reported measured contrast of the protein/complex. The best fitted contrast at each iteration is given by

2 $$c(x,y)=\frac{{\Sigma }_{i,j}{\rm{ePSF}}{(x,y)}_{i,j}\times {{\rm{ROI}}}_{i,j}}{{\Sigma }_{i,j}{\rm{ePSF}}{(x,y)}_{i,j}^{2}}.$$

*Generating a trajectory from consecutive localizations*. Individual successful and consecutive fitting events across adjacent frames were connected into a single molecular trajectory using the same code published and explained previously^21^ using the trackpy Python package.

*Segmenting trajectories using step detection*. To segment each molecular trajectory to its specifically sampled oligomeric states, separated by 120 kDa, for better mass resolution on the histogram level, characterization of the thermodynamic (Fig. 2) and for calculation of the average transitions kinetics, we implemented a step detection algorithm^68^. We combined this implementation with a step size threshold of 50 kDa, which is much lower than the known steps of approximately 120 kDa owing to trimer additions and is slightly higher than the noise introduced by the bilayer interface (about 40 kDa standard deviation at 270 Hz). Specifically, the extra mass threshold introduced was used to avoid detection of small mass changes that result from lateral movement of particles during frame acquisition, leading to different blurring of the PSF and therefore to small contrast variations. Given the intrinsic bilayer noise level of about 40 kDa at a frame rate of 270 Hz and our interest in resolving transitions between known measured masses at raw frame rate, we found this threshold to be suitable. This was confirmed by simulated data of a known transition rate (Extended Data Fig. 2). Only trajectories longer than 20 frames (74 ms) were considered for segmentation, for which shorter trajectories (<20 frames) were considered without segmentation. The minimum segment was restricted to three frames (11 ms).

*Extraction of oligomeric mass and diffusion coefficient*. For calculation of the diffusion coefficient, all detected trajectories and molecular segments were considered similarly. The diffusion coefficient was calculated as previously reported^21^, for trajectories longer than ten frames (37 ms). For shorter trajectories, we did not include a measure of the mobility. The molecular mass of each trajectory or segment was calculated by the median value of the mass trajectory. For a given diffusion coefficient, the assigned mass was corrected to take into account the motion blur that smears the detected and fitted PSF. This smearing effect lowers the fitted contrast by several percent, depending on the diffusion coefficient of the protein and the mass. The blur correction was validated both experimentally and with simulations for different masses, diffusion coefficients and acquisition parameters, as described previously^21,60^. The masses of molecular trajectories to which a diffusion coefficient was not assigned were not corrected.

*Plotting mass histograms and calculating surface molar fractions*. To calculate the surface densities of different oligomeric species, we generated weighted mass histograms from the trajectory dataset. To avoid noise detection at lower masses, we considered only trajectories longer than ten frames (37 ms); short segments of long trajectories were included even if their length was shorter than ten frames. The contribution of each mass trajectory or segment was weighted by its length and the final histogram was divided by the total number of frames per video and by the detected area. This results in a mass histogram in which the *y*-axis represents the average number of detected molecular species per detected area (or surface density). The histograms (Fig. 2) were then fitted to a series of five Gaussian functions for the five oligomeric species, from one mi3-trimer to the pentagonal ring. The surface density of each oligomer was multiplied by the number of its trimeric subunits and the total surface density of trimers was calculated by the sum of all oligomers. Following normalization, the molar fraction of mi3-trimers in each oligomeric state is given by

3 $${X}_{n}=\frac{n{\rho }_{n}}{{\sum }_{n}n{\rho }_{n}},$$

in which *X*_*n*_ is the molar fraction of mi3-trimers in an oligomer of size *n* trimers and *ρ*_*n*_ is the surface density of this oligomer.

*Measurements and detection of mass changes*. To quantify the dissociation rate constant of the dimer and trimer of mi3-trimers (${k}_{{\rm{off}}}^{{\rm{dimer}}},{k}_{{\rm{off}}}^{{\rm{trimer}}}$), we performed three dynamic MP experiments at trimer surface densities of 0.25, 0.34 and 0.72 μm^−2^. The experiments were performed as described above, by adding 2 nM of hist-tag mi3-subunits on top of the SLB. Following an equilibration time of 30 min and for each bilayer, we consecutively measured 30 different areas on the SLB, each area of dimensions roughly 6.3 × 9.9 μm^2^, for 1 min and at an effective frame rate of 270 Hz. The detected molecular trajectories were analysed and segmented as described above. Following segmentation, segments attributed to dimeric and trimeric oligomers were defined as all mass traces whose median mass falls within the experimental range given by the overall mass distribution of the corresponding oligomer. Dissociation events for dimers or trimers were defined as any mass change during the molecular trajectory in which the final mass is lower than the initial mass and that the absolute mass change is larger than 50 kDa. Theoretically, direct analysis of the resulting distribution of dwell times before dissociation will provide information on the dissociation constant. However, this analysis is prone to several statistical and experimental biases, including: early termination of trajectories owing to particles leaving the field of view, termination of molecular trajectories owing to identity switching (wrong trajectory linking results from close proximity of particles below the diffraction limit) and mass fluctuations resulting from close proximity of particles that do not interact. We therefore focus our analysis on the calculation of the average observed transition rate. Here <*r*_*ij*_> is the average transition rate from an oligomeric state *i* to any oligomeric state *j*, in which *m*_*j*_ < *m*_*i*_, and *m* is the measured mass. Taking the inverse of this rate, *τ*_*ij*_ = <*r*_*ij*_>^−1^, represents the average characteristic timescale for disassembly of oligomer, *i*, or the average dwell time before disassembly. Calculation of the average dissociation rate for the *i*th oligomer, <*r*_*i*_> follows

4 $$ < {r}_{i} > =\frac{{\sum }_{j < i}{N}_{{ij}}}{{\sum }_{k}{t}_{i,k}}=\frac{{N}_{{\rm{diss}}.}^{(i)}}{{T}_{{\rm{total}}}^{(i)}}$$

Here *N*_*ij*_ is the number of detected transitions from state *i* to state *j*, in which *m*_*j*_ < *m*_*i*_, and *t*_*i*,*k*_ is the total observation time of the *k*th segment of state *i*. Therefore, the average is given by the total number of disassembly events, ${N}_{{\rm{diss}}.}^{(i)}$, divided by the total observation time, ${T}_{{\rm{total}}}^{(i)}$. An example of the calculation for a representative trace is shown in Extended Data Fig. 3. The average dissociation rate was calculated for the dimeric and trimeric states for each 1-min dynamic MP video and converted to the average lifetime, *τ*_*i*_ = <*r*_*i*_>^−1^. A distribution of the 30 measured average lifetimes for the two oligomers is shown in Supplementary Fig. 5. The average lifetimes across different surface densities are shown in Fig. 2 (inset). Also, because for two-dimensional reactions the rate constant depends on the local distribution of proteins, the ratio of the average lifetimes of the dimer and trimer per video was calculated as well, as shown in Extended Data Fig. 2.

#### Dynamic MP measurements (Fig. 3)

*Data acquisition*. Dynamic MP measurements of solution bulk assembly kinetics from tethered pentamers (Fig. 3) were performed on the same commercial mass photometer (OneMP, Refeyn Ltd.). Here acquisition used a custom field of view of size 15.4 × 13.2 μm^2^ to allow maximum statistics and a frame rate of 250 Hz for maximum temporal resolution. Each measurement corresponds to acquiring a 60-s video. No further averaging was applied. Following the formation of the SLB and washing excess vesicles from the surface, 2 nM of tagged subunits were added to the gasket. The density of mi3-trimers was controlled by the incubation time. The solution of the tagged mi3-subunits was replaced with assembly buffer at least five times, washing away subunits in solution, and the system was allowed to equilibrate for 30 min to allow formation of pentamers on the surface. The initial mass distribution was measured and the assembly reaction was initiated by adding an equilibrated solution of the reassembled, untagged-mi3-VLPs at total protein concentrations of 88, 44 and 29 nM. The added solution of equilibrated VLPs does not contain the histidine tag modification and therefore particles do not bind the SLB (Supplementary Figs. 5 and 18) and bind surface-assembled pentamers instead. The assembly process of surface pentamers into fully assembled VLPs was monitored by acquiring consecutive 1-min videos, each at a different area of the SLB and for approximately 40 min per technical repeat. At each assembly condition, we repeated the experiment three times. For each repeat, a new SLB was formed and the above procedure was followed. Each repeat of assembly measurements represents a kinetic measurement of approximately 1,000 particles (about 20–30 particles per measurement multiplied by approximately 30–40 time points).

*Analysing trajectories to extract mass distributions*. Mass histograms of the dynamic MP experiments shown in Fig. 3 and Supplementary Figs. 10–17 were processed in the same way as described above, with only one extra step. The approximately four times larger field of view used here to increase statistics results in small optical contrast inhomogeneities across the imaged field of view. To quantitatively correct these small variations (several percent), we performed a standard MP experiment (similar to the procedure described above for a landing assay on a glass surface) using citrate synthase protein, a monodisperse protein calibrant with a known mass. Using this calibrant, we constructed a two-dimensional map of relative variations of the measured mass as a function of the *x*,*y* position across the imaged field of view (Supplementary Fig. 9). Following the fitting stage, we used this map to correct each measured particle contrast according to its fitted *x*,*y* position. Because the corrected mass is a constant function related to the microscope, the same correction was used for all of the measurements that correspond to the same size of the field of view. The experimentally calibrated relative contrast variation is shown in Supplementary Fig. 9.

#### Single-complex assembly experiments

*Coverslip preparation and photolithography*. Confined SLBs were prepared using a previously published protocol^69,70^, with modifications as detailed in ref. ^53^. Briefly, glass coverslips were cleaned as described above. Following plasma cleaning, the coverslips were rinsed with Milli-Q water, dried with nitrogen and fitted with silicone gaskets. The gaskets were filled with 50 µl of 2 µg ml^−1^ PLL(20)-g[3.5]-PEG(2) (SuSoS Surface Technologies) and incubated for 30 min at room temperature. After incubation, the coverslips were rinsed with Milli-Q water, dried with nitrogen and exposed to deep ultraviolet light using a mask aligner (Suss MJB4, HgXe 500 W source) for 60 min through a custom-made chrome photolithography mask containing an array of 5-µm-diameter circles. Finally, the coverslips were rinsed with Milli-Q water, dried with nitrogen and stored at −20 °C for up to three months before use.

*Data acquisition*. Patterned supported SLBs were prepared using the photolithographically patterned glass coverslips and the SLB preparation procedure. Formation of surface pentamers confined to the SLB followed a similar protocol as described above. For assembly from pentamers to full VLPs, an equilibrated solution of preassembled untagged-mi3-VLPs at total mi3-monomer concentrations of 88 or 100 nM was added on top of the confined pentamers. The focus position was then found and the confined SLBs were measured for 5 min from the time of solution addition using a OneMP with a field of view of size 15.4 × 13.2 μm^2^.

*Data analysis*. We carried out data analysis in the same manner as for standard dynamic MP analysis (see above), with an extra step for correcting long tracking of single assembled VLPs in the case in which more than one pentagonal ring complex was confined in the same trap. After automatic trajectory linking, resulting trajectories were further manually examined and linked using frame, position and contrast values. In several cases, slower mobility of particles close to the edge of the trap affected the ratiometric contrast, owing to the median background subtraction, and we manually found periods in which the VLPs did not move and reanalysed these with a modified version of ratiometric analysis in which the background is estimated using interpolation of the raw images from 250 frames before immobilization to 250 frames after immobilization. For longer immobilization periods, in which the modified ratiometric analysis could not overcome sample drift, we did not consider the mass measurements from the corresponding frames (Supplementary Fig. 21). The contrast values of the initial pentameric rings were converted to mass by aligning the initial contrast values (first 10 s) to the expected pentamer mass, following calibration of the mass to contrast conversion for the same acquisition settings.

*Mass trajectories analysis*. We performed dwell times analysis using the same step detection procedure described above to identify transitions between molecular states, with a threshold of 70 kDa. The resulting trajectories are shown in Supplementary Figs. 19 and 20. The molecular states were defined according to their expected masses (Supplementary Fig. 19, projected histograms) with a possible error of up to 5% owing to variations in the contrast to mass conversion as a result of variation in focus position. The trajectories and molecular transitions were also examined manually to validate the transition times between stable intermediates. In cases where a molecular state was not detected in a particular mass trace, its dwell time was set to 0.

## Online content

Any methods, additional references, Nature Portfolio reporting summaries, source data, extended data, supplementary information, acknowledgements, peer review information; details of author contributions and competing interests; and statements of data and code availability are available at https://doi.org/10.1038/s41586-026-10948-z.

## Supplementary information

Supplementary Information This file contains Supplementary Notes 1–7, Supplementary Figs. 1–22 and Supplementary References

Peer Review File

Supplementary Video 1 **Two-dimensional self-assembly dynamics of mi3-VLP subunits on an SLB**. The left panel shows 5 s of a representative median-subtracted video from a MP measurement of mi3-subunits tethered to an SLB undergoing self-assembly. The video was acquired at 270 Hz. The middle panel shows the raw mass-versus-time trace, with horizontal lines indicating the expected masses of the different oligomeric states. The right panel shows the oligomeric structures that correspond to the measured masses

Supplementary Video 2 **Assembly of a pentameric ring into a complete mi3-VLP monitored by continuous MP on a patterned SLB**. The left panel shows 250 s of a representative median-subtracted video from an MP measurement of a pentameric ring tethered to an SLB. On addition of mi3-subunits to the solution, the pentamer assembles into a complete VLP. The middle panel shows the corresponding mass-versus-time trace, with grey dots indicating raw data at the native frame rate (250 Hz) and a black line representing a running median over 100 frames (400 ms). Horizontal lines mark the expected oligomeric masses of assembly intermediates, with red lines highlighting the masses of topologically closed states. The right panel illustrates the structures of these topologically closed intermediates along the assembly pathway

## Data Availability

The raw data and code required to reproduce all of the manuscript figures can be found in the University of Oxford Research Archive (https://doi.org/10.5287/ora-nb59kaxzj).
