# Supplementary material for: Molecular-level observation of the self-assembly of a virus-like particle
Source: Nature. 2026 Sep 16;657(8132):653–60. doi: 10.1038/s41586-026-10948-z (PMC13581590; doi:10.1038/s41586-026-10948-z)
Supplement: Supplementary file 1 [file 41586_2026_10948_MOESM1_ESM.pdf]

nature portfolio

https://doi.org/10.1038/s41586-026-10948-z

# Supplementary information

# Molecular-level observation of the self-assembly of a virus-like particle

In the format provided by the authors and unedited

Supplementary notes for:

# Molecular-level observation of the self-assembly of a virus-like particle

Roi Asor[1,2,*,†], Dan Loewenthal[1,2,†], Diana Melnyk[3], Tiong Kit Tan[3,4] and Philipp Kukura[1,2,*]

[1]Department of Chemistry, University of Oxford, Physical and Theoretical Chemistry Laboratory, OX1 3QZ, United Kingdom
[2]Kavli Institute for Nanoscience Discovery, Biochemistry Building, OX1 3QU, United Kingdom
[3]MRC Translational Immune Discovery Unit, Weatherall Institute of Molecular Medicine, University of Oxford, OX3 9DS Oxford, United Kingdom
[4]Chinese Academy of Medical Sciences Oxford Institute, Nuffield Department of Medicine, University of Oxford, OX3 7BN Oxford, United Kingdom

† Equal contribution *To whom correspondence should be addressed: roi.asor@chem.ox.ac.uk philipp.kukura@chem.ox.ac.uk

## Table of Contents

**Supplementary information:** ........ **2**
**Materials** ........ **2**
Buffers lipids and commercial proteins ........ 2
**Supplementary note 1: Difference in measured mass of the complete VLP** ........ **2**
**Supplementary note 2: Solution thermodynamic model (Figure 1d)** ........ **3**
**Supplementary note 3: Characterising two-dimensional interactions** ........ **3**
3.1 Thermodynamic characterisation of the 2D thermodynamic model in Figure 2 ........ 3
3.2 Quantifying the effect of dimensionality reduction ........ 4
3.3 Quantifying $k_{off}$ and $k_{on}$ and estimation of the theoretical 2D diffusion limited rate constant ........ 4
**Supplementary note 4: Dynamic MP simulations for testing the extracted lifetimes** ........ **5**
4.1 Simulation procedure ........ 5
4.2 Extracting lifetimes ........ 6
**Supplementary note 5: Calculation of the grand canonical free energy potential** ........ **6**
**Supplementary note 6: Dynamic MP measurements (Figure 4 and Extended Data Fig. 4): Bulk kinetics modelling** ........ **7**
**Supplementary note 7: Stochastic kinetic simulations (Extended Data Fig. 8)** ........ **8**
**Supplementary Figures S1-S22** ........ **9**
**Supplementary references** ........ **31**

# Supplementary information:

## Materials

***Buffers lipids and commercial proteins***

HEPES (H3375), Chloroform (288306), Magnesium Chloride hexahydrate (M2670), Sodium Chloride (S3014) and Trizma base (T1503) were purchased from Merck Life Science UK Limited. Dulbecco's Phosphate Buffered Saline (DPBS) was purchased from ThermoFisher Scientific. UltraPure 10% SDS (15553-035, Invitrogen). Lipids, 1-palmitoyl-2-oleoyl-glycero-3-phosphocholine, 850457 (POPC); 1,2-dioleoyl-sn-glycero-3-[(N-(5-amino-1-carboxypentyl)iminodiacetic acid)succinyl] (nickel salt), 790404P (DGS-NTA) and 1,2-dioleoyl-sn-glycero-3-phosphoethanolamine-N-[methoxy(polyethyleneglycol)-550] (ammonium salt), 880530 (18:1 PEG550 PE) were purchased from Avanti Polar Lipids. The lipid powders were kept at -80 °C and aliquots were stored in chloroform at −20 °C. SpyTag-polyHistidine peptide was purchased from BioMatik, sequence: RGVPHIVMVDAYKRYKGSGESGSGHHHHHH.

## Supplementary note 1: Difference in measured mass of the complete VLP

When examining the measured mass of soluble mi3-trimers the average measured mass is $108 \pm 1\ k$Da (**Fig. S1a**). Therefore, the expected measured mass of the mi3-VLP is expected to be $2150 \pm 24\ kDa$. However, the particles are measured at $2030 \pm 10k$Da (**Fig. S1b,** top), slightly below the expected mass. The origin of this small change in the measured interferometric contrast stems from the dimensions of the VLP particles, extending up to about 20 nm away from the glass-solution interface (**Fig. S1c**), while the contrast to mass conversion was calibrated using small proteins that bind close to this interface. This height change leads to differences in the measured interferometric optical contrast owing to the added optical path difference between the reference light, reflected from the glass-solution interface and the scattered light from the VLP. The measured contrast is therefore a function of the *z* coordinates through,

$$C \propto 2\frac{|E_{scat}|}{|E_{ref}|}\cos\left(\phi_0 + \Delta OP(z)\right).$$

Where, $|E_{scat}|$ and $|E_{ref}|$ are the real amplitudes of the scattered and reflected fields and $\phi_0$ is an intrinsic phase that includes the Gouy phase shift between the reference field and the field emitted by a point scatterer, as well as any additional phase differences from components of the imaging path such as the partial reflector at the back focal plane and defocusing. Assuming that the microscope was optimised to provide the maximum contrast at the glass-solution interface, we can take $\phi_0(z = 0) \approx 0$. $\Delta OP(z)$, is the additional optical path that the scattered field accumulate because of a scatterer that is at distance z above the interface. The optical path difference is given by $\Delta OP = \frac{4\pi \cdot \delta z \cdot n_m}{\lambda}$, and therefore the contribution to the overall contrast as a function of the protein position will be,

$$c_0 \cdot \cos \Delta OP(z) = c_0 \cdot \cos\frac{4\pi \cdot \delta z \cdot n_m}{\lambda}.$$

To estimate the contrast, we calculated the sum $c_{VLP} = c_0 \sum_i \cos\frac{4\pi \cdot \delta z_i \cdot n_m}{\lambda}$, where $\delta_{z_i}$, $n_m$, $\lambda$ are the *z* coordinate of the *i*-th trimeric subunit, the solution refractive index and the wavelength of the laser light source, respectively. Calculation of the expected measured mass, using the above equation as a function of the number of subunits (while increasing the *z* distances of the trimeric subunits), is shown in **Fig. 1c**. Based on this simple calculation while taking into account the measured mass of the mi3 trimer, the expected measured mass of the VLP should be $2015 \pm 24\ k$Da, which agrees well with the measured value. This difference between the expected mass and the measured value suggests that the intrinsic phase shift of the setup, $\phi_0 < -0.01\pi$, which indeed close to zero.

## Supplementary note 2: Solution thermodynamic model (Figure 1d)

The thermodynamic model for interacting subunits considers two interaction parameters, the monomer-monomer interaction that forms the trimeric subunit, and the trimer-trimer interaction that forms the dodecahedral VLP symmetry. These two interactions are taking into account by the standard free energy changes $\Delta G_1^\circ, \Delta G_2^\circ$, respectively. The law of mass action predicts that the distribution of oligomers will follow,

$$C_n \nu_0 = \sum_i (C_1 \nu_0)^n g_{n,i} \cdot s_{n,i} \cdot \exp\left(-\frac{\Delta G_{n,i}^\circ}{k_B T}\right) \quad (1)$$

Where, $g_{n,i}$ is the number of unique different topological configurations that are composed of $n$ monomeric subunits and includes $c_i$ inter-trimer contacts, $s_{n,i}$ is a kinetic degeneracy factor[1], $\Delta G_{n,i} = 3n \cdot \Delta G_1^\circ + c_i \Delta G_2^\circ$, and $\nu_0$ is the standard state, taken as 1 $M^{-1}$ in our calculation (therefore $C_n$ represent the concentration in M), and $\sum_n nC_n = C_{Total}$, where $C_{Total}$ is the total concentration of mi3-monomers in solution.

The VLP is a regular dodecahedron composed of 20 trimeric subunits, the variation of $g_{n,i}$ with the configuration index, $i$, is of the order of between 1-100. Therefore, $\ln g_{n_i} \ll \Delta G_{n,1/2}^\circ$ allowing us to neglect the contribution of this term. This means that the distribution of states can be estimated with high accuracy by considering the most stable state for each oligomeric size and taking only one of its configurations into consideration. This leads to the simplified expression,

$$C_n = (C_1 \nu_0)^n \cdot s_n \cdot \exp\left(-\frac{\Delta G_n^\circ}{k_B T}\right) \quad (2)$$

The law of mass action predicts that at equilibrium and for N>>1, almost all subunits will be found as either free mi3-subunits or complete VLPs. In our case, this prediction is validated experimentally by the measured mass histograms (**Fig. S3**). We therefore include three possible states in the thermodynamic model, corresponding to the mi3-monomers, mi3-trimers and complete VLPs. We can now write the conservation of mass as,

$$C_1^{Total} \nu_0 = \mathrm{C}_1 \nu_0 + (C_1 \nu_0)^3 \cdot e^{-\frac{3\Delta G_1^\circ + \ln S_{Trimer}}{k_\mathrm{B} T}} + (C_1 \nu_0)^{60} \cdot e^{-\frac{20\cdot(3\cdot\Delta G_1{}^\circ + \ln S_{Trimer}) + 30\cdot(\Delta G_2^\circ + \ln S_{VLP})}{k_\mathrm{B} T}} \quad (3)$$

where, the degeneracy factors for the trimer and the complete VLPs are $S_{Trimer} = \frac{2^3}{3}$ and $S_{VLP} = \frac{3^{19}}{20}$. Conservation of mass allows us to calculate the concentrations of the three molecular species for each combination of $C_1^{Total}$ and the two interaction free energies.

To extract the standard free energy changes, $\Delta G_{1/2}^\circ$, we fitted a titration series at different total protein concentrations (**Fig. 1**). At each iteration, the model assumed the value of the two standard free energies and then solved for the relative molar fractions of the different species according to the conservation law, until good agreement between the model and the data has been reached.

## Supplementary note 3: Characterising two-dimensional interactions

### *3.1 Thermodynamic characterisation of the 2D thermodynamic model in Figure 2*

A two-dimensional thermodynamic model was used to characterise the distribution of oligomeric states on the supported lipid bilayer (SLB). Here, we assume that the inter-trimer pairwise interaction (**Fig. S6, top**) is identical for all contacts formed, and that there is no cooperativity between binding sites. This means that the model includes only one parameter, $\Delta G^\circ(2D)$, the free energy change in forming an inter-trimer contact. The model therefore includes the pairwise interaction strength and incorporates the kinetic degeneracy for each transition (**Fig. S6**, bottom). We also emphasise that our ability to track individual trimers and oligomers on the surface allows us to directly measure the number density of subunits instead of assuming it, therefore the total density is a measurable rather than an adjustable parameter. In a similar way to solution (**Supplementary note 1**), the law of mass action is given by,

$$\rho_{Total} a_0 = \Sigma_{\mathrm{n}=1}^{5} (\rho_1 a_0)^n \cdot s_n \cdot \exp\left(-\frac{\Delta G_n^\circ(2D)}{k_B T}\right) \quad (4)$$

where, $\rho_n$ is the number density of trimeric subunits, $a_0$ is the standard state taken as the cross section of the trimers, $s_n$ is the molecular degeneracy resulting from the multivalency, and $\Delta G_n = (n-1)\cdot \Delta G^{\circ}(2D)$ for $n \in \{2,3,4\}$ and $\Delta G_5 = 5\Delta G^{\circ}(2D)$. The titration presented in **Fig. 2d** was fitted to the above equation, to extract the interaction parameter, $\Delta G^{\circ}(2D)$.

### *3.2 Quantifying the effect of dimensionality reduction*

To quantify to which degree dimensionality reduction enhances the effective interactions, we consider the phenomenological length scale, useful to characterise this enhancement effect, $h = \frac{K_D^{2D}}{K_D^{3D}}$, where $K_D^{2D}$ and $K_D^{3D}$ represent the dissociation constant in 2D (surface) and 3D (solution). The size of this length scale determines whether oligomers form more favourably in solution (3D) or on the SLB. Given that the standard free energy changes of interactions are written in the scale of molecular dimensions ($\nu_\circ^{-1}$ for 3D and $a_\circ^{-1}$ for 2D, where $\nu_\circ$ and $a_\circ$ are the specific volume and cross section of the protein) this length scale can be written as $h = \frac{\nu_\circ}{a_\circ} e^{-\frac{\Delta G^{\circ}(2D)-\Delta G^{\circ}(3D)}{k_B T}}$. Therefore, enhancement is expected when $h < \frac{\nu_\circ}{a_\circ} = \frac{4}{3} r$, where the equality assumes a globular protein. Reported estimations of $h$ for preferred dimerization on membranes is on the nanometer scale, these however vary between different proteins and experimental or theoretical methods[2-5]. Here, we have the opportunity to directly compare the interactions in 3D and 2D on a single molecule level and provide a quantification of $h$. Given our quantification of the interactions in solution and on the membrane we get $h = \frac{0.74 \pm 0.15\ \mu m^{-2}}{2.5 \pm 0.4\ \mu M} = 0.5 \pm 0.1\ nm < \frac{4}{3} r_h = 4.4\ nm$, where $r_h$ is the hydrodynamic radius of the trimer calculated using HullRad v10 software[6].

### *3.3 Quantifying $k_{off}$ and $k_{on}$ and estimation of the theoretical 2D diffusion limited rate constant*

The resulting transition rates are 0.65 ± 0.05 s for the dissociation of dimers of trimers and 0.36 ± 0.04 s for dissociation of trimers of trimers (**Fig 2f** and **Extended Data Fig. 1**), yielding $k_{off}$ = $1.5 \pm 0.1\ s^{-1}$ and $2.8 \pm 0.3\ s^{-1}$, respectively. Combining with the 2D dissociation constant we can calculate the bimolecular $k_{on}$ for dimerisation by, $k_{on} = \frac{k_{off}}{K_D} = 2.15 \pm 0.45 \frac{\mu m^2}{s}$ and the same calculation for the trimer formation yields $2.55 \pm 0.49 \frac{\mu m^2}{s}$. To place these values in context, we estimate the 2D diffusion-limited rate constant $k_{on}^{2D}(t)$ using our experimental quantification of the 2D diffusion coefficient per particle, which is inversely proportional to the oligomeric state (**Extended Data Fig. 1 and S8**).

To estimate the theoretical limit of the time-dependent two-dimensional forward rate constant, $k^{2D}(t)$, we used the previously derived equation[2],

$$k(t) = 4\pi D \left( \frac{1}{\log\left(\frac{4Dt}{\sigma^2}\right) - 2\gamma} - \frac{\gamma}{\left(\log\left(\frac{4Dt}{\sigma^2}\right) - 2\gamma\right)^2} \right) \quad (5)$$

where $\sigma$ and $D$ are the encounter radius and 2D (experimentally measured **Fig. S6**) diffusion coefficient, and $\gamma$ is the Euler's constant. This equation approximates the rate constant at the long-time limit, where $t \gg \frac{\sigma^2}{D}$. Focusing on dimerisation, the subunits have an average diffusion coefficient of 1.25 $\mu m^2\ s^{-1}$ (**Extended Data Fig. 1**), and in our case $\sigma = 0.0075\ \mu m$ (estimated from the atomic structure of the mi3-trimer[6]). Therefore, $D = 2.5\ \mu m^2\ s^{-1}$, which gives $t \gg 45\ \mu s$. Evaluated between 0.3 and 0.7 s, which is the timescale relevant to the observed dissociation, yields $k_{on}^{2D}(t) \approx 3\ \mu m^2 s^{-1}$ (**Extended Data Fig. 1**), in good agreement with our experimentally determined value.

## Supplementary note 4: Dynamic MP simulations for testing the extracted lifetimes

To validate the procedure of extracting the average lifetimes, we constructed a simulation framework to generate an approximation for the experimental dynamic MP movies, including known transitions between molecular species.

### *4.1 Simulation procedure*

*Trajectory generation:* To generate trajectories for a simulation of an experimental 7x10 $\mu m^2$ FOV, a larger FOV of 21x17 $\mu m^2$ with periodic boundary conditions was generated. This way we could keep a constant averaged surface density for individual species, while allowing the number of particles in the field of view to fluctuate. For a given total mi3-trimer surface density, an equilibrium distribution of oligomers was generated based on the thermodynamic model described by **equation S7.** From this distribution, *N* random samples of oligomers were taken, where $N/A$ gives the required surface density, with *A*, the area of the FOV. For each oligomer, a 2D diffusion trajectory of length 2 min and temporal resolution of 5.4 kHz (x10 of the maximum frame rate) was generated according to,

$$x_{t_i} = x_{t_{i-1}} + \sqrt{2D\Delta t} \times randn(0,1) \quad (6)$$

$$y_{t_i} = y_{t_{i-1}} + \sqrt{2D\Delta t} \times randn(0,1)$$

Here, $x_{t_i}$ and $y_{t_i}$are the instantaneous position of the particle, $D$ is the diffusion coefficient, $\Delta t$ was set to 0.185 ms (for 5.4 kHz), and randn(0,1) is a randomly drawn number from a standard normal distribution with mean 0 and variance of 1. The initial positions ($x_{t=0,i}, y_{t=0,i}$) were chosen randomly withing the FOV. The diffusion coefficient and the mass of the particles were set according to the oligomer identity and the experimentally measured values. This way we could simulate the molecular transitions at similar conditions as the experimental data. To simulate the lifetime of the dimeric oligomer (dimer of mi3-trimers), all randomly selected particles that belong to either the mi3-trimers or dimer of mi3-trimers were considered together, and the dynamic transitions between mi3-trimers and dimer of trimers were simulated by a given $k_{off}$ that corresponds to the simulated lifetime, and $k_{on} = \frac{k_{off}}{K_D}$, where $K_D$ is the measured dissociation constant for the dimer of trimers (**Fig. 2**). This way, we could simulate the dynamic transitions between the two molecular species while maintaining the overall equilibrated distribution.

For the trajectories classified as either mi3-trimer or dimer of mi3-trimers, a transition involved a change of mass from dimer of trimers to mi3-trimer was randomly sampled according to an exponential distribution,

$$\delta t_{dissociation} = \frac{1}{k_{off}} \log(1/r) \quad (7)$$

Where, $\delta t_{dissociation}$ is the time difference since the particle first assigned the mass of a dimer of trimers and the transition time, r is a random number drawn from a uniform distribution in the range 0 and 1.

In the same way, stochastic mass transition from a trimer to a dimer of trimers was sampled as,

$$\delta t_{association} = \frac{1}{k_{on}} \log(1/r). \quad (8)$$

At the centre of the large 21x17 $\mu m^2$ FOV, we defined a smaller FOV that is 10% larger than the experimental FOV. Only segments of the trajectories that were occupying this area during the 1 or 2 min of the simulations were saved for further steps.

*Movie generation:* To generate a complete simulated dynamic-MP movie, a 2 min experimental movie of an SLB without added proteins was recorded at 540 Hz and under identical FOV as the data. This

movie was used as the background for generating the simulated movies. For every frame, the coordinates of each particle $(x_{ti}, y_{ti})$ were convolved with an experimentally obtained kernel of the experimental point spread function (ePSF). The kernel size was 25x25 pixels, which is equivalent for positioning a particle centered on this position. This way, we generated a noise free movie of the simulated diffusing ePSFs. Since the simulation time resolution is 10 times higher than the acquired movie (to simulate motion-induced PSF blurring during acquisition), the amplitude of the convoluted ePSF was set to $\frac{c_i}{10}$, where $c_i$ is the assigned contrast of the PSF (negative) corresponding to the oligomeric state of the simulated particle. The generated array of diffusing ePSFs was then binned 10-fold (summing batches of 10 frames together) to down-sample the simulated movie 10 times, back to the maximal experimental frame rate of 540 Hz and further binned 2-fold to fit the experimental conditions used. The central area of the simulated movie, similar to the experimental movie, was cut, to fit the size of the experimental FOV, and together with the experimental background movie we generated a simulated movie with the experimental noise components according to,

$$Sim(t, x, y) = BG(t, x, y) + \overline{BG(t, x, y)}_{N=500} \times ePSF(t, x, y)$$

where, $BG(t, x, y)$ is the measured movie of the washed SLB, $\overline{BG(t, x, y)}_{N=500}$ is the same movie following a rolling time average of 500 frames, to suppress background noise and $ePSF(t, x, y)$ is the noise free simulated movie of diffusing ePSFs.

***4.2 Extracting lifetimes***

We simulated a series of dynamic MP movies, where the lifetimes of the dimers were set to values between 0.3 and 3.5 seconds, with 5 repeats for each condition. Each movie was analysed with the same analysis code as the experimental movies. The extracted lifetimes were compared to the simulated values, validating the analysis method (**Extended Data Fig. 2**).

## Supplementary note 5: Calculation of the grand canonical free energy potential

Computation of the solution grand canonical free energy potential (**Fig. 3** and **Extended Data Fig. 3**) follows previous derivations[1,7-9] and continues the same assumptions introduced in **Eq. S1**. The driving force for the assembly reaction is given by the change in chemical potential between any oligomer of size *n***,** and mi3 -trimeric building blocks. The molar fraction of the *n*-th oligomer is therefore given by,

$$\frac{\chi_n}{n} = e^{\frac{-n\cdot(\Delta\tilde{\mu}_n{}^{\circ} - \Delta\mu_1)}{k_B T}} \tag{S9}$$

Here, $\Delta\tilde{\mu}_n{}^{\circ}$ is the averaged standard chemical potential change of a trimeric mi3-subunit in an aggregate of size *n*, and $\Delta\mu_1$ is the chemical potential change of soluble mi3-trimer. Since we consider an equilibrated solution added above a passivating bilayer, the concentration of the trimeric mi3 subunits remains constant. Therefore, the chemical potential of any oligomer on the surface of the bilayer is calculated relative to $\Delta\mu_1 = k_B T \ln \chi_1 \approx Const.$ Assuming a similar set of intermediates to **Eq. S1**, where only the most stable structure for each oligomeric size is considered, this difference can be written as,

$$\Delta\tilde{\mu}_n{}^{\circ} - \Delta\mu_1 = \frac{-c_n\cdot\epsilon - k_B T\cdot ln s_n}{n} - k_B T ln\chi_1 \tag{S10}$$

Here, $\epsilon$ is the pairwise interaction free energy gain in units of $k_B T$, $c_n$ is the number of inter-trimers contacts, and $s_n$ is a degeneracy factor[1] similar to that in **Eq. S1**. At the pseudo-critical concentration of $\sim 17\ nM$ of trimers, this difference is shown in **Extended Data Fig. 3b**. $\Delta\tilde{\mu}_n{}^{\circ}$ is a decaying function of *n*, and the difference in chemical potential with respect to the soluble trimer is always $\geq 0$, as expected at equilibrium where no net driving force for assembly exist. The change in the average coordination number of the incoming trimers as a function of *n* is indicated by the local minima of the curve. This change becomes more apparent when comparing **Eq, S14** without the degeneracy factor

(**Extended Data Fig. 3b** orange curve) to a linear oligomeric growth model (green curve), where addition of each subunit introduces one inter-subunit bond. These local minima, correspond to the topologically closed, stable intermediates.

The relative occurrence of oligomers is better illustrated by examining the grand canonical potential under a fixed chemical potential of subunits (**Extended Data Fig. 3c**),

$$\Theta_{\mathrm{n}}(\epsilon, \Delta\mu_1) = n\Delta\tilde{\mu}_n{}^\circ - n\Delta\mu_1 = -c_n \cdot \epsilon - k_B T \cdot ln s_n - n \cdot ln\chi_1 \,. \qquad \text{(S11)}$$

In solution the chemical potential of 20 trimeric subunits is equal to the chemical potential of the trimers in a complete VLP (**Extended Data Fig. 3c**, top), and $\Theta_{\mathrm{n}}(\epsilon, \Delta\mu_1)$ represents the potential landscape at chemical equilibrium.

When the solution is brought into contact with the surface pentamers, the grand canonical potential remains unchanged, however, the surface now contains pentamers in excess relative to their equilibrium fraction in solution (disassembly of pentamers is irrelevant as the surface distribution itself is at equilibrium). The relevant chemical driving force is therefore between the pentameric rings on the surface and the complete VLPs, mediated by the constant chemical potential of the trimeric subunits in solution,

$$\Delta\Theta_{5\to 20}(\epsilon, \Delta\mu_1) = 5\Delta\tilde{\mu}_5{}^\circ - 20\Delta\tilde{\mu}_{20}{}^\circ + 15\Delta\mu_1 = -25 \cdot \epsilon - k_B T \cdot ln\frac{s_5}{s_{20}} + 15 \cdot ln\chi_1 \,. \qquad \text{(S12)}$$

Using the experimental value of the free energy per contact (10.7 $k_B T$, **Fig. 1**) and the concentration of trimeric subunits (17, 11.8, 7.4 nM) for the three experimental conditions yields driving forces of approximately $15, 10, 3$ $k_B T$, respectively for each concentration (orange marks in **Extended Data Fig. 3c**).

## Supplementary note 6: Dynamic MP measurements (Figure 4 and Extended Data Fig. 4): Bulk kinetics modelling

Aiming at connecting our single complex dynamics data (**Fig. 4**) with bulk distribution kinetics (**Fig. 3**), we divided the bulk mass histograms into three molecular states: pentamers, complete/close to complete VLP (> 1750 kDa) and intermediates that are between the pentamers and the VLPs in mass (**Extended Data Fig. 4**). Plotting the fraction of particles in each of these states as a function of time at different solution concentrations of mi3-subunits, reveals similar kinetics, however with different timescales (**Extended Data Fig. 4a**). Here, as expected, following the addition of free trimeric subunits to the solution, the molar fraction of pentamers decreases over time, which correlates to an increase in the fraction of the intermediates. The molar fraction of VLPs shows a sigmoidal curve, where its increase is connected to the decay of the intermediate fraction. This time variation fits a characteristic curve of consecutive reactions. Informed by our single complex dynamics experiments, showing low probability of disassembly back to the initial pentameric state, we included only forward reactions in modelling the kinetic rates. Fitting this simple model that takes into account the two reactions rates ($r_1$ and $r2$), results in good agreement with the data (**Extended Data Fig. 3a**). We attribute the small deviations between the model and the data of the pentamers mass fraction at the two lowest concentrations to the fact that the concentration of subunits in the solution above the SLB, although assumed constant, is slightly decaying over a period of a few tens of minutes at such low concentrations. Since the perturbation out of equilibrium is small to begin with and the concentrations in solution are very low (17-5.6 nM), small changes in the solution concentrations can result in slowing down or even stopping the conversion from pentamers to intermediates. To quantify the concentration dependence, we plotted $r_1$ and $r_2$ across the three conditions on a logarithmic scale (**Extended Data Fig. 3c**). Linear fits result with slopes of $2.4 \pm 0.1$ and $2.1 \pm 0.4$ for $r_1$ and $r_2$, respectively. From our single complex dynamics data, we know that the transition between the pentamer to the first stable intermediate state requires addition of three subunits (8-mer), whereas each subsequent face-closing step among

intermediates typically requires two subunits. Accordingly, the rates are expected to scale as $r \propto k[Trimer]^n$, where $n \approx 3$ for $r_1$ and $n \approx 2$, which fits very well with our results.

## Supplementary note 7: Stochastic kinetic simulations (Extended Data Fig. 8)

VLP assembly dynamics (**Extended Data Fig. 8**) were simulated using a simple Gillespie type stochastic kinetic Monte Carlo approach[10]. To model the time evolution of subunit association and dissociation to a growing VLP, we considered the VLP structure as an undirected graph comprised of 20 nodes, where each node has a connectivity of 3, corresponding to the number of interaction sites of the subunits. Therefore, each node represents an mi3- trimeric subunit and each edge represents a trimer-trimer interaction. Each simulated trajectory represents the assembly of one VLP particle, under a fixed concentration of trimeric subunits in solution.

Starting with a graph representing the pentagonal ring, in each iteration, all possible potential node additions or removals were enumerated to the current state of the graph. Nodes can be added through the creation of a new edge to the current state of the graph, while removal is only possible by deletion of a single edge that results in an increase in the connected components of the graph. In this case, the connection component that does not contain the original pentameric ring is removed. Physically, the later assumption states that subunits that are connected to the growing VLP by more than one interaction, cannot be removed, and therefore topologically closed structures cannot dissociate. The total propensity $a_\circ$ is calculated as the sum of all forward (association) and backward (dissociation) reaction propensities, given by:

$$a_\circ = \Sigma_i^{N_{ass}} a_i^{ass} + \Sigma_i a_i^{diss} \tag{13}$$

where,

$$a_i^{ass} = k_{i,on}[T], \;\; a_i^{diss} = k_{i,off}. \tag{14}$$

here, $k_{on}$ and $k_{off}$ are the microscopic association and dissociating rate constants per site, and $[T]$ is the molar concentration of mi3-trimers in solution. Random numbers $r_1, r_2 \in (0,1)$ are sampled from a uniform distribution to determine the next reaction time increment:

$$\tau = \frac{1}{a_\circ} \ln \frac{1}{r_1} \tag{15}$$

and to select the specific event that occurs by the smallest index that satisfy,

$$\Sigma_j a_j > r_2 a_\circ \tag{16}$$

where, $a_j$ is a vector containing the combined propensities for association and dissociation events. According to the chosen event we updated the graph and the recorded time increment. In each trajectory, the simulation proceeds until either the maximum simulation time of 1 h or VLP completion. Simulations were performed at three concentrations 17, 9, and 5.6 nM of trimeric subunits. The association rate and dissociation constant were set to $10^7\, M^{-1}s^{-1}$ and $1\, \mu M$ for considering weak micromolar interactions that are diffusion limited.

## Supplementary Figures S1-S22

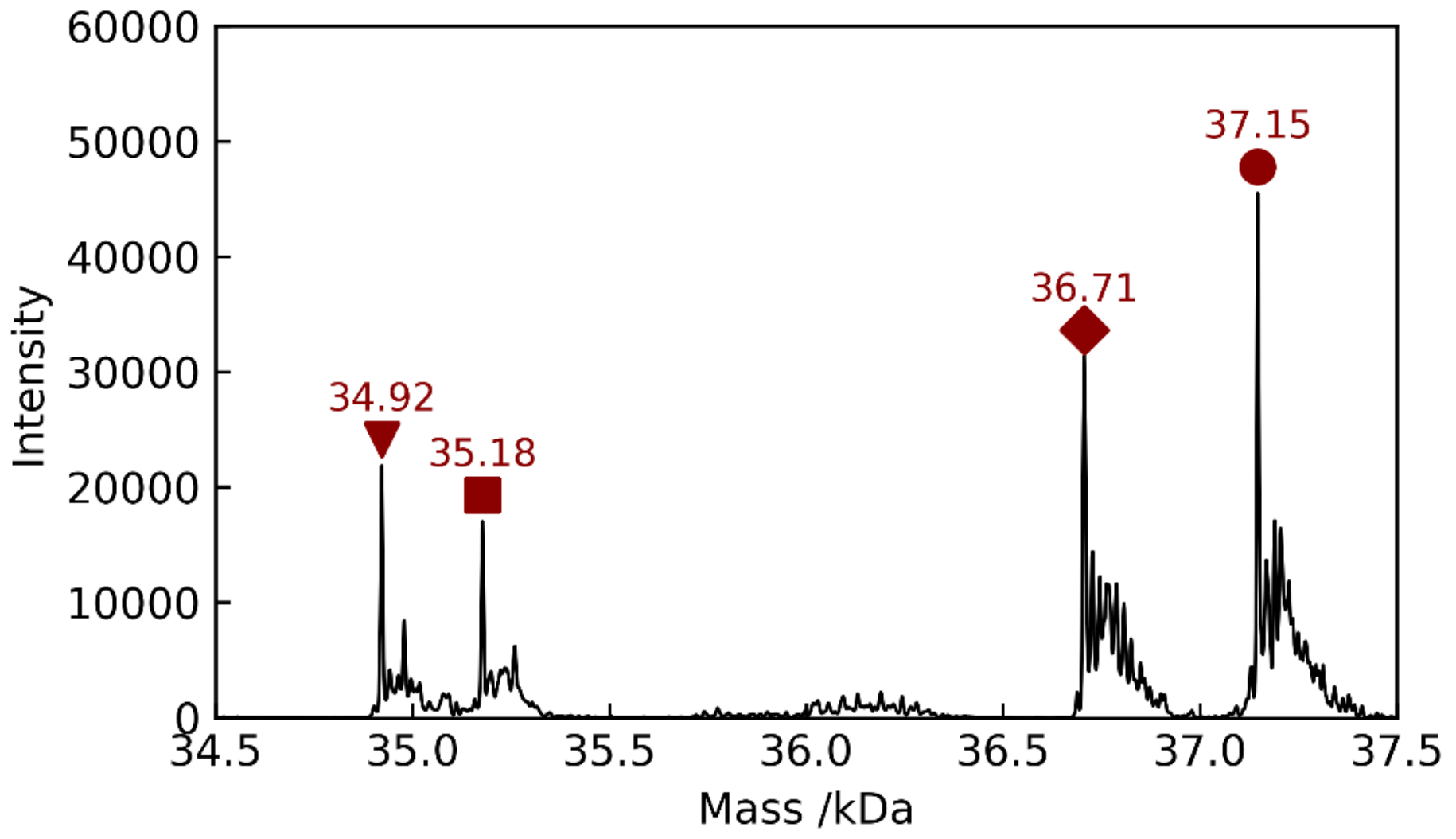


**Figure S1: Mass spectrometry of the isolated, assembled SC003-mi3 construct reveals two discrete species.** The higher-mass dominant forms are consistent with the full-length SC003-mi3 subunit. The smaller population separated by ~2 kDa could result from C-terminal truncation. Mass spectrometric analysis was carried out on an Agilent 1290 LC system coupled to an Agilent 6530 QTOF mass spectrometer (Agilent Technologies) using a reverse-phase setup. Samples (20 µL of a 1 µg/mL solution) were injected onto a 2.1 × 150 mm Zorbax 300SB-C3 column (5 µm particle size) maintained at ambient temperature. Mobile phases consisted of chromatography-grade water (A) and acetonitrile (B) (Optima LC/MS grade, Fisher Chemical), each supplemented with 0.1% formic acid (Optima LC/MS grade, Fisher Chemical). The chromatographic gradient was programmed as follows (time in minutes/A/B): 0.00/90/10, 0.34/20/80, 0.36/5/95, 0.77/90/10, and 0.90/90/10, with a constant flow rate of 1.0 mL/min. Electrospray ionization was performed at 4 kV with the source temperature set to 350 °C. Nebulizer gas pressure was maintained at 60 psi, and drying gas flow was set to 12 L/min. Data were acquired in positive-ion full-scan MS mode over an m/z range of 100–3000, using a fragmentor voltage of 250 V. To minimize carryover, a blank injection consisting of running buffer was included between samples. Mass spectra were subsequently averaged and deconvoluted using Agilent MassHunter Qualitative Analysis software (version B.07.0; Agilent Technologies).

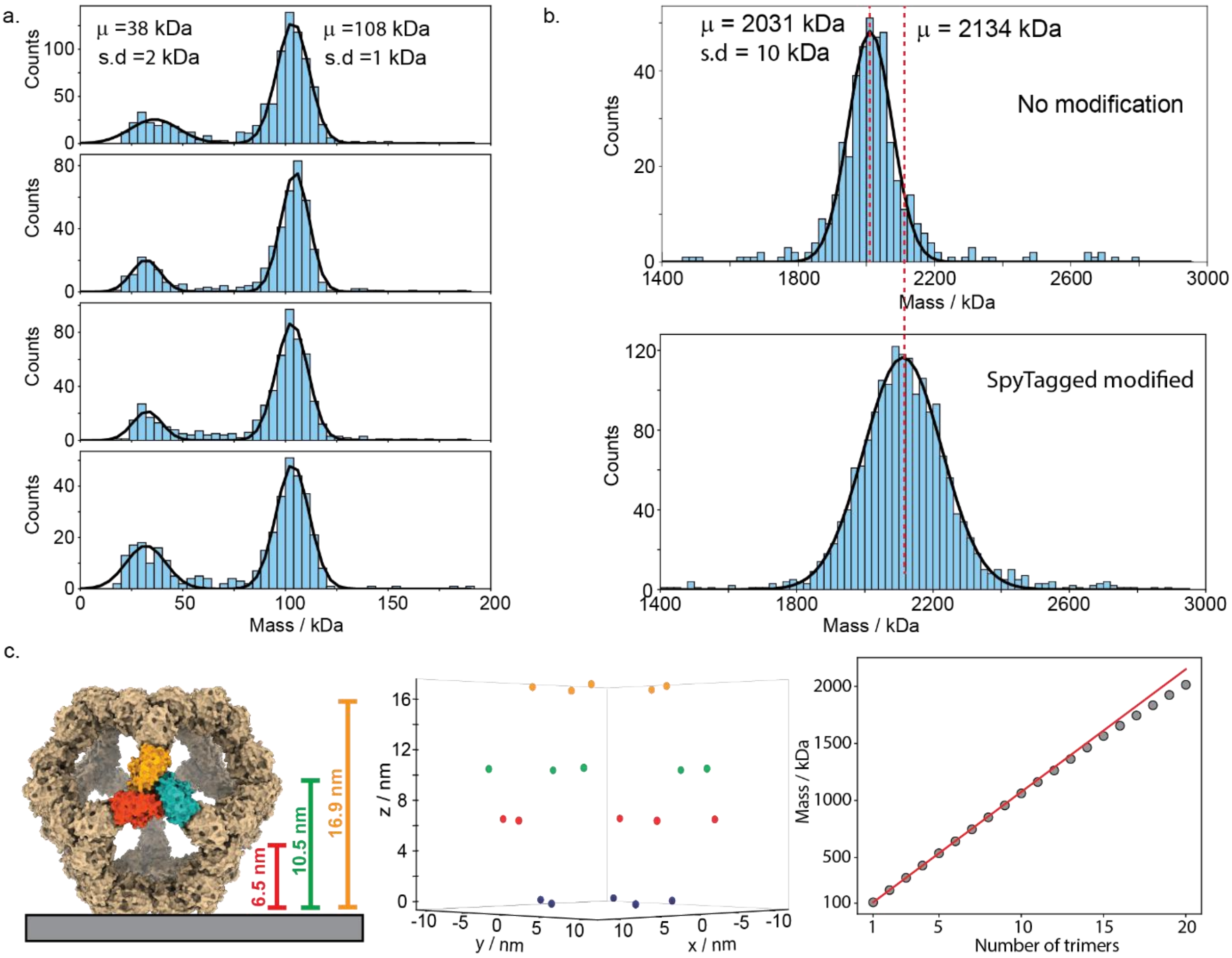


**Figure S2: Mass measurements of mi3-subunits and complete VLPs.** (a) Four representative mass histograms of mi3-monomeric and trimeric subunits following disassembly of the VLP (see Supplementary note 2). (b) Mass histograms of the complete VLPs before disassembly and the complete VLPs following the addition of excess spyTag-polyhistidine peptide (calculated mass of ~3.3 kDa per peptide). The binding of multiple peptides shifts the mass of the VLP by ~100 kDa. (c) The origin of the measured lower mass for the capsid. Left: illustration of the capsid as it expected to land on the glass surface. Owing to the dodecahedral symmetry there are 4 characteristic heights compared to the surface of the glass. The height differences between the subunits and the glass-solution interface are translated to additional phase factors between the reference light and the scattered light, resulting in lower contrast (grey symbols) than expected from 20 trimers that are bound directly to the glass surface (red curve).

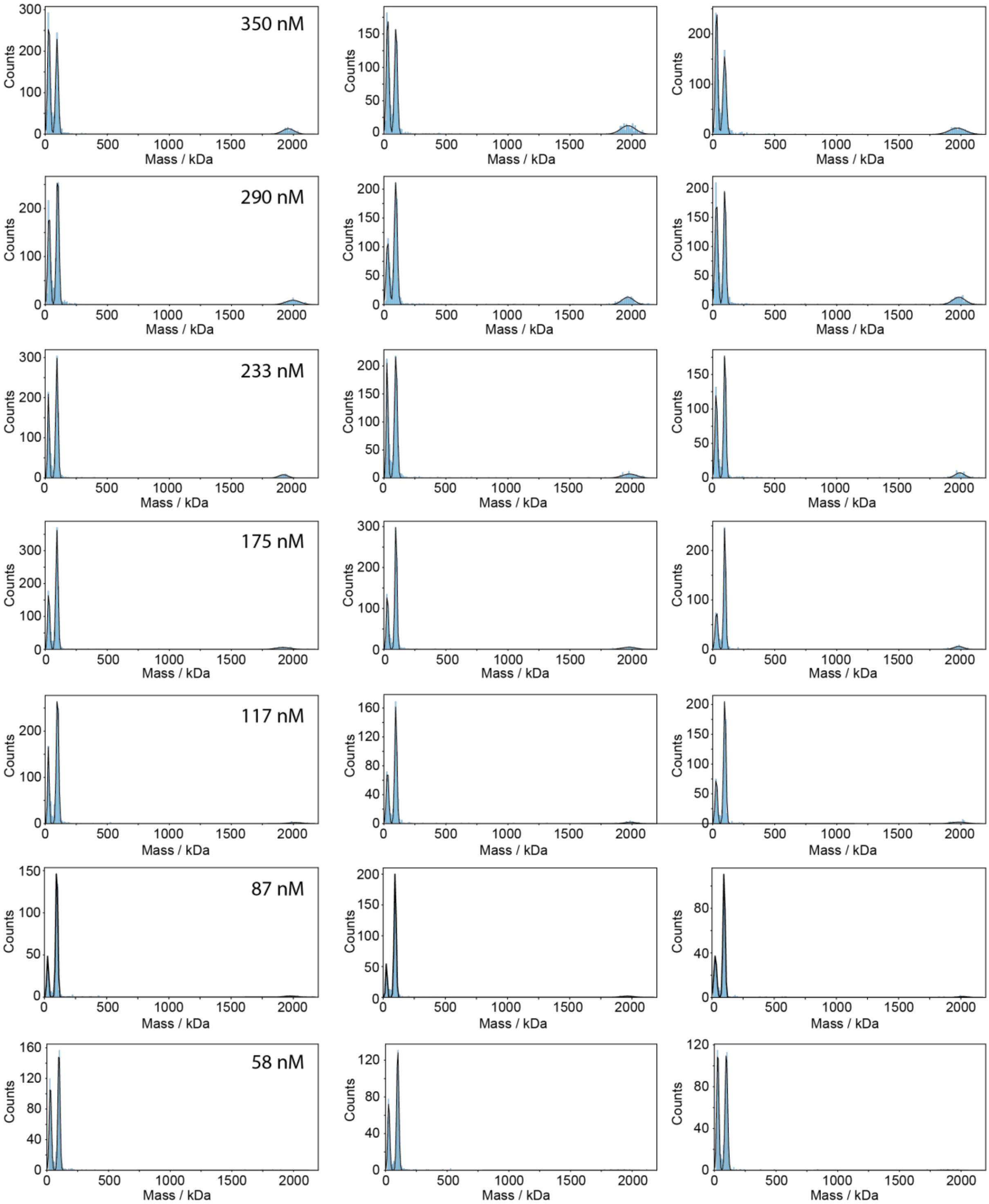


**Figure S3: Measured mass histograms of the assembly titration presented in Figure 1.** Mass histograms of equilibrated subunits in assembly buffer, measured at different total protein concentrations between 58 and 350 nM (indicated). For each protein concentration (rows), three technical replicates were measured (columns). Black curves correspond to the best three Gaussian functions fitted to each molecular state including the mi3-monomer, mi3-trimer and complete VLP. The calculated mass fraction presented in **Fig. 1d** represents the average and standard deviation of the three replicates.

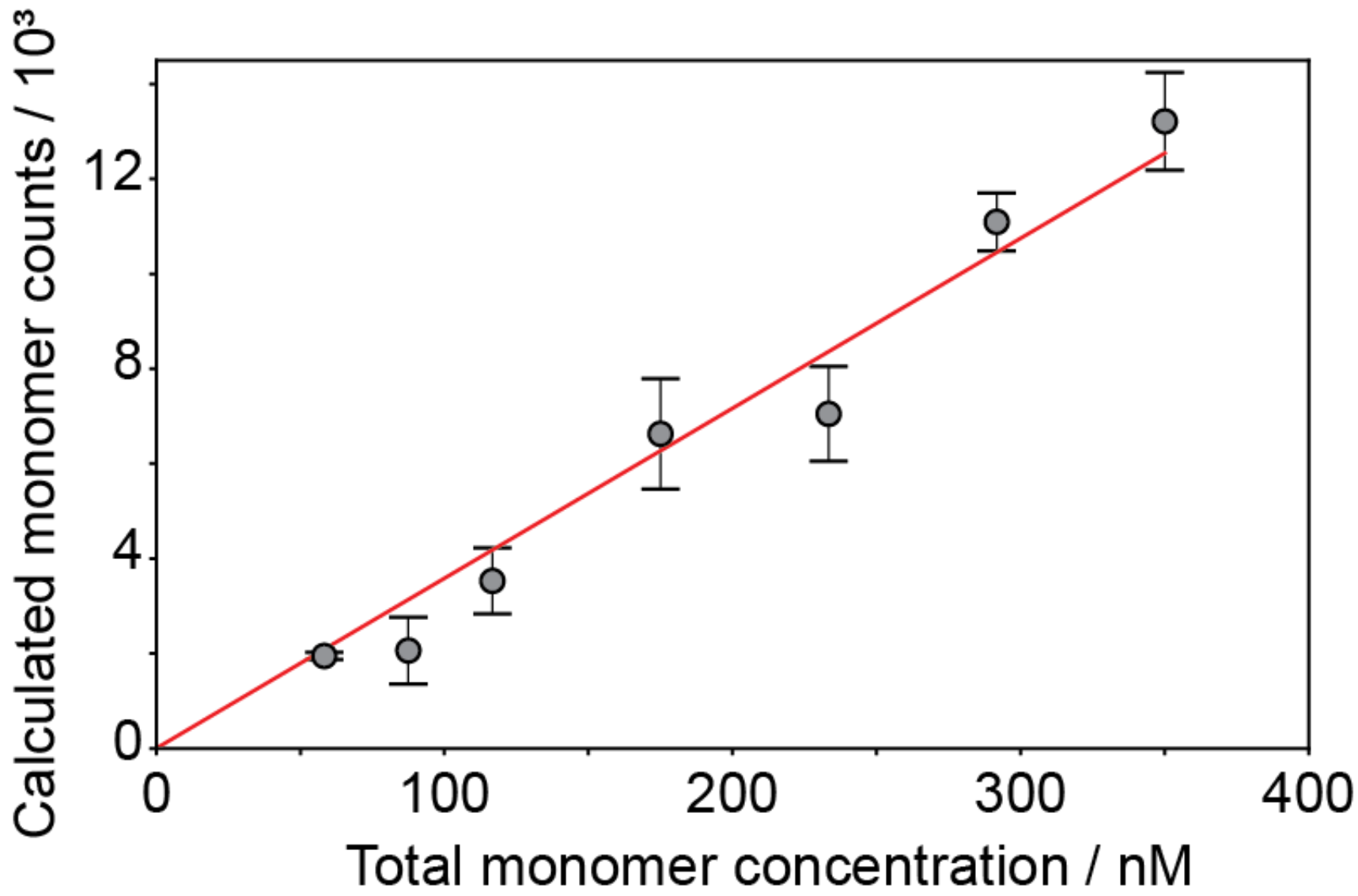


**Figure S4: Calculated total mi3-monomer detections during 60 sec acquisition MP movie as a function of total mi3-monomer concentration.** The calculated values were extracted by fitting a gaussian peak for the mi3-monomers, mi3-trimers and complete VLPs. The area under each peak corresponds to the number of detected particles. The total counts of each oligomeric state were multiplied by the aggregation number (1, 3 or 60) and the total weighted counts were summed, and presented as a function of the incubated sample concentrations.

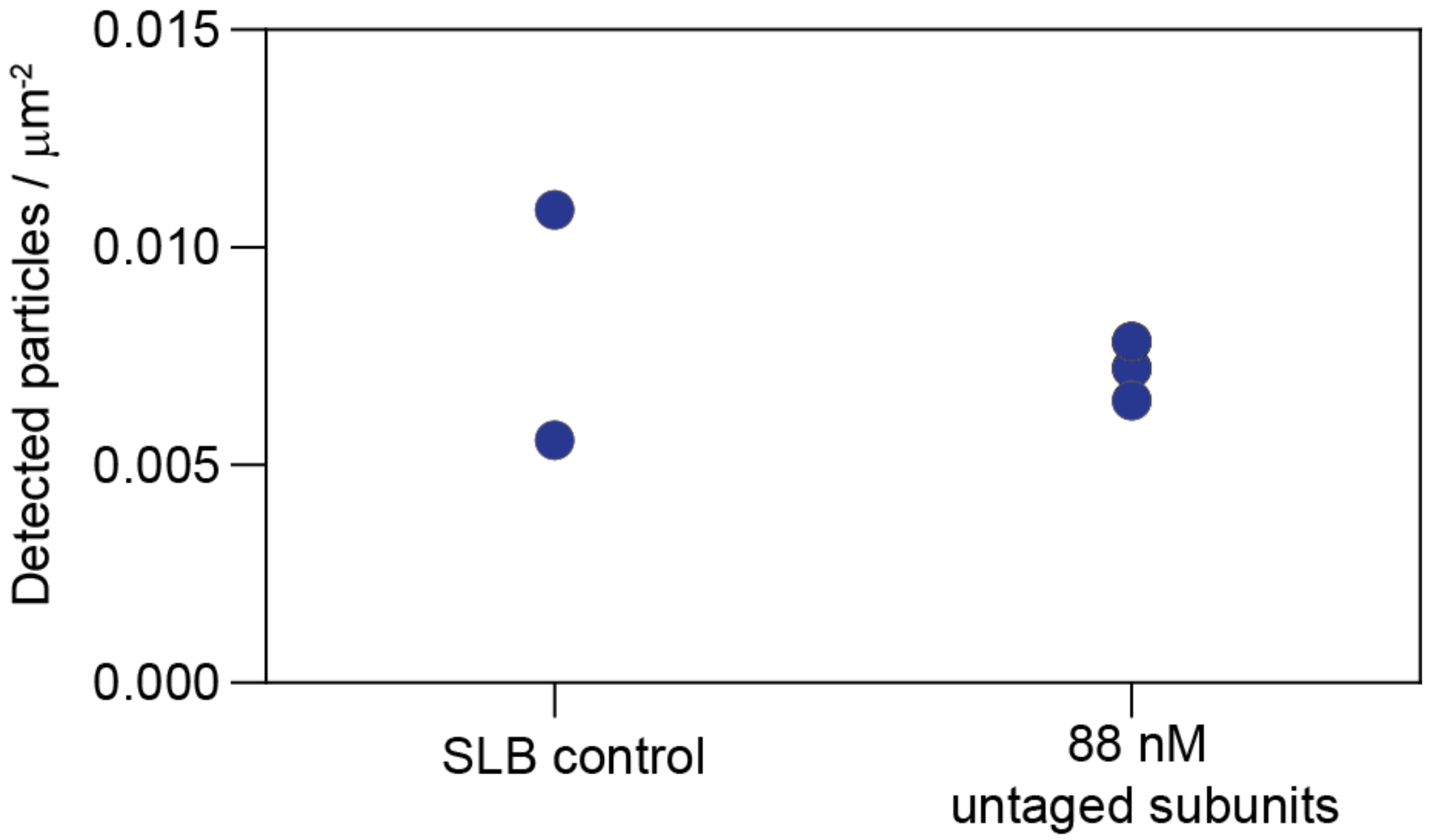


**Figure S5: Characterisation of mi3-subunits non-specific binding to the SLB** Quantification of nonspecific binding to clean SLBs showing the same and very low number of detected particles before (control) and after the addition of a solution containing a total of 88 nM of mi3-monomers.

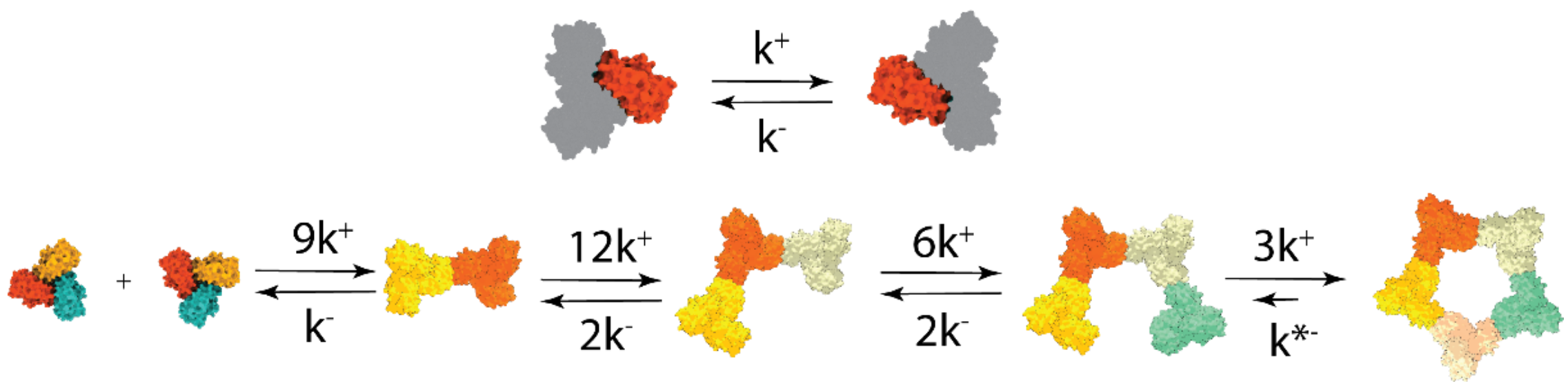


**Figure S6: Schematics of the two-dimensional model illustrating the assembly of the pentagonal ring on SLB surface.** Top: Schematic of the pairwise interaction between one interface of the mi3-trimers, given by $K_D = \frac{k^-}{K^+}$. When considering the interaction between trimers, the kinetic degeneracies should be included, owing to multivalency. Assuming an isodesmic process, the observed dissociation constant will vary owing to the differences in kinetic factors. The variation in the forward kinetic degeneracy for oligomers larger than 2 is a result of the flat configuration of the growing ring. In this configuration one free interface of the trimer, is oriented to the solution and is not available for 2D interaction. The fundamental interaction free energy change, $\Delta G^{\circ} = -k_B T \ln \frac{k^-}{\rho \cdot k^+}$ is the only free parameter in the model**.**

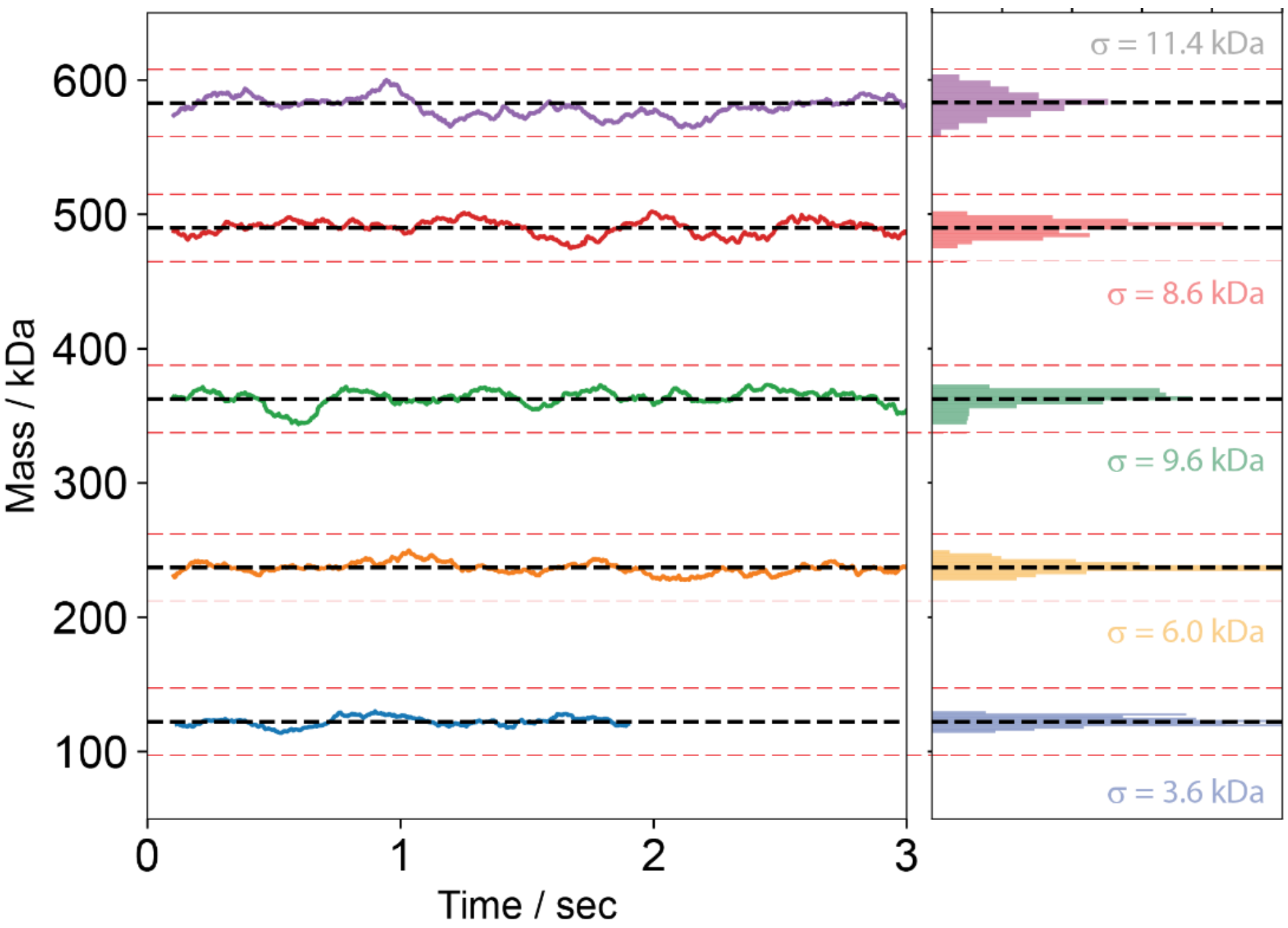


**Figure S7: Mass accuracy at 100 ms temporal resolution** Mass traces oligomeric species between mi3-trimers and pentamer of mi3-trimers on SLBs (**Fig. 2**) following rolling temporal averaging of 28 frames (effective temporal resolution of ~100 ms)**.** Mass traces were acquired at 270 Hz. Red horizontal dashed lines indicate a +-25 kDa around the average. The resulting mass histograms and standard deviations are shown to the right.

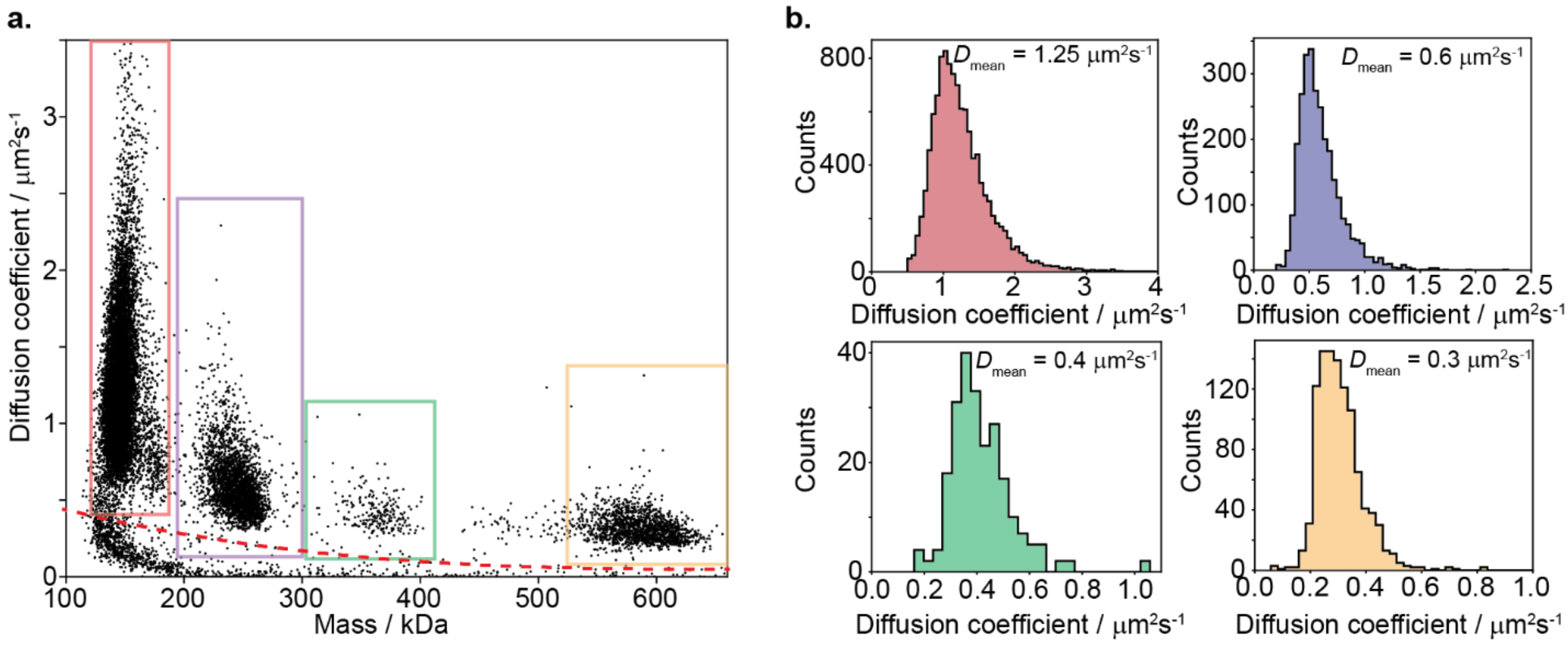


**Figure S8: Mass and diffusion measurement using dynamic-MP**. (a) Representative Mass-Diffusion scatter plot for a 1 min Dynamic-MP movie of the assembly of his-tagged mi3-trimers on an SLB (**Supplementary note 4**). Each point in the plot represents a molecular trajectory segment. Coloured boxes indicate the mass and diffusion ranges corresponding to the mi3-trimer (red), dimer of trimers (purple), trimer of trimers (green) and the pentagonal ring (orange). The red dashed line corresponds to a threshold below which trajectories are considered to represent noise from the SLB. (b) Diffusion coefficient histograms, corresponding to the data shown in the coloured boxes in (a). The mean values of the diffusion coefficient distributions are indicated.

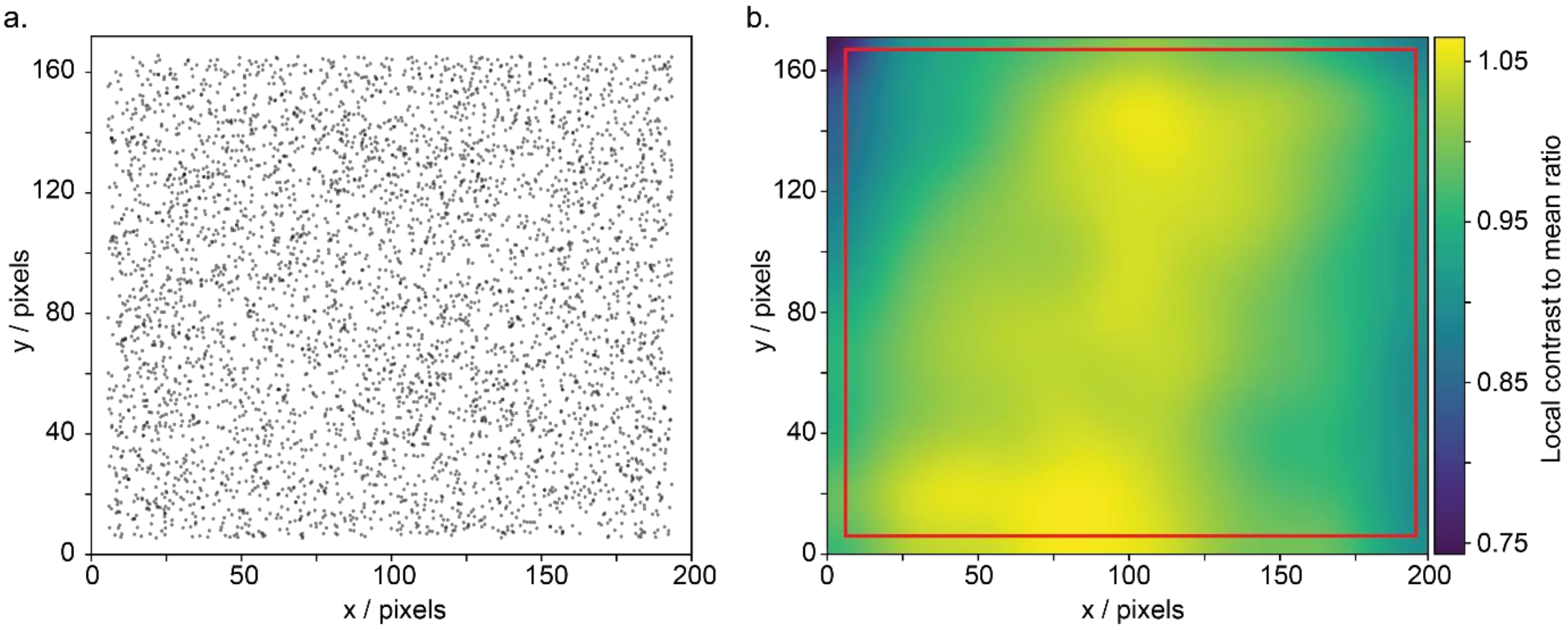


**Figure S9: Spatial contrast correction map for the large FOV used for assembly experiments on continuous SLB.** (a) Binding map of the citrate synthase protein as a function of x-y position. (b) Smoothed and normalised event - contrast map as a function of landing event positions. The red box indicates the area of the FoV utilised for tracking and quantification of the molecular masses of assembling VLPs. The map shows that except for the edges of the FOV, the contrast variation as a function of position is lower than 5%.

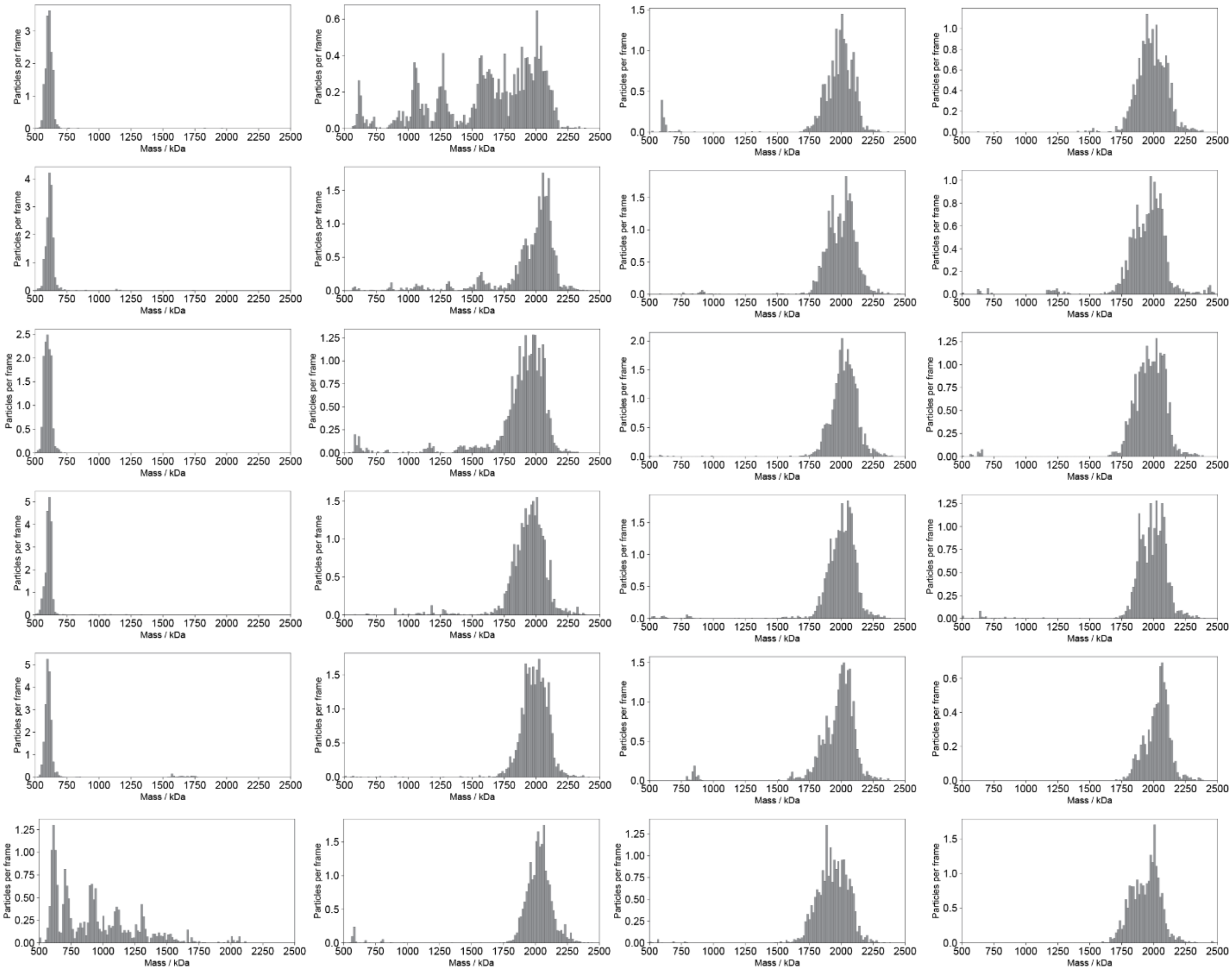


**Figure S10: Bulk assembly mass histograms for assembly reaction at 88 nM of soluble mi3 protein concentration: Repeat 1**. Measured mass histograms for assembly reaction at 88 nM total protein concentrations in solution. Each mass distribution was extracted from a 60 sec Dynamic-MP movie, acquired as described in the **Method Section**. The first 5 histograms represent the reference initial state of the pentagonal ring on the SLB before mi-3 subunits were added to solution.

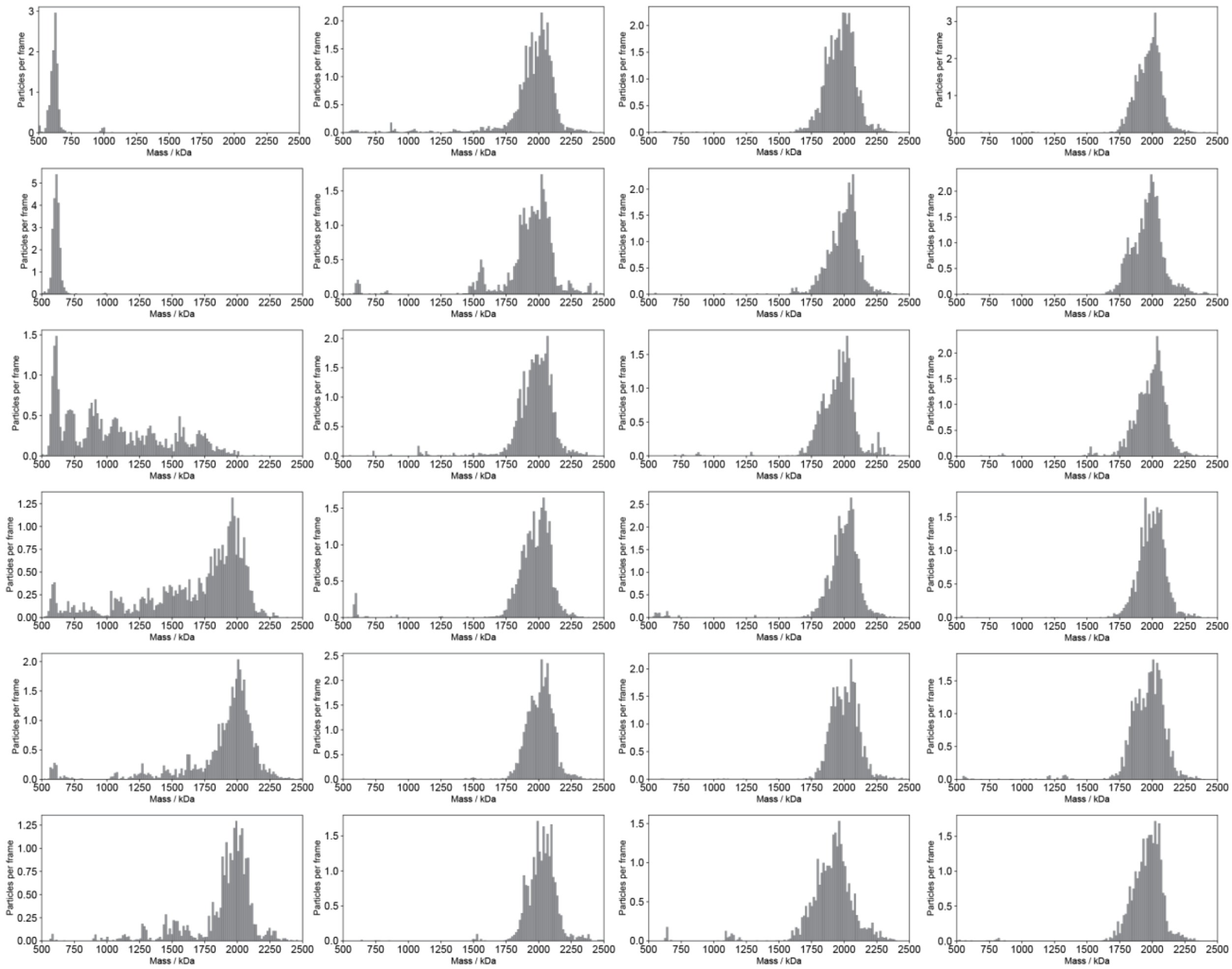


**Figure S11: Bulk assembly mass histograms for assembly reaction at 88 nM of soluble mi3 protein concentration: Repeat 2.**

Measured mass histograms for assembly reaction at 88 nM total protein concentrations in solution. Each mass distribution was extracted from a 60 sec Dynamic-MP movie, acquired as described in the **Method Section**. The first 2 histograms represent the reference initial state of the pentagonal ring on the SLB before mi-3 subunits were added to solution.

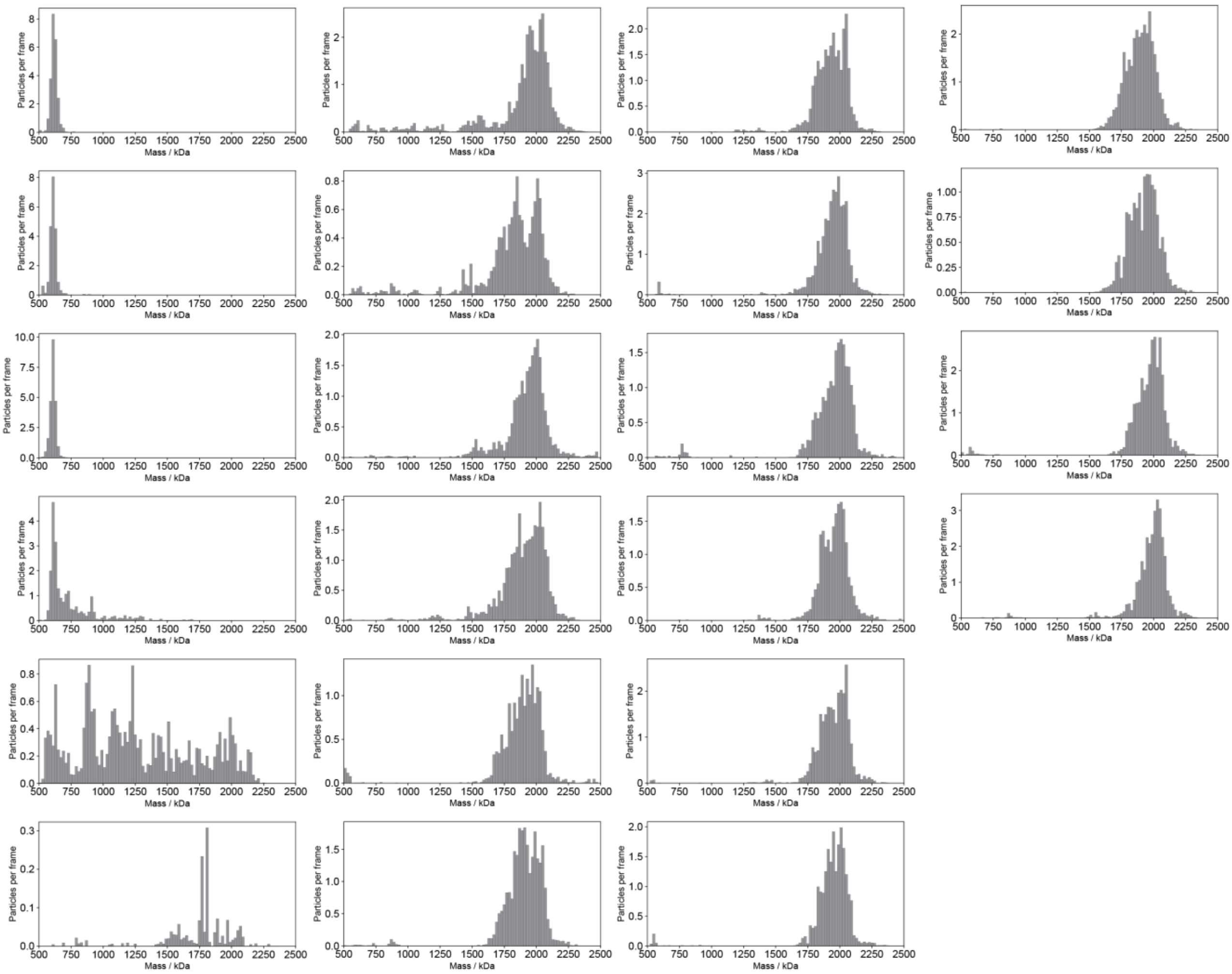

**Figure S12: Bulk assembly mass histograms for assembly reaction at 88 nM of soluble mi3 protein concentration: Repeat 2.**

Measured mass histograms for assembly reaction at 88 nM total protein concentrations in solution. Each mass distribution was extracted from a 60 sec Dynamic-MP movie, acquired as described in the **Method Section**. The first 3 histograms represent the reference initial state of the pentagonal ring on the SLB before mi-3 subunits were added to solution.

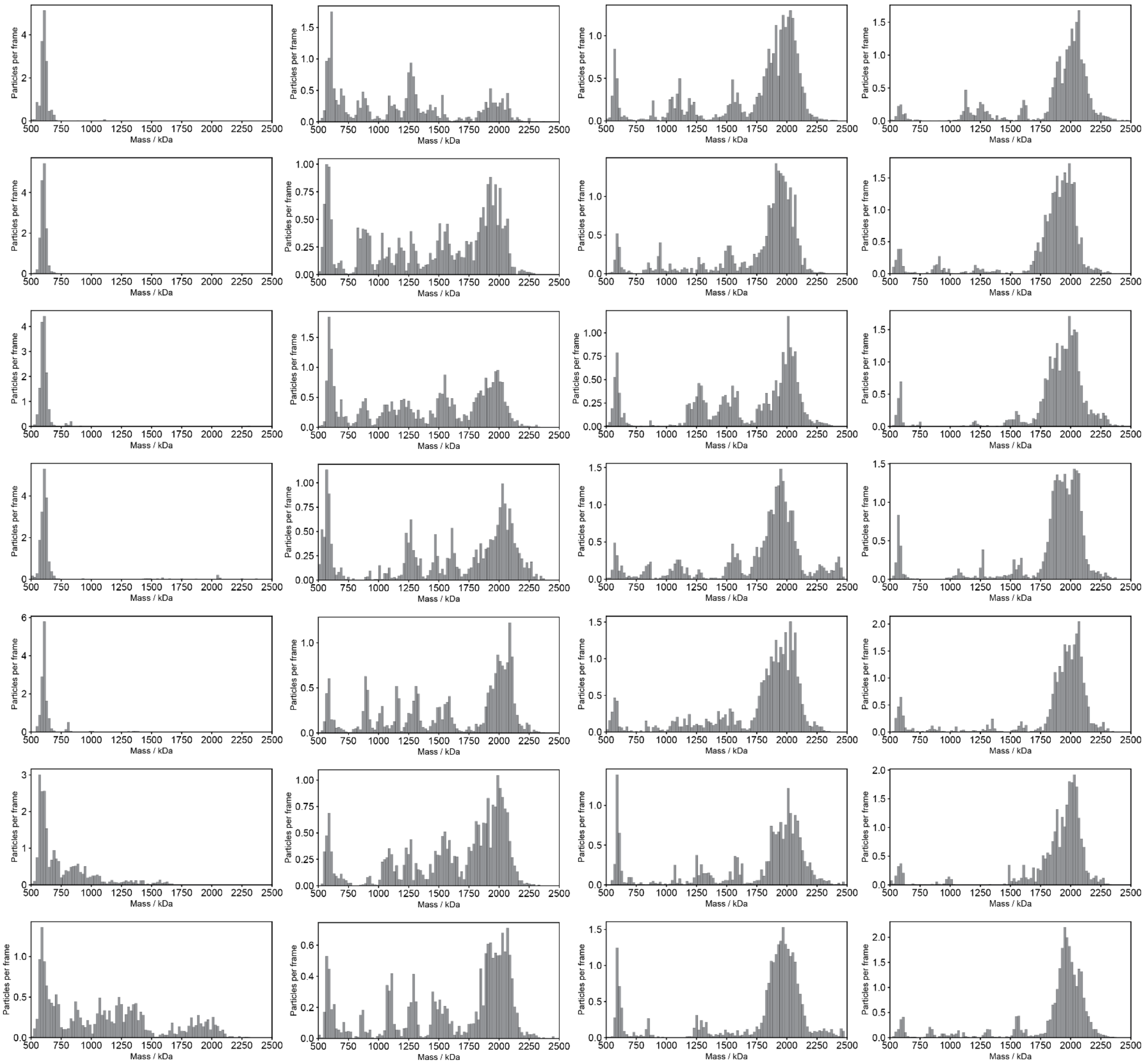


**Figure S13: Bulk assembly mass histograms for assembly reaction at 44 nM of soluble mi3 protein concentration: Repeat 1.**

Measured mass histograms for assembly reaction at 44 nM total protein concentrations in solution. Each mass distribution was extracted from a 60 sec Dynamic-MP movie, acquired as described in the **Method Section**. The first 5 histograms represent the reference initial state of the pentagonal ring on the SLB before mi-3 subunits were added to solution.

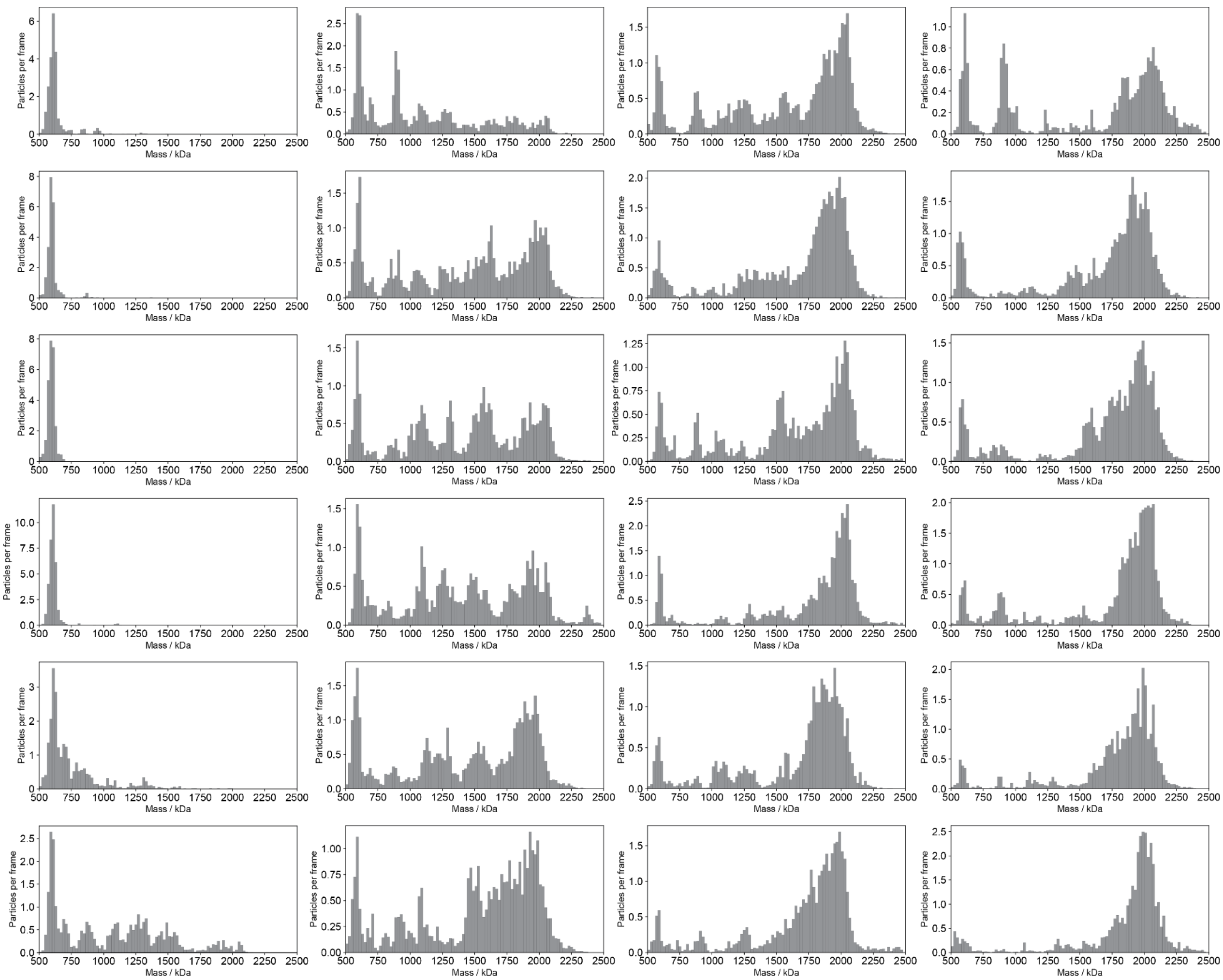


**Figure S14: Bulk assembly mass histograms for assembly reaction at 44 nM of soluble mi3 protein concentration: Repeat 2.**

Measured mass histograms for assembly reaction at 44 nM total protein concentrations in solution. Each mass distribution was extracted from a 60 sec Dynamic-MP movie, acquired as described in the **Method Section**. The first 4 histograms represent the reference initial state of the pentagonal ring on the SLB before mi-3 subunits were added to solution.

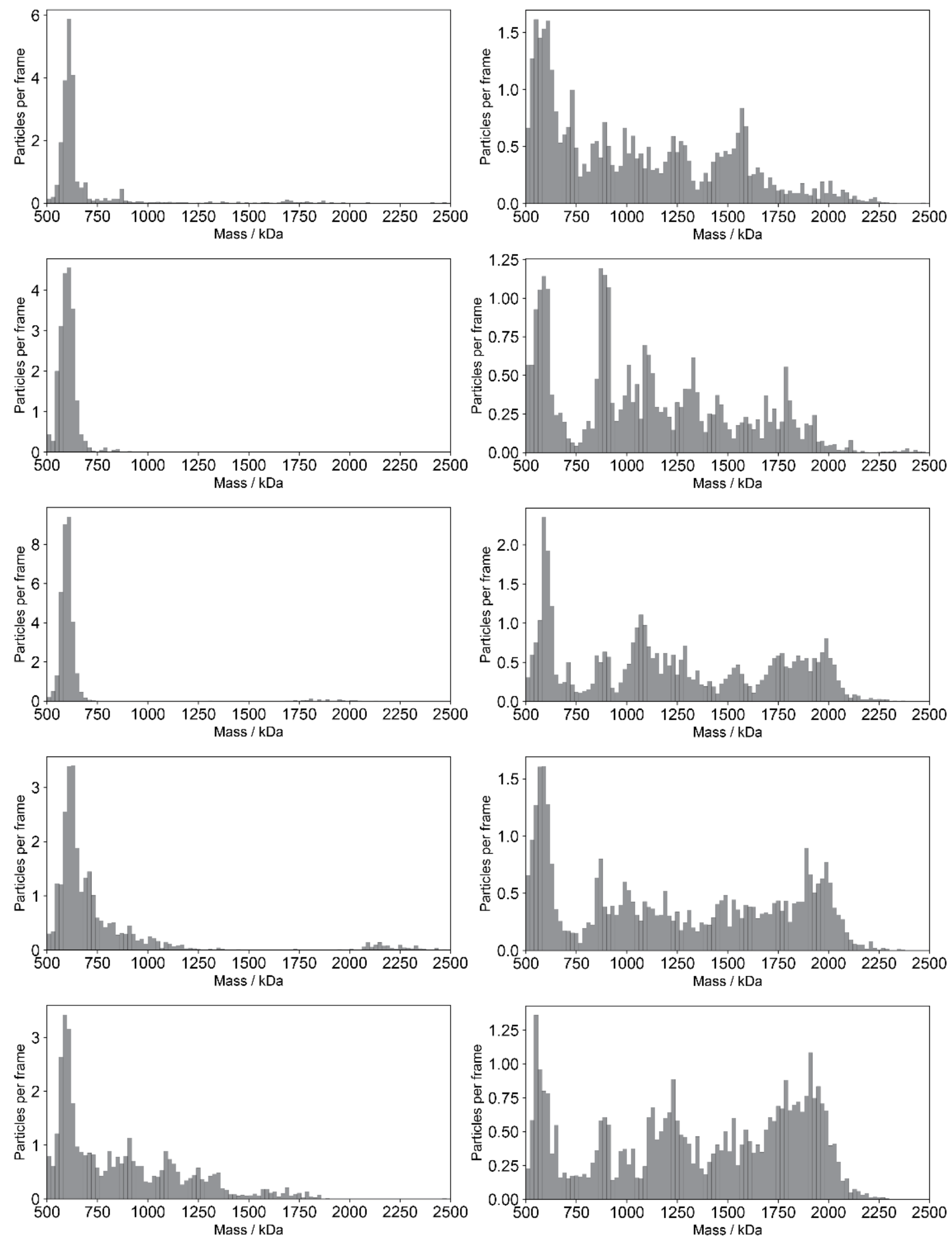


**Figure S15: Bulk assembly mass histograms for assembly reaction at 44 nM of soluble mi3 protein concentration: Repeat 3.**

Measured mass histograms for assembly reaction at 44 nM total protein concentrations in solution. Each mass distribution was extracted from a 60 sec Dynamic-MP movie, acquired as described in the **Method Section**. The first 3 histograms represent the reference initial state of the pentagonal ring on the SLB before mi-3 subunits were added to solution.

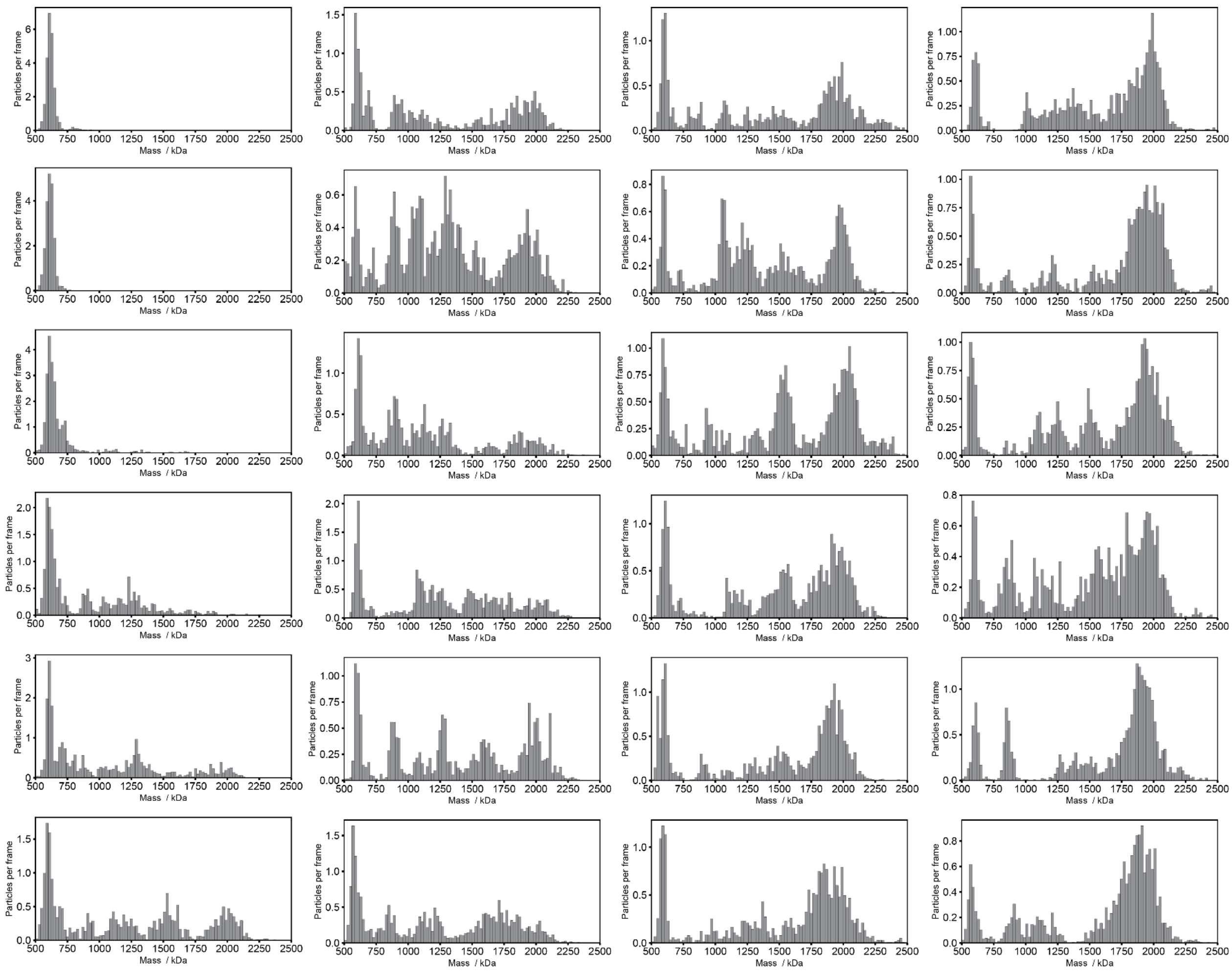

**Figure S16: Bulk assembly mass histograms for an assembly reaction at 29 nM of soluble mi3 protein concentration: Repeat 1.**

Measured mass histograms for assembly reaction at 29 nM total protein concentrations in solution. Each mass distribution was extracted from a 60 sec Dynamic-MP movie, acquired as described in the **Method Section**. The first 2 histograms represent the reference initial state of the pentagonal ring on the SLB before mi-3 subunits were added to solution.

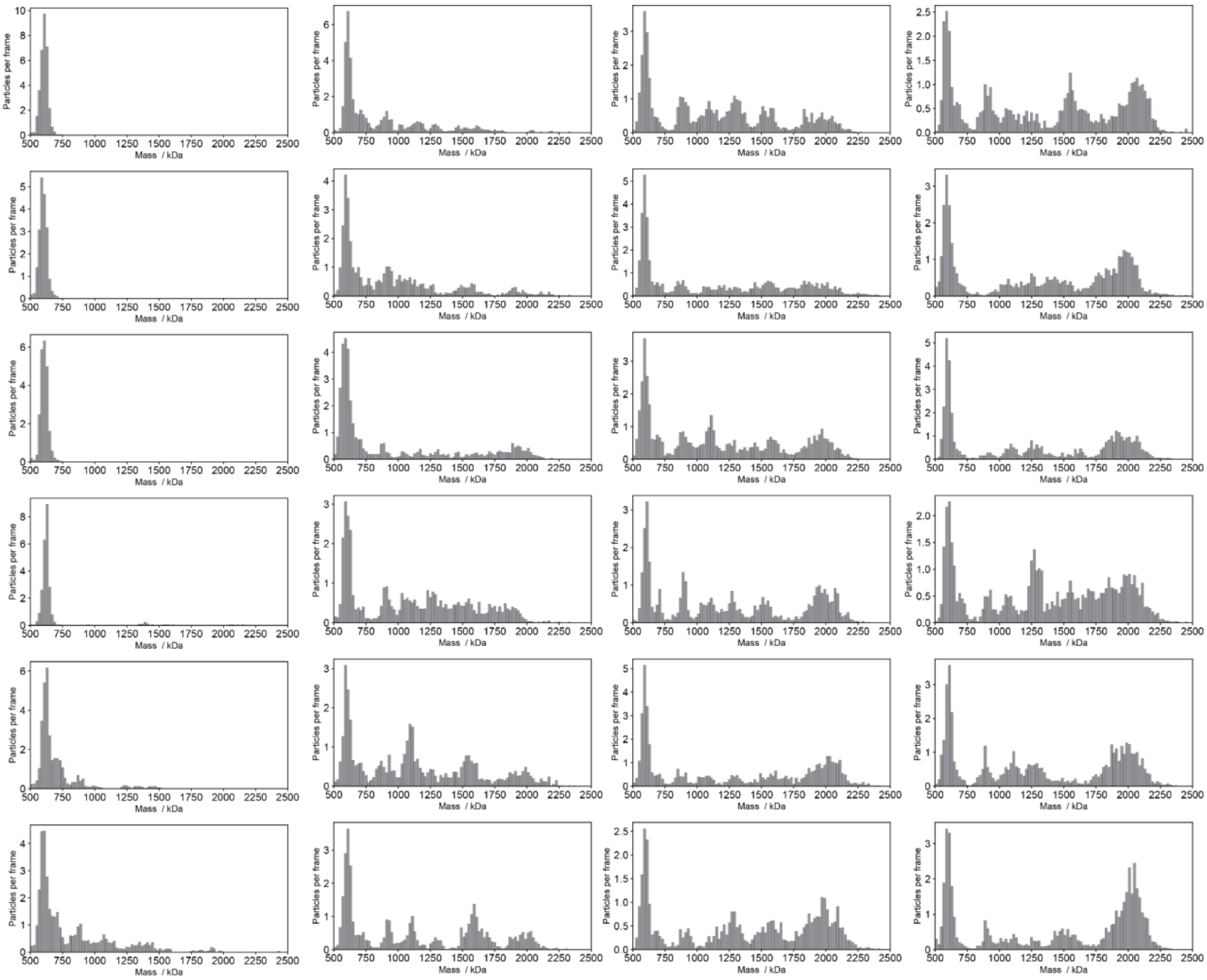


**Figure S17: Bulk assembly mass histograms for an assembly reaction at 29 nM of soluble mi3 protein concentration: Repeat 2.**

Measured mass histograms for assembly reaction at 29 nM total protein concentrations in solution. Each mass distribution was extracted from a 60 sec Dynamic-MP movie, acquired as described in the **Method Section**. The first 4 histograms represent the reference initial state of the pentagonal ring on the SLB before additional mi-3 subunits were added to solution.

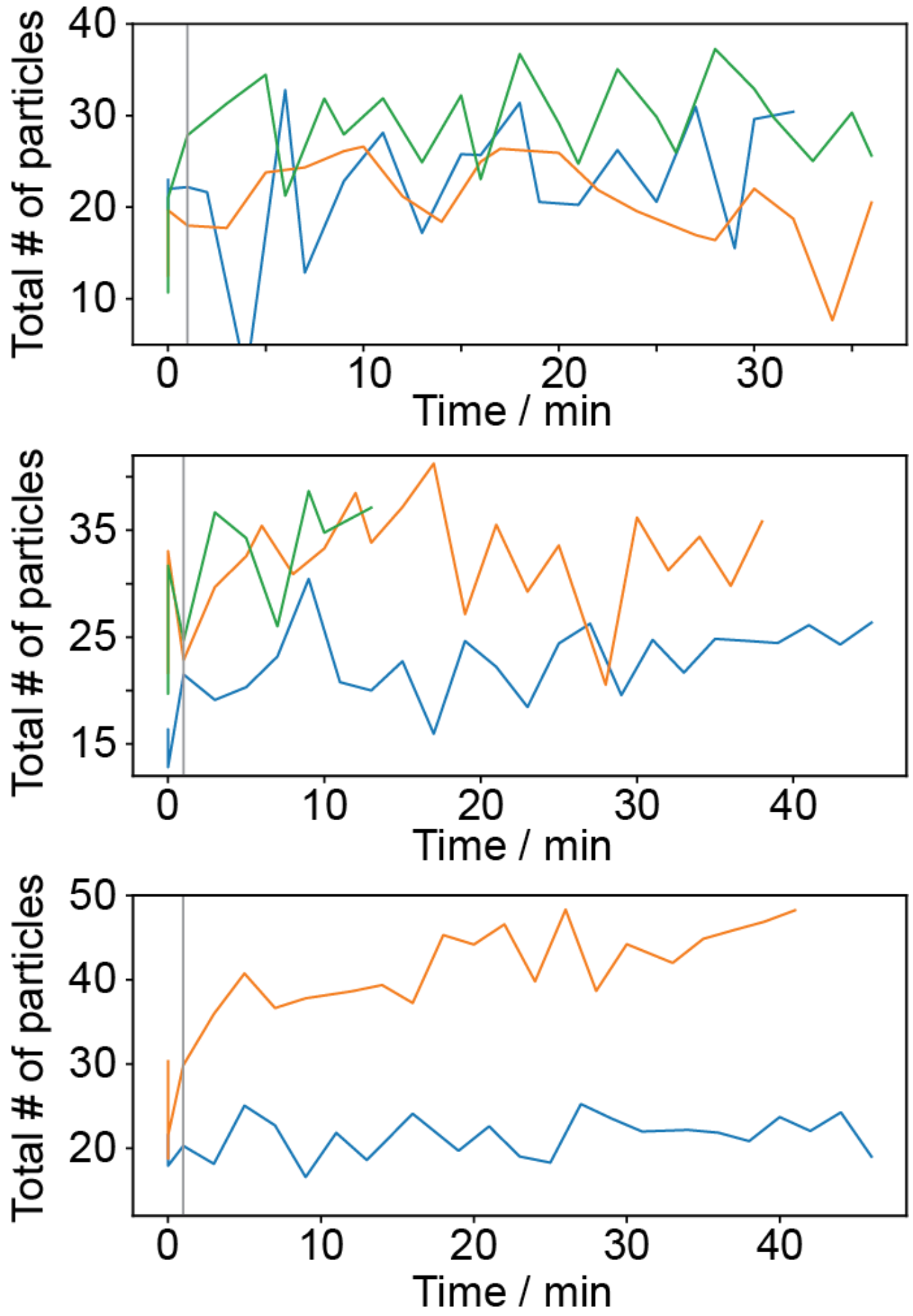


**Figure S18: Adding unlabelled mi3-proteins on top of the SLB-confined pentamers does not change the surface density of detected particles throughout the assembly reaction.** The total number of particles observed on the SLB as a function of time during the experiments described **in Fig. 3** and **S10-17**. Each colour represents a technical repeat, where the top, middle and bottom panels correspond to the experiments done at total mi3-monomer solution concentrations of 88, 44, and 29 nM. Grey vertical lines correspond to the time points where the unlabelled mi3-proteins were introduced to the solution.

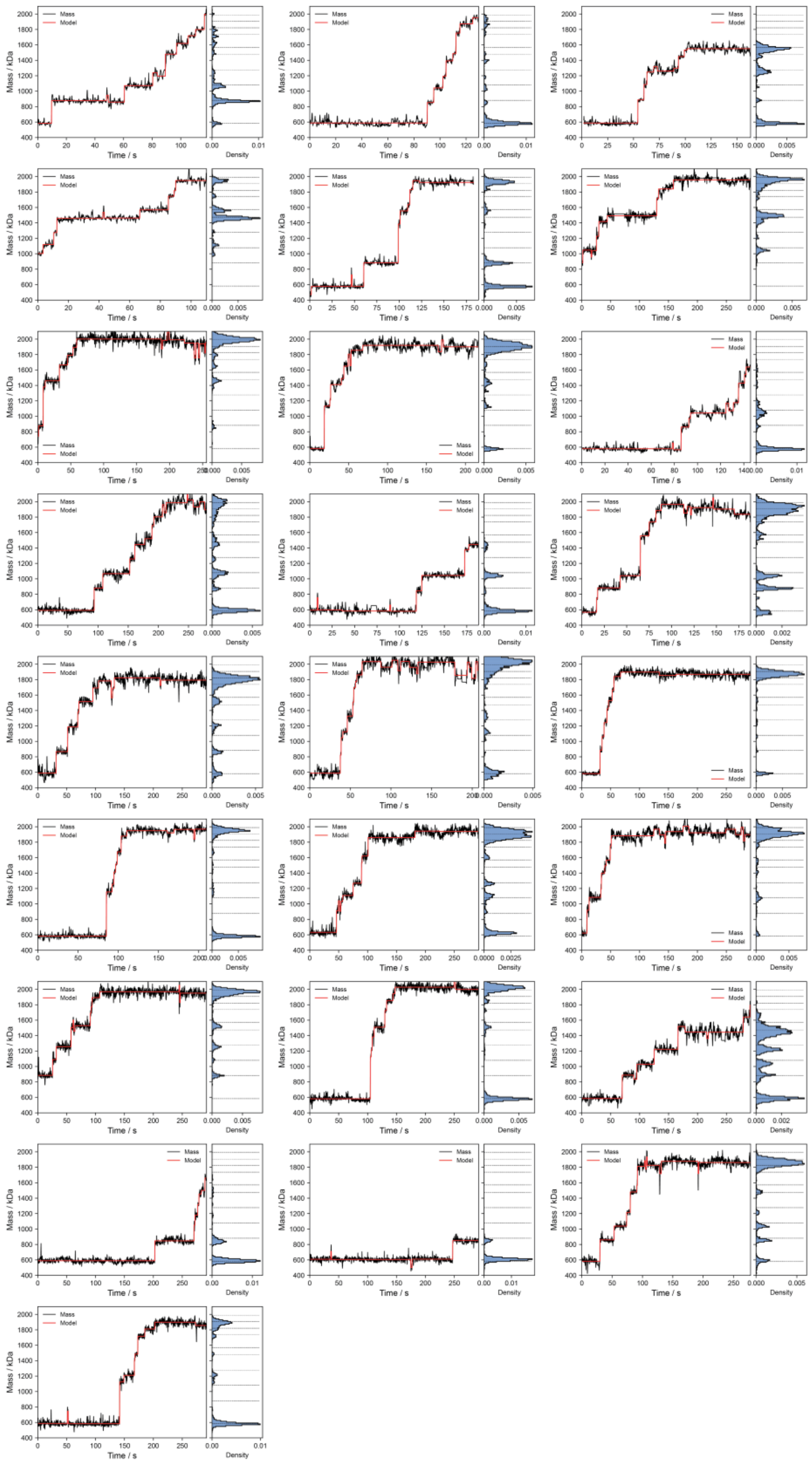


**Figure S19: Measured mass traces of the assembly process of mi3-VLPs in confined SLBs.** Each mass trace corresponds to a single VLP assembly process and contains: the measured mass trace as a function of time (black curve) following a moving median operation with a symmetric window size of 50 frames (200 ms) from each side, a step detection model (the **Method Section**) that identifies transitions between oligomeric states (red curve), and a projected mass histogram. Dashed lines in the histogram, indicate the expected massed of the topologically closed states

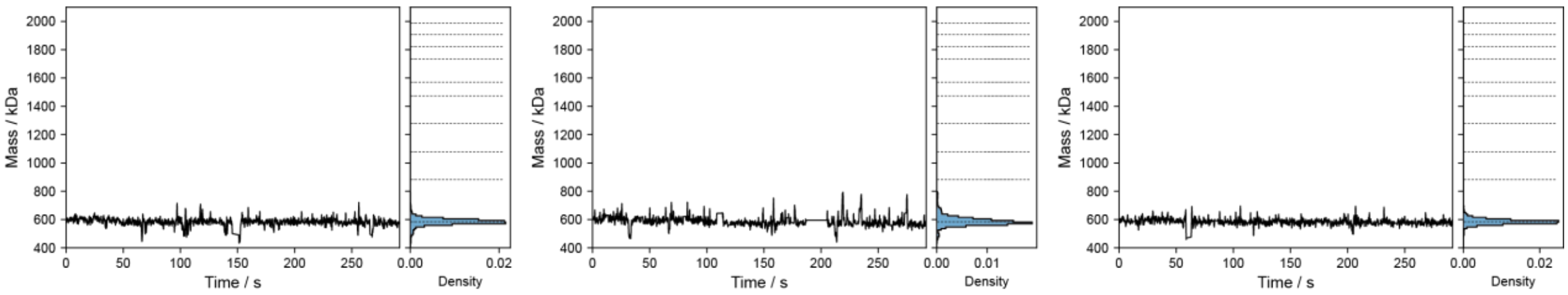


**Figure S20: Measured mass traces of mi3-pentamers in confined SLBs that did not assemble during 5 min of the acquisition.** Each mass trace corresponds to individual mis-pentamer and contains the measured mass trace as a function of time (black curve) following a moving median operation with a symmetric window size of 50 frames (200 ms) from each side and a projected mass histogram. Dashed lines in the histogram, indicate the expected massed of the topologically closed states.

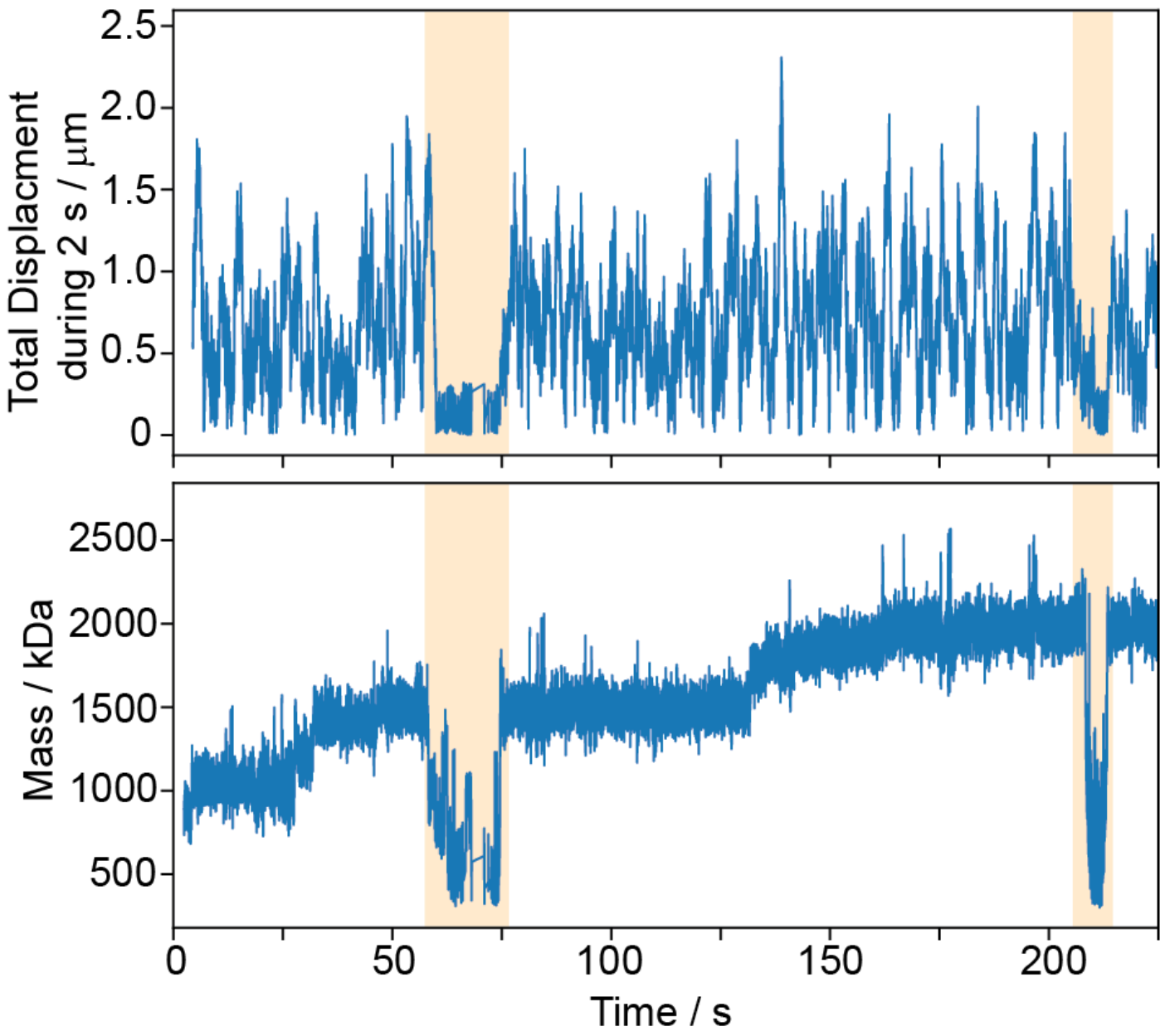


**Figure S21: Transient immobilisation of particles leads to an underestimation of the measured contrast.** As a result of the ratiometric background subtraction (see Methods), transient immobilisation of particles on timescales comparable to the time window used for the median background calculation results in a reduced optical contrast. The top panel shows an example of transient particle immobilisation events, identified by the total displacement measured over 2 s intervals as a function of observation time. The bottom panel shows the mass estimate, based on the ratiometric contrast, for the same particle. Two major transient immobilisation events are highlighted, illustrating the correlation between transient immobilisation and the measured contrast.

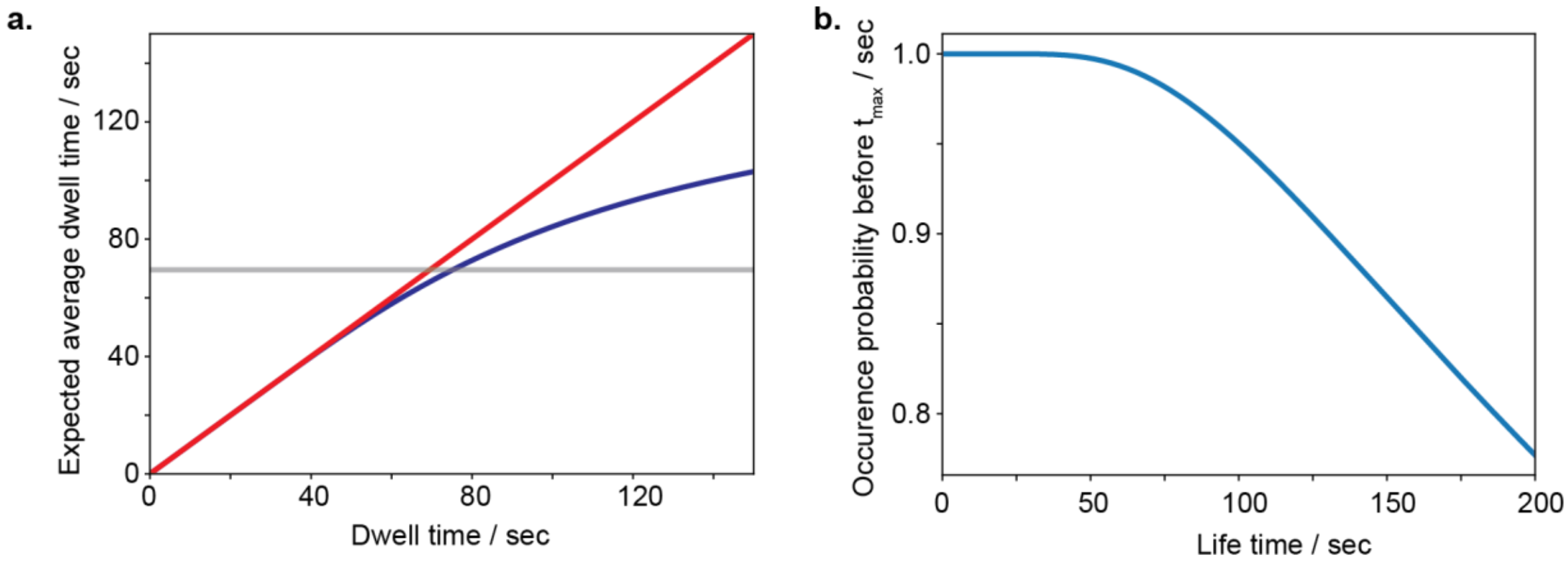


**Figure S22: The measured averaged dwell time of the pentamer represents a lower bound.** The experimentally measured average dwell time of the pentameric ring represents a lower bound on the true lifetime because the observation window is finite (20–300 s after addition of mi3 subunits) (a) Assuming single-exponential kinetics, the initial 20 s delay does not affect the measured dwell time because only particles that remained in the pentameric state during this period were included in the analysis. However, transitions occurring after the end of the observation window are not observed. The average observed dwell time within this finite observation window is therefore given by,

$$< \tau_{observed} > = \frac{\int_0^{t_{max}} \frac{t}{\tau} \exp\left(-\frac{t}{\tau}\right) dt}{\int_0^{t_{max}} \frac{1}{\tau} \exp\left(-\frac{t}{\tau}\right) dt}.$$

where $t_{max}$ correspond to the beginning and end of the observation window, respectively.

The difference between the true dwell time, $\tau$, and the theoretically observed $\tau_{observed}$ therefore becomes significant for dwell times approaching $t_{max}$. The grey horizontal line indicates the experimentally measured pentamer dwell time, demonstrating that the measured value represents a lower estimate of the true lifetime. (b) We can also estimate the upper bound by calculating the probability of observing a transition from the pentameric ring to a higher-order intermediate during the measurement window as a function of the dwell time. Because approximately **90**% of pentameric rings were observed to grow beyond the pentameric state during the experiment, the corresponding analysis places an upper bound of approximately **125** sec on the pentamer dwell time at a mi3 monomer concentration of 88–100 nM.

## Supplementary references


1 Zlotnick, A. To Build a Virus Capsid. *Journal of Molecular Biology* **241**, 59–67 (1994). https://doi.org/10.1006/jmbi.1994.1473

2 Yogurtcu, O. N. & Johnson, M. E. Theory of bi-molecular association dynamics in 2D for accurate model and experimental parameterization of binding rates. *The Journal of Chemical Physics* **143** (2015). https://doi.org/10.1063/1.4929390

3 Wu, Y., Vendome, J., Shapiro, L., Ben-Shaul, A. & Honig, B. Transforming binding affinities from three dimensions to two with application to cadherin clustering. *Nature* **475**, 510–513 (2011). https://doi.org/10.1038/nature10183

4 Gavutis, M., Jaks, E., Lamken, P. & Piehler, J. Determination of the Two-Dimensional Interaction Rate Constants of a Cytokine Receptor Complex. *Biophysical Journal* **90**, 3345–3355 (2006). https://doi.org/10.1529/biophysj.105.072546

5 Jhaveri, A., Chhibber, S., Kulkarni, N. & Johnson, M. E. Protein dimerization in 2D vs 3D: Geometric allostery enhances binding affinity. *The Journal of Chemical Physics* **163** (2025). https://doi.org/10.1063/5.0275282

6 Fleming, P. J. & Fleming, K. G. HullRad: Fast Calculations of Folded and Disordered Protein and Nucleic Acid Hydrodynamic Properties. *Biophysical Journal* **114**, 856–869 (2018). https://doi.org/10.1016/j.bpj.2018.01.002

7 Asor, R. *et al.* Assembly Reactions of Hepatitis B Capsid Protein into Capsid Nanoparticles Follow a Narrow Path through a Complex Reaction Landscape. *ACS Nano* **13**, 7610–7626 (2019). https://doi.org/10.1021/acsnano.9b00648

8 Zandi, R., Reguera, D., Bruinsma, R. F., Gelbart, W. M. & Rudnick, J. Origin of icosahedral symmetry in viruses. *Proceedings of the National Academy of Sciences* **101**, 15556–15560 (2004). https://doi.org/10.1073/pnas.0405844101

9 Ben-Shaul, A. & Gelbart, W. M. in *Micelles, Membranes, Microemulsions, and Monolayers Partially Ordered Systems* Ch. Chapter 1, 1–104 (1994).

10 Gillespie, D. T. Stochastic Simulation of Chemical Kinetics. *Annual Review of Physical Chemistry* **58**, 35–55 (2007). https://doi.org/10.1146/annurev.physchem.58.032806.104637
