# Supplementary material for: Molecular-level observation of the self-assembly of a virus-like particle
Source: Nature. 2026 Sep 16;657(8132):653–60. doi: 10.1038/s41586-026-10948-z (PMC13581590; doi:10.1038/s41586-026-10948-z)
Supplement: Supplementary file 2 [file 41586_2026_10948_MOESM2_ESM.pdf]

Peer Review File

Molecular-level observation of the self-assembly of a virus-like particle

Corresponding Authors: Roi Asor & Philipp Kukura

Any redactions in this file are there to maintain patient confidentiality, the confidentiality of unpublished data, or to remove third-party material.

This file contains all reviewer reports in order by version, followed by all author rebuttals in order by version.

Attachments originally included by the reviewers as part of their assessment can be found at the end of this file.

Version 0:

Reviewer comments:

Referee #1

(Remarks to the Author)
The manuscript by Asor, Loewenthal, and co-workers describes a stunningly detailed series of observations of virus assembly using a cutting-edge, label-free, single-molecule technique. The key enabler of these observations is the combination of mass photometry, and use of a supported lipid bilayer for prolonged measurements. Throughout all measurements, in multiple concentration regimes, mass photometry allows the evolution of the mass, and thus monomer count, to be quantified in beautiful detail.
A major strength of the manuscript is its rigorous investigation over multiple concentration regimes. First, the assembly process is investigated at equilibrium (Fig 1), where it is conspicuously observed that mi3 monomers and mi3 trimers are observed, as well as fully assembled capsids, but nothing in between. Second, the effective concentration is enhanced through confinement to 2D, and the assembly of pentamers of trimers is characterized in detail (Fig 2,3). Finally, at a further elevated effective concentration, the assembly of pentamers of trimers is monitored (Fig 4,5) to capture assembly of the full 60-subunit capsid (15 mi3 trimers). Thus, the whole process is captured from beginning to end.
To achieve the needed high effective concentration, assembly is studied in 2D. In Lines 150-157, the authors lay out their evidence that this confinement does not alter the intrinsic molecular reactivity. I find their arguments to be persuasive.
I am particularly enthusiastic about Figure 4c, that depicts the excellent agreement between observed populations in the grand canonical ensemble and the expected free energy landscape. This is a highly successful example of how new label-free methods can allow observation of dynamics that have previously only been simulated or only studied in a more limited manner. The authors' comment at the end, that essentially any self-assembled nanostructure (of sufficient size) can be probed in this manner, suggests this result is a harbinger of many more studies of this kind: the first in a new wave of observations on molecular self-assembly of exceptional and unprecedented detail.
I enthusiastically recommend publication after the issues discussed below are addressed. Particularly important issues are starred*.
General comment: I urge the authors to be more consistent (in text and figures) about how they refer to oligomers and monomers. Sometimes monomer means the mi3 monomer, and sometimes it means the trimer. A 5-mer could mean 5 or 15 mi3 monomers! This can usually be understood from context, but it would be nice if the reader didn't have to work as hard. Some consistent terminology (for example, "pentamer of trimers" so that monomer only refers to mi3, but other schemes are certainly possible) would be helpful.
Line 62, what does "self-limited" mean in this context?
*Line 67-69, Of references 42-45, 42/43/45 are not single-molecule studies (as the beginning of this line states). Ref 47, which uses a related technique, could use more discussion. Finally, the authors highlight the limits of studies on 2D substrates, but also carry out their own work on 2D substrates. Looking through these references, it is very clear how much of a leap this study is over the state of the art. Nonetheless, I think the reader would benefit from some additional discussion of these past experiments and contextualization of their results.
Line 80, I do not understand why the authors call this "solution mass photometry", since it seems like this is the standard geometry of mass photometry (except for the SLB) where the measurement entails capture of the solution-phase species at the surface. Saying "solution" here implies this is a different configuration of MP. The reader would also benefit from a

reference here for MP (the only previous one is in the abstract).
Line 81, when “single-molecule” is used as a modifier (here, of “trapping approach”), it should be hyphenated.
Line 96, “standard” MP is not a helpful descriptor for the non-expert. Perhaps, MP on a glass substrate?
Line 97-98, the authors should list the MP-determined mass alongside the known value for easy comparison, for the monomer, trimer, and full capsid. These exact values are hard to get from Fig. 1.
Line 98-100, there is something confusing about how this sentence is worded.
Line 102 and Fig 1, I could not understand how the fully assembled capsid could be 15% when there are just 4 blips detected, but then I realized that each blip accounts for 60 monomers. One way the authors could help the reader to perceive this is to add the mass integration of these peaks (similar to how integrals are presented in an NMR trace) in the bottom traces of Fig. 1b (where an integral over the 4 blips would be shown to have a comparable value to, say, the integral over the monomer peak). Also, the concentrations (58 nM/350 nM) in Fig. 1b should be enlarged and moved away from the scale bar so that they are not confused with a scale bar length. In Fig. 1d, a legend should be included within the figure for easy reference. What do the shaded colors represent?
*Line 107, can these equilibrium constants be compared to literature values?
Line 127-131, as it is worded, this sentence implies that the increase in effective concentration derives from the constrained rotation, but it actually comes from the reduction of dimensionality. Also, the authors say “mi3-subunits do not interact with the SLB in the absence of a tag” but do not present evidence. Looking through the SI, perhaps Fig. S7 should be referred to here.
Line 139, as I understand, there is precedent for the longevity of the pentamer of trimers, as in Ref 51. This should be acknowledged.
Line 235-237, I don’t understand this argument about supersaturation. Supersaturation is not mentioned in Note 8.
Fig. 4, the authors should include the number of individual trajectories to produce the panels in Figures b and c.
*Line 298 and surrounding area, the kinetic analysis in Figure 5 is exquisite, but I think it could be used in a somewhat richer manner. If one takes the dwell times in Fig. 5c, and simulates the equilibrium populations, do you get the relative populations in Fig. 4c? And if not why?
*Also, the authors should show (perhaps in SI), a histogram of dwell times for each stable intermediate (ie, the entries on the x axis in Fig. 5c). The average dwell time only gives the rate for a monoexponential decay process. Eyeballing the density of circles, it looks like some of the kinetics here may be non-monoexponentially distributed, but it is of course hard to see. If the decay kinetics are non-monoexponential, then it would not be terribly surprising, since they know that each irreversible event includes multiple reversible intermediates. Still, I think the authors may have some additional information they can extract about those hard-to-see intermediates (for example, a minimum “waiting time” before the irreversible step can be completed. I appreciate that the dwell times for the pentamer of trimers is skewed because of the 20s “dead time” of the experiment, but it looks particularly non-monoexponentially distributed.
Figure 6, I found part b) to be difficult to parse because the x and y axes are different between the three panels.
Line 348-349, I understand that it is expected that the reaction rates scale in this manner, but I don’t believe the authors have measured this. If so, this should be presented as an “expected” dependence, rather than something that has been confirmed.
SI, Line 739, typo, “of” vs “if”

Referee #2

(Remarks to the Author)
Asor et al. report an elegant and seminal set of experiments to monitor assembly of virus-like particles. This has been a dream experiment of many scientists for decades. Using mass photometry they are able to monitor and quantify each step, i.e. building block addition, especially neat is the assembly in SLB traps. The manuscript is extremely well written and data clearly presented. The conclusions are fully supported by the data. The results are relevant to a broad readership and impacts multiple fields. The approach will likely be applied to virus assembly or also other hetero-oligomeric machineries in the future. It is recommended to publish the manuscript in Nature after minor revisions addressing the following comments.
Minor comments:
-line 107 - please use the same naming convention for the KDs as in the figures
-supplement shows minor errors such as pixel x pixel is displayed in different layouts, suggested to unify the appearance, line 473 - "10 higher" is 10x higher meant?, line 550 - should be Fig. S17b, and would benefit from an additional round of proof-reading
-Fig S1c middle - the plot seems skewed with a dip in the middle of the x axis
-font size in main text figures is very small, sometimes too small

Referee #3

(Remarks to the Author)
A. This study investigates the assembly pathways of an engineered protein into a dodecahedral cage by directly observing the process at the single-particle level. Using mass photometry combined with single-molecule trapping, the authors track the growth of individual protein intermediates in real time and measure their molecular mass throughout the process.
B. The claim that "no experimental approach has been successful in visualizing capsid assembly" and the limitations of other methods have been overcome by the present work is debatable. Garmann et al (2019, ref. 47) have used a similar approach to observe the kinetics of assembly of single virus capsids around viral RNA. Although that reference lacked the

level of detail of this manuscript, it should be emphasized that ref. 47 used a real virus which had an icosahedral cage with 2649 possible intermediate species, while the current manuscript deals with a dodecahedral cage with 73 possible intermediate species. The significance of the work for virus assembly is not clear. The construct is not a capsid. Instead, it is a highly engineered, wireframe protein dodecahedral cage that structurally resembles more to a clathrin cage than a virus. There are no dodecahedral viruses, to my knowledge so the title should be revisited. The building blocks of the dodecahedral cage are large and few and that is probably the main reason the same method has produced a clearer picture than Garmann et al in terms of the assembly pathway are the nature of intermediates. In addition, single molecule visualization with time course structural information has been demonstrated on surface supported, flat HIV coat protein assemblies (ref. 50, ACS Nano 2020, 14, 7, 8724–8734). Lower time resolution, but much higher spatial resolution than the current work. Granted, that system was also very engineered and a surface support was needed, not unlike the current work.

The system under study is an engineered protein cage under surface-confined conditions, with no nucleic acid involvement. As such, additional controls and comparisons are needed to establish how well the results translate to natural virus assembly, particularly given differences in geometry, molecular interactions, and the absence of nucleic acids or other viral components. Several aspects of the experimental design, analysis, and interpretation limit the generalizability of the conclusions.

C.-D. The approach and the chosen system are perfectly matched to maximize the amount of information that could be extracted by using the mass photometry method. The quality of data is mostly high, with some exceptions where statistics are questionable. Specifically, Fig. 1 b: fully assembled VLPs representing 15 +/- 4% is a conclusion drawn from 1-2 counts. Describing the statistics as insufficient would be an understatement.

Reducing dimensionality from 3d to 2d allows mass photometry to access oligomerization dynamics, but also increases the artificial nature of the study.

Mass photometry provides information on particle mass but not structure. Consequently, identical masses may correspond to distinct structural states and intermediate assignments rely on assumed geometries rather than direct observation. While the mi3-cage structure is known from prior work, the study does not include any structural validation (e.g., EM, AFM) of the assembled species under the experimental conditions. Perhaps, for mi3 subunits which are very rigid conformation is not supposed to change, but for viruses the ability to switch between conformers is a characteristic required by quasi-symmetry.

The analysis assumes that landing events faithfully reflect solution-phase concentrations. Is there an experimental basis for this assumption? Surface-based detection may introduce biases due to differential surface affinity of species and possible distortion upon surface interaction. These factors could affect the relative abundance of oligomeric states, particularly for weak or transient intermediates. The manuscript does not explicitly address or control for such biases.

E. The authors extract key thermodynamic parameters (e.g., dissociation constants and pseudo-critical concentration), but these values are not directly compared to those reported for well-characterized viral systems such as HBV, MS2, CCMV or BMV or consistent with other, established ensemble-averaging methods.
As a result, it is unclear whether:
- The interaction strengths fall within physiologically-relevant ranges
- The observed assembly pathways are representative of viral capsids
- The inferred mechanisms are broadly applicable or system-specific
- The observed kinetics are realistic
These omissions weaken the claim that the study reveals general principles of virus assembly.

Generalizing the pairwise-additive interaction assumption might be a stretch. Is there support for this assumption? There have been suggestions in the literature that this is not the case for viruses.

G. The discussion of prior literature occasionally emphasizes limitations of previous approaches without highlighting their contributions. A more balanced framing would improve context.

H. The introduction is prone to generalizations, e.g. L 53 on assembly efficiency: Depending on what is understood by “efficient”, this generalization might be a stretch. There are several well-studied spherical viruses with symmetrical capsids for which assembly also produces non-infectious particles including sub-viral particles a defective particles. Examples: HBV, Influenza, Paramyxoviruses (Senday, Measles), and a few retroviruses. Judging from the reference list, it would seem that HBV is the canonical icosahedral virus. However, a few of the features of assembly that are mentioned as general, have been identified much earlier in other systems, e.g. Prevelige, P. E., Jr., Thomas, D., & King, J. (1993). Nucleation and growth in bacteriophage P22 assembly. Biophysical Journal. A generalization of the accepted mechanism for empty capsid formation is on L 63: "key intermediates are short-lived and of very low abundance". It should be clear, this is not true for viruses: See the tobacco mosaic virus - classic textbook example, 34-subunit coat protein disks (the key intermediate) form first, then viral RNA binds to a specific site on a disk, and the ring rearranges into a helical intermediate. Several experimental and theoretical works of in vitro co-assembly involving the encapsulation of cargo (cognate or not) have shown that there are alternate pathways to the “low-abundance key intermediates” one. References to these works are conspicuously absent. There are some very strong claims in conclusion - if there are no limits why not study a real virus?

Open Access This Peer Review File is licensed under a Creative Commons Attribution 4.0 International License, which permits use, sharing, adaptation, distribution and reproduction in any medium or format, as long as you give appropriate credit to the original author(s) and the source, provide a link to the Creative Commons license, and indicate if changes were made.
In cases where reviewers are anonymous, credit should be given to 'Anonymous Referee' and the source.
The images or other third party material in this Peer Review File are included in the article's Creative Commons license, unless indicated otherwise in a credit line to the material. If material is not included in the article's Creative Commons license and your intended use is not permitted by statutory regulation or exceeds the permitted use, you will need to obtain permission directly from the copyright holder.
To view a copy of this license, visit https://creativecommons.org/licenses/by/4.0/

Referees' comments:
Referee#1
The manuscript by Asor, Loewenthal, and co-workers describes a stunningly detailed series of observations of virus assembly using a cutting-edge, label-free, single-molecule technique. The key enabler of these observations is the combination of mass photometry, and use of a supported lipid bilayer for prolonged measurements. Throughout all measurements, in multiple concentration regimes, mass photometry allows the evolution of the mass, and thus monomer count, to be quantified in beautiful detail. A major strength of the manuscript is its rigorous investigation over multiple concentration regimes. First, the assembly process is investigated at equilibrium (Fig 1), where it is conspicuously observed that mi3 monomers and mi3 trimers are observed, as well as fully assembled capsids, but nothing in between. Second, the effective concentration is enhanced through confinement to 2D, and the assembly of pentamers of trimers is characterized in detail (Fig 2,3). Finally, at a further elevated effective concentration, the assembly of pentamers of trimers is monitored (Fig 4,5) to capture assembly of the full 60-subunit capsid (15 mi3 trimers). Thus, the whole process is captured from beginning to end.

To achieve the needed high effective concentration, assembly is studied in 2D. In Lines 150-157, the authors lay out their evidence that this confinement does not alter the intrinsic molecular reactivity. I find their arguments to be persuasive.

I am particularly enthusiastic about Figure 4c, that depicts the excellent agreement between observed populations in the grand canonical ensemble and the expected free energy landscape. This is a highly successful example of how new label-free methods can allow observation of dynamics that have previously only been simulated or only studied in a more limited manner. The authors' comment at the end, that essentially any self-assembled nanostructure (of sufficient size) can be probed in this manner, suggests this result is a harbinger of many more studies of this kind: the first in a new wave of observations on molecular self-assembly of exceptional and unprecedented detail.
I enthusiastically recommend publication after the issues discussed below are addressed. Particularly important issues are starred*.

We thank the reviewer for their detailed examination of our work and for their positive perception of the presented work and its implication. Below we address their comments and suggestions.

1. General comment: I urge the authors to be more consistent (in text and figures) about how they refer to oligomers and monomers. Sometimes monomer means the mi3 monomer, and sometimes it means the trimer. A 5-mer could mean 5 or 15 mi3 monomers! This can usually be understood from context, but it would be nice if the reader didn't have to work as hard. Some consistent terminology (for example, "pentamer of trimers" so that monomer only refers to mi3, but other schemes are certainly possible) would be helpful.

Thank you, we have edited the text to be consistent with our terminology. The terminology defined as follows: mi3-monomer – for the smallest subunit, mi3-trimer – for the trimeric building block, mi3-VLP – for the assembled structure. Larger oligomers of trimers are referred according to the number of mi3-trimes.

2. Line 62, what does "self-limited" mean in this context?
*This was a typo and we refer to **self-limiting** assembly, a self-assembly process that results in a finite size assembled structure, as discussed in the references at the end of the sentence.*

To correct this, we modified the sentence as follows:

*"…First, the **self-limiting** free energy landscape, **results in a well-defined finite and closed complex, and** involves weak and reversible pairwise interactions[34-37]…."*

*3. Line 67-69, Of references 42-45, 42/43/45 are not single-molecule studies (as the beginning of this line states). Ref 47, which uses a related technique, could use more discussion. Finally, the authors highlight the limits of studies on 2D substrates, but also carry out their own work on 2D substrates. Looking through these references, it is very clear how much of a leap this study is over the state of the art. Nonetheless, I think the reader would benefit from some additional discussion of these past experiments and contextualization of their results.

Thank you, reference 45 (ion mobility MS) was misplaced here, this was corrected and moved to the previous sentence. We agree that references 42/43 (CDMS) are not classical solution phase single-molecule studies, however these references refer to CDMS experiments, an important approach used to examine the assembly mechanism of capsid proteins. Furthermore, CDMS is fundamentally a single-molecule method as it measures the mass/charge ratio and the charge of individual proteins or complexes in the gas phase. Its disadvantage with respect to our work is that dynamics are only monitored at the population level on the ~minutes time scale. Regarding reference 47, which describes iSCAT experiments, we agree with the reviewer and have modified the text to further discuss the difference in capabilities that enabled the current work.

Regarding the limitation on 2D, it is important to note that although we used a bilayer surface to monitor the assembly of the pentamer of trimers, and to keep the imaged complex at the interface, we do study the complete 3D assembly process from the pentamer to the 3D structure of the complex formed in solution. Any interaction beyond the pentamer represents solution kinetics and thermodynamics. In this sentence, we wanted to state that previous work was limited to measuring interactions that occur **only on the surface** where it was not possible to track the 3D assembly of the capsid.

Starting from line 70:

"…Single-molecule ***approaches have been applied to overcome such heterogeneity: Charge detection mass spectrometry (CDMS) and resistive-pulse sensing provide*** *ensemble-averaged snapshot observations* ***of a subset of*** *stable intermediates,* ***but struggle with*** *the temporal resolution* ***required to capture dynamics and, in the case of CDMS, does not operate in solution***[42-44]*.* ***Single-molecule fluorescence yields dynamics but cannot quantify the degree of oligomerisation***[45,46,47] ***while atomic force spectroscopy, reveals structure and interactions at the protein subunit scale only in 2D, where full capsids cannot assemble***[48,49]*. In terms of monitoring capsid assembly, interferometric scattering microscopy has been used to monitor* ***the formation of capsids on an RNA template***[49]***, yielding information on nucleation and elongation at the single particle level. This study, however, did not possess the temporal resolution and sensitivity required to reveal molecular level details of the assembly process. As*** *a result, despite decades of dedicated study, no experimental approach has been successful in visualising* ***the molecular mechanism underlying*** *capsid assembly*[13]*."*

4. Line 80, I do not understand why the authors call this "solution mass photometry", since it seems like this is the standard geometry of mass photometry (except for the SLB) where the measurement entails capture of the solution-phase species at the surface. Saying "solution" here implies this is a different configuration of MP. The reader would also benefit from a reference here for MP (the only previous one is in the abstract).

Thank you for the comment, we removed the word solution, the method is also referenced here.

5. Line 81, when “single-molecule” is used as a modifier (here, of “trapping approach”), it should be hyphenated.

Thank you, corrected

6. Line 96, “standard” MP is not a helpful descriptor for the non-expert. Perhaps, MP on a glass substrate?

We have clarified the description of the experiment.

Line 110 was changed to:

“We begin with a characterisation of the assembly process in solution using standard mass photometry, ***where individual proteins and complexes bind non-specifically from solution to a glass surface.****”*

7. Line 97-98, the authors should list the MP-determined mass alongside the known value for easy comparison, for the monomer, trimer, and full capsid. These exact values are hard to get from Fig. 1.

We added the measured values of the major solution species to the text, as well as added a measured mass spectra of the monomers using mass spectrometry (Figure S1). We are also referring the reader to Supplementary note 1 and Figure S2 to explain the conversion of the optical contrast to mass for the specific case of VLPs.

Changes in the text in line 111: “*At 58 nM total mi3-monomer concentration, the solution distribution is dominated by* ***mi3-monomers*** *and* ***mi3-trimers*** *at 38 ± 2 kDa (see also* ***Fig. S1****) and 108 ± 1 kDa, respectively (****Fig. 1b****, top and* ***Figs. S2-S3****). Plotting the mass distribution on a logarithmic scale (Fig. 1b, middle)* ***shows the extremely low abundance of intermediates in the 150 - 500 kDa mass range, as well as*** *particles near the measured mass of* ***complete mi3-VLPs (2030 ± 10 kDa, see Supplementary note 1 and Fig. S2b)****. Conversion* ***of particle abundances*** *into mass fractions (Fig. 1b, bottom,* ***where each species was weighted according to its oligomeric state****) shows that mi3-monomers are predominantly found in trimeric form (65 ± 4%) followed by fully assembled VLPs (15 ± 4%), monomers (11 ± 3%) and small oligomers (9 ± 3%) (Fig. 1b, bottom). At a higher total monomer concentration (350 nM), the majority of protein is now found in assembled VLPs (82 ± 5%,* ***at 1982 ± 25 kDa****) (Fig. 1c).* ***The small deviation of the average mass of assembled particles (1982 vs 2030 kDa) could be caused by a fraction of particles missing one trimeric subunit caused by slow kinetics associated with capsid closure***[57]*.*”

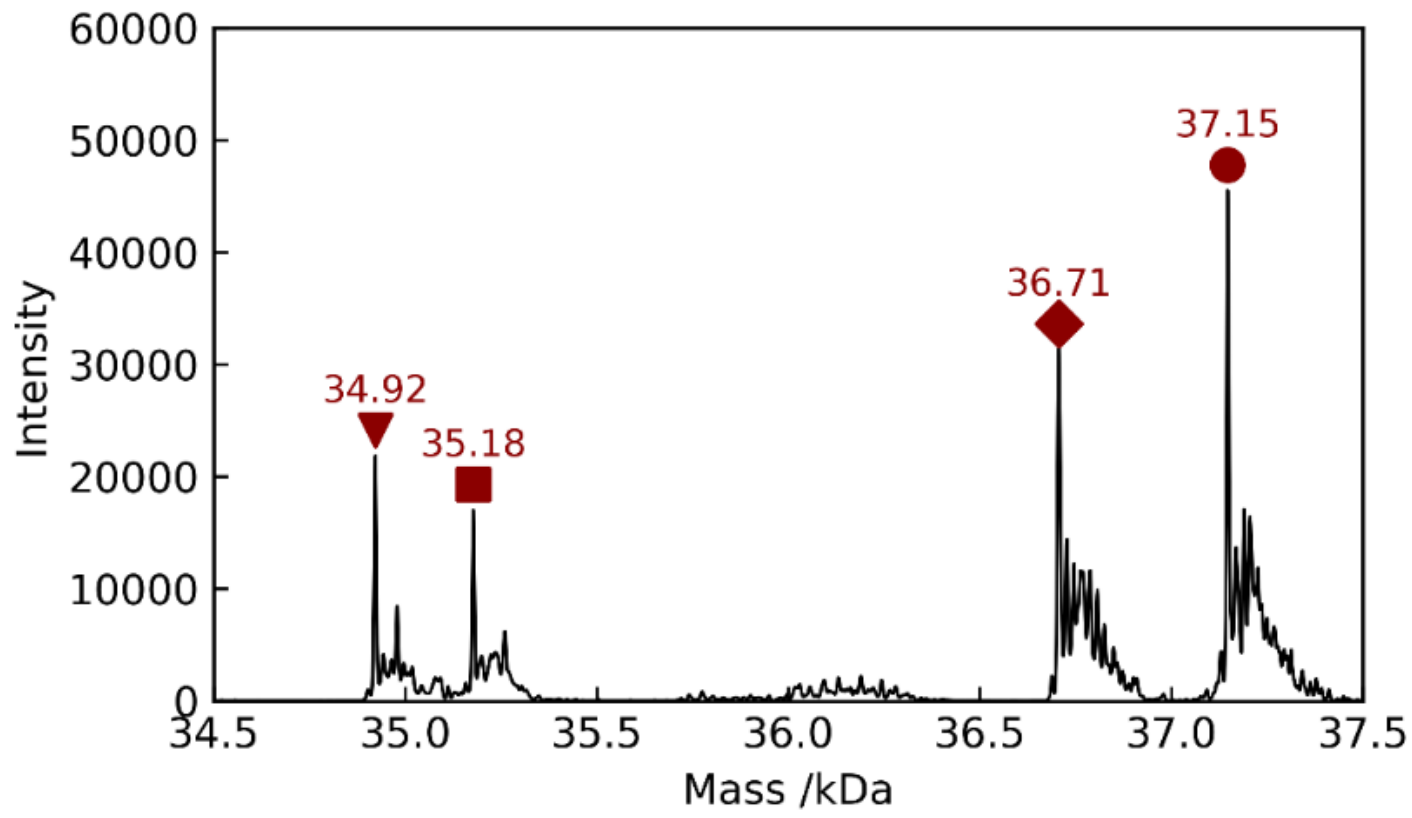

Figure S2: Mass spectrometry of the isolated, assembled SC003-mi3 construct reveals two discrete species. *The higher-mass variants are the dominant forms are consistent with the full-length SC003-mi3 subunit. The smaller population separated by ~2 kDa could result from C-terminal truncation. Mass*

spectrometric analysis was carried out on an Agilent 1290 LC system coupled to an Agilent 6530 QTOF mass spectrometer (Agilent Technologies) using a reverse-phase setup. Samples (20 µL of a 1 µg/mL solution) were injected onto a 2.1 × 150 mm Zorbax 300SB-C3 column (5 µm particle size) maintained at ambient temperature. Mobile phases consisted of chromatography-grade water (A) and acetonitrile (B) (Optima LC/MS grade, Fisher Chemical), each supplemented with 0.1% formic acid (Optima LC/MS grade, Fisher Chemical). The chromatographic gradient was programmed as follows (time in minutes/A/B): 0.00/90/10, 0.34/20/80, 0.36/5/95, 0.77/90/10, and 0.90/90/10, with a constant flow rate of 1.0 mL/min.Electrospray ionization was performed at 4 kV with the source temperature set to 350 °C. Nebulizer gas pressure was maintained at 60 psi, and drying gas flow was set to 12 L/min. Data were acquired in positive-ion full-scan MS mode over an m/z range of 100–3000, using a fragmentor voltage of 250 V. To minimize carryover, a blank injection consisting of running buffer was included between samples. Mass spectra were subsequently averaged and deconvoluted using Agilent MassHunter Qualitative Analysis software (version B.07.0; Agilent Technologies).

8. Line 98-100, there is something confusing about how this sentence is worded. We have tried to clarify this sentence.

Line 113: "Plotting the mass distribution on a logarithmic scale (Fig. 1b, middle) **shows the extremely low abundance of intermediates in the 150-500 kDa mass range**, **as well as particles** near the measured mass of complete mi3-VLPs (**2030 ± 10 kDa, see Supplementary note 1 and Fig. S2b**)."

9. Line 102 and Fig 1, I could not understand how the fully assembled capsid could be 15% when there are just 4 blips detected, but then I realized that each blip accounts for 60 monomers. One way the authors could help the reader to perceive this is to add the mass integration of these peaks (similar to how integrals are presented in an NMR trace) in the bottom traces of Fig. 1b (where an integral over the 4 blips would be shown to have a comparable value to, say, the integral over the monomer peak). Also, the concentrations (58 nM/350 nM) in Fig. 1b should be enlarged and moved away from the scale bar so that they are not confused with a scale bar length. In Fig. 1d, a legend should be included within the figure for easy reference. What do the shaded colors represent?

The reviewer raises an important point (see also comment 5 from reviewer 3). Attempts to include an integrated peak, however, made the figure too crowded and difficult to read. We therefore added an explanation in the text.

Line 118: "*Conversion **of particle abundances** into mass fractions (**Fig. 1b**, bottom, **where each species was weighted according to its oligomeric state**) shows that mi3-monomers are predominantly found in trimeric form (65 ± 4%) followed by fully assembled VLP (15 ± 4%)*"

We also modified Fig. 1 according to the above comments.

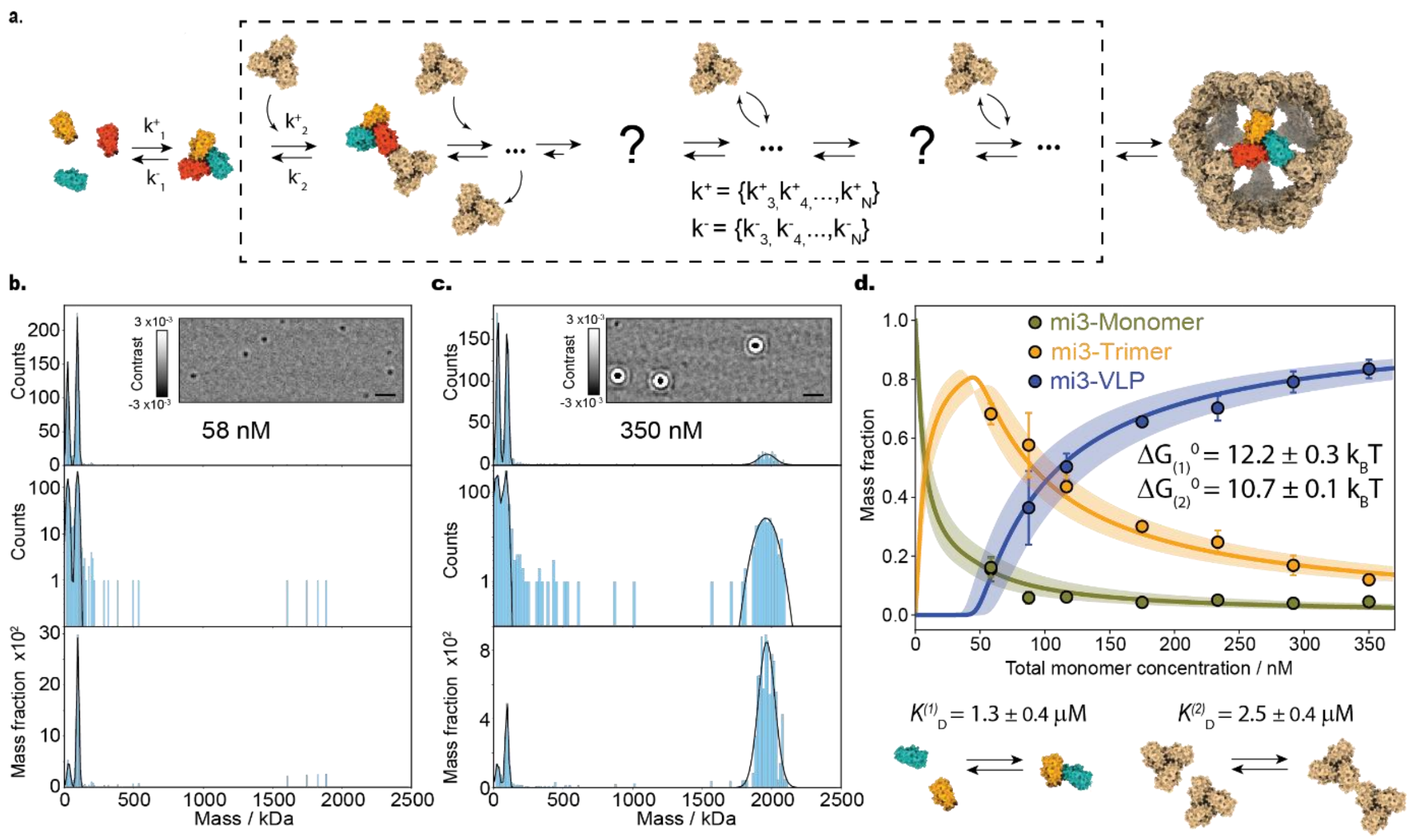

Finally, we have changed the legend of the figure to:

"…(d) Experimental mass fractions for the ***mi3-monomer*** *(green),* ***mi3-trimer*** *(orange) and assembled* ***mi3-VLP*** *(blue) as a function of the total protein concentration.* ***The expected linear relation between the total mi3-monomer concentration and the total detected mi3-monomers (Fig. S4) is maintained, suggesting similar detection probability for all oligomeric species.*** *Symbols and error bars correspond to the average and standard deviation of three technical repeats per concentration. Solid lines correspond to a global fit (Supplementary note 2.4) varying the standard free energy change for pairwise interactions between monomers (ΔG_((1))^∘) and trimers (ΔG_((2))^∘),* ***shaded colours and fitting errors correspond to 95% confidence interval of the fit.****"*

*10. Line 107, can these equilibrium constants be compared to literature values?

We could not find any experimental evidence for the pairwise interaction strength for this system. The original publication (10.1038/nature18010) introducing the I3-01 particle, shows that assembly at 8 µM subunit concentration at 1 M Guanidinium thiocyanate produces the assembled particle without indication of free subunits. While the experiment is not identical, it is consistent with a pseudo-critical concentration on the order of tens of nM. See also response to comment 9 of reviewer 3 for comparison with native capsids.

11. Line 127-131, as it is worded, this sentence implies that the increase in effective concentration derives from the constrained rotation, but it actually comes from the reduction of dimensionality. Also, the authors say "mi3-subunits do not interact with the SLB in the absence of a tag" but do not present evidence. Looking through the SI, perhaps Fig. S7 should be referred to here.

The bilayer causes two key changes compared to solution: 1. The increase in local concentration as a result of confining particles in 2D increasing the encounter probability/rate and thus effective concentration. 2. Alignment of the tethered protein in an orientation that favours binding and reducing rotational degrees of freedom and thereby changing the standard free energy of the interaction.

We refined the relevant sentence in the manuscript to:

Line 152: "*In this 2D configuration, rotational degrees of freedom are constrained, pre-aligning trimers for oligomerisation, increasing the interaction free energy gain*[58,59]. ***Additionally, surface confinement results in an increased effective concentration and therefore promotes assembly (Fig. 2a).***"

We also added a reference to Fig S7 that shows experimentally that we do not detect particles diffusing on the surface of the SLB when subunits are not functionalised with a histidine tag.

12. Line 139, as I understand, there is precedent for the longevity of the pentamer of trimers, as in Ref 51. This should be acknowledged.

We agree with the reviewer and added the relevant citation.

Line 166: "*We observe the expected exponential decay in higher oligomer abundance with the exception of the pentamer of trimers, which is stabilised by the additional free energy associated with ring formation,* ***as observed also for T=1 icosahedral capsids***[62]."

13. Line 235-237, I don't understand this argument about supersaturation. Supersaturation is not mentioned in Note 8.

Thank you, we have corrected the sentence to be more precise and in line with the SI notes.

Line 256: "*In this coupled configuration,* ***the chemical potential difference*** *(****Extended Data Fig. 3 and Supplementary note 5****) between* ***the*** *surface pentameric ring and* ***the*** *complete VLPs is constant, because the bulk concentration is effectively unchanged.*"

14. Fig. 4, the authors should include the number of individual trajectories to produce the panels in Figures b and c.

We added the total number of detected molecular trajectories to the figure caption.

Figure caption 3: " *(b)...Vertical lines correspond to the masses of closed-structure intermediates. Mass broadening, owing to particle size, for masses >1750 kDa prevents resolving individual molecular states at the ensemble level.* ***Mass histograms arise from 507, 850, 823, 1111 molecular trajectories that were further segmented, weighted by their observation time, and normalised by the total observation time per movie (Methods).*** *(c) Time-averaged mass distribution (between 4 and 15 minutes) and comparison with the calculated grand-canonical free energy landscape using interaction parameters from* ***Fig. 1****. Solid red lines: Topologically closed structures based on PDB ID: 7b3y. Dashed lines: Unstable configurations.* ***The structures presented for the 12-mer and 14-mer illustrate the corresponding topological state. Different specific molecular arrangements (with identical topology) are possible, due to their overall similar mass****.* ***The averaged mass distribution includes 15 different movies that include total of 19785 trajectories.***"

*15. Line 298 and surrounding area, the kinetic analysis in Figure 5 is exquisite, but I think it could be used in a somewhat richer manner. If one takes the dwell times in Fig. 5c, and simulates the equilibrium populations, do you get the relative populations in Fig. 4c? And if not why?

*17. Also, the authors should show (perhaps in SI), a histogram of dwell times for each stable intermediate (ie, the entries on the x axis in Fig. 5c). The average dwell time only gives the rate for a monoexponential decay process. Eyeballing the density of circles, it looks like some of the kinetics here may be non-monoexponentially distributed, but it is of course hard to see. If the decay kinetics

are non-monoexponential, then it would not be terribly surprising, since they know that each irreversible event includes multiple reversible intermediates. Still, I think the authors may have some additional information they can extract about those hard-to-see intermediates (for example, a minimum “waiting time” before the irreversible step can be completed. I appreciate that the dwell times for the pentamer of trimers is skewed because of the 20s “dead time” of the experiment, but it looks particularly non-monoexponentially distributed.

We have combined our response to these two comments/suggestions. Following the reviewer comments, we have added Extended Data Fig. 6.

Here we show:

1. The distribution of measured dwell times per state and how well they correspond to a monoexponential distribution. Originally, we decided to report only the average value, because we do not think we have enough statistics to resolve the number of decay timescales. Following the reviewer comments, we re-examined our single molecule traces and managed to extend the pool using incomplete trajectories that still provide events that report transitions between specific states (see new Fig 4 and S19-20). We now added the full distributions in the form of bar plots (Extended Fig. 7a). We still think that the limited statistics does not allow a quantitative statement regarding the number of components. For qualitative examination we added the expected monoexponential distribution based on the average value. It is important to note, this is not a fit to the distribution, only a calculation based on the average. While there could be multiple timescales coming from the requirement of 3 binding events that makes the overall irreversible step, we think that due to the fact that the pairwise interactions are very fast (diffusion limited) compared to the time to stochastically selecting the correct ring configuration, we cannot really see this timescale. We also added an additional figure for the reviewer that shows that the new trajectories added (7 trajectories) following the same behaviour of the previous data set.

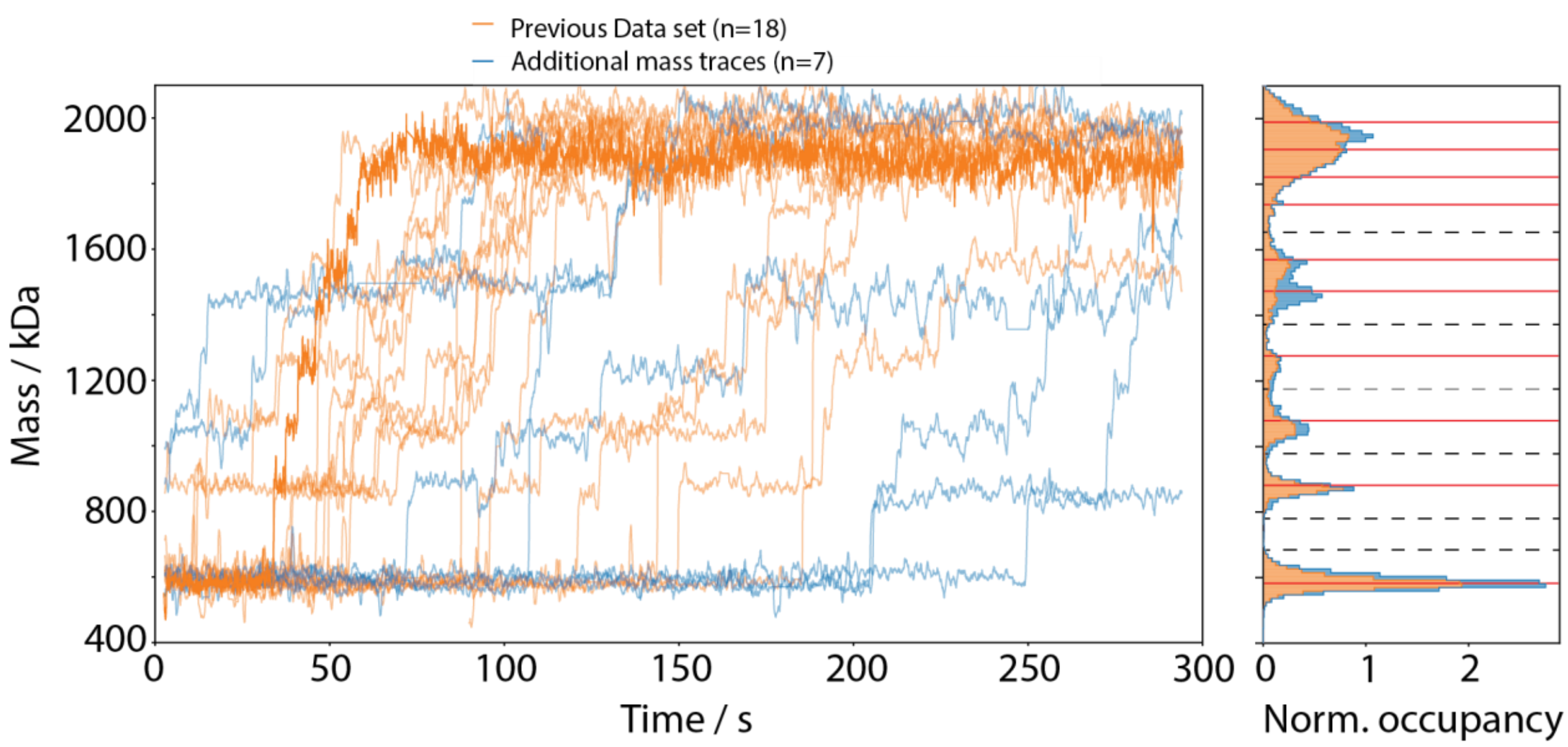

2. We agree with the reviewer comment, and simulated the expected relative occupancies. We would like to emphasise that the measured distribution on Figure 3c is not the equilibrium state. The equilibrium state is shown in Fig 1 and Extended Data Fig. 4a (time > 25min), where only a complete VLP is expected. Figure 3c shows the relative occupancies at timepoints where the intermediate occupancies are maximal at a solution concentration of 44 nM (4 min < t < 15 min). Therefore, to compare the dwell times with the population-level data, we need to compare the evolution of the distribution at the same solution subunit concentration. Figure 4c presents the assembly population-level kinetics at 44 nM of subunits in solution, the single complex assembly was done at 88-100 nM of subunits in solution, similar to the experiments presented in Extended data Fig. 4a and Figs. S12-

14. We therefore plot the simulated distribution and the population-level time varying distribution performed at similar conditions as the single complex experiment side by side. It is only informative to compare the first minutes of the reaction as the bulk assembly converges to the complete particle after about 5 min. Our simulation (based on the measured transitions times) and data show very good correlation between expected and measured distributions. Importantly, both the presented results here, as well as Fig. 4c and Extended Data Fig. 5 show that the intermediate occupancy (8-mer to 14/15-mer) during assembly is relatively flat, in very good agreement with our measured dwell time that show similar transition rates between the intermediate states.

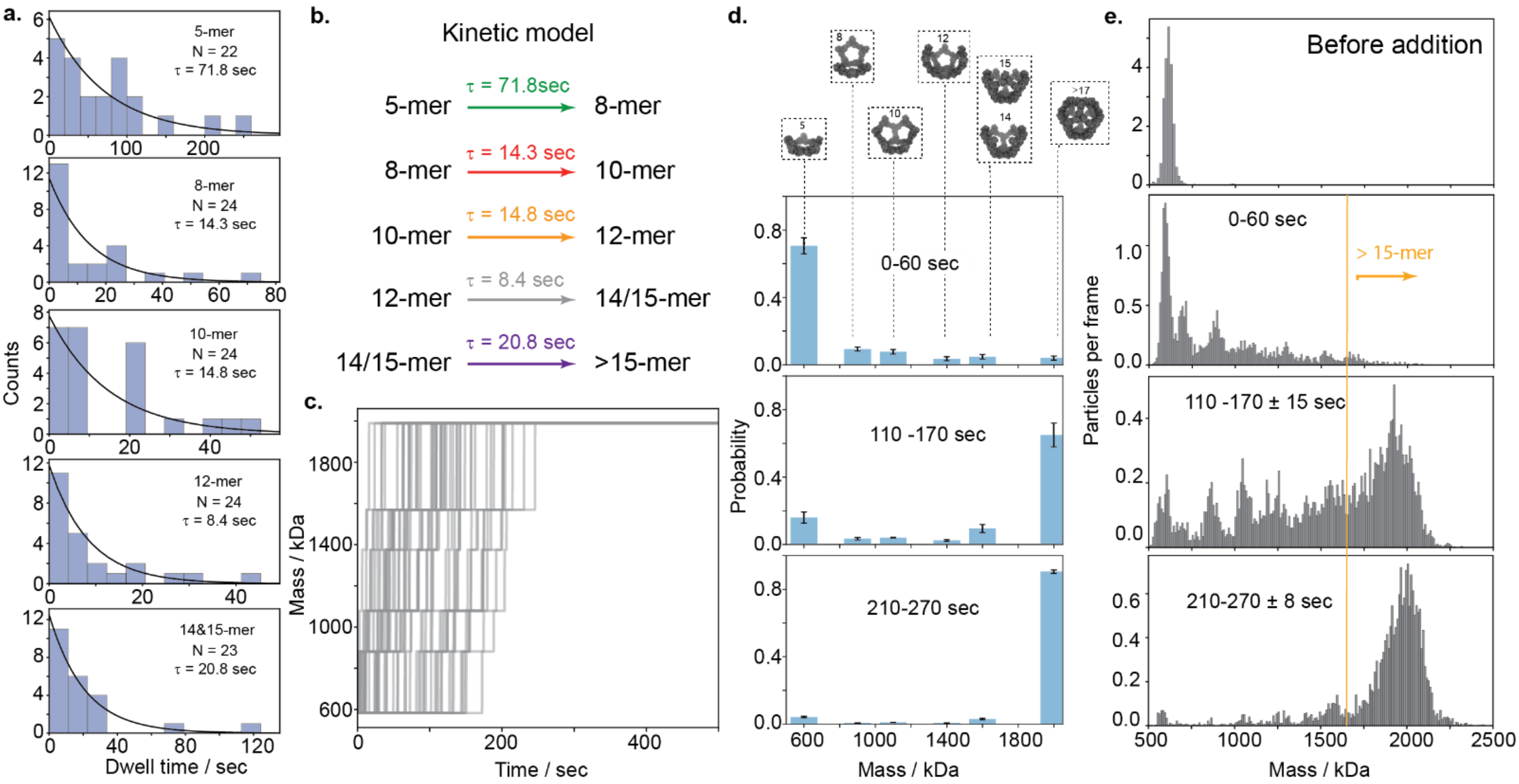

Extended Data Figure 7: Single-complex transitions dwell times. (a) Measured state-dependent dwell time distribution extracted from the assembly traces of individual mi3-VLPs. For each state the number of trajectories and the averaged dwell time are presented. The black curve corresponds to the expected exponential distribution given the calculated average. (b) The six state model and kinetic Monte Carlo simulation used to simulate relative occupancies of the different assembly states as a function of time, given the measured averaged transitions time. (c) An example of simulation results, showing the stochastic propagation of states for 50 tested trajectories. (d) The expected probability to sample each state during 60 sec intervals at different times during the process. Probabilities were calculated based on 500 traces (10 independent simulations rounds, containing 50 trajectories each). (e) Assembly reaction on continuous supported lipid bilayers performed at 88 nM subunits concentration in solution (see Fig. 4 for experimental description and Fig. S10-12 for the complete time trace at 88 nM solution concentration). Histograms represent averaged distributions over three independent measurements at similar time points following the addition of solution subunits.

3. For completeness, we also plotted the expected deviation of our results as a function of the dwell time owing to the limits of the measurement time (~20 sec – 300 sec). It shows that for the faster dwell times (~10-20 sec) the effect is minimal. For the longer dwell time (5-mer lifetime), we expect some deviation, therefore we stated that the dwell time of the 5-mer is probably a lower limit. We added Fig. S21 to explain this and to support the claim that we measure a lower bound.

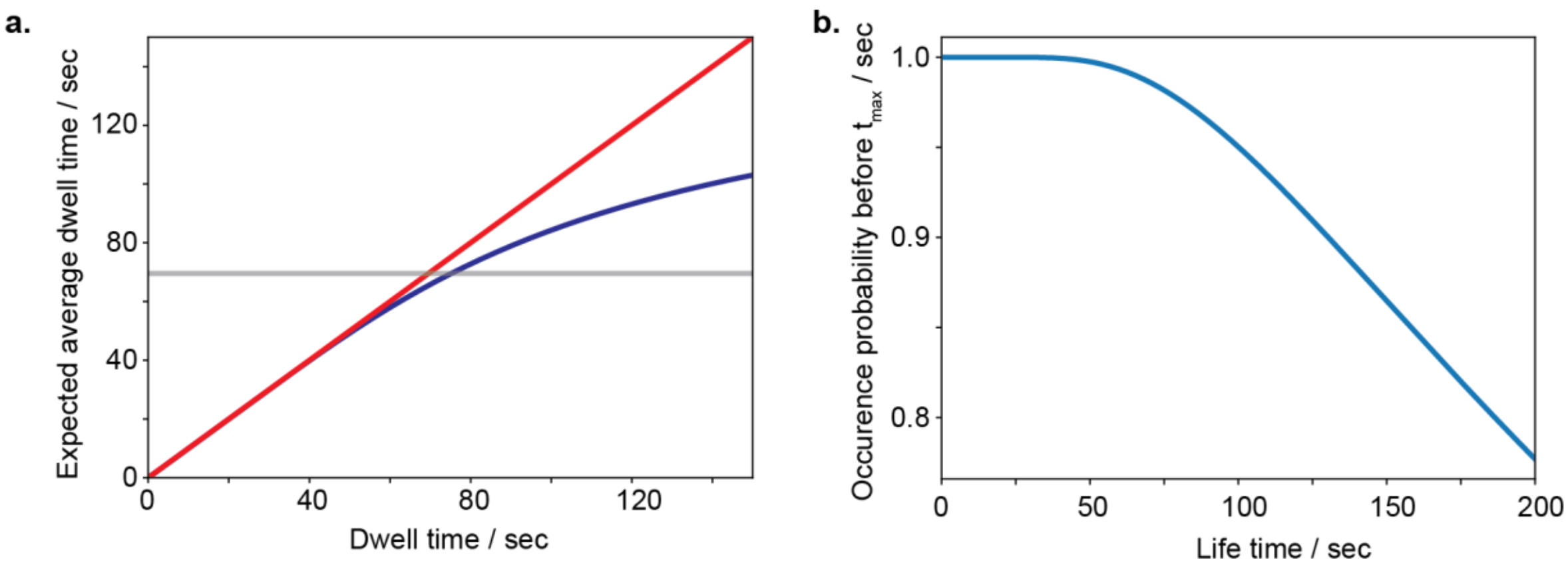

Figure S19: The measured averaged dwell time of the pentamer represents a lower bound. *The experimentally measured average dwell time of the pentameric ring represents a lower bound on the true lifetime because the observation window is finite (20–300 s after addition of mi3 subunits) (a) Assuming single-exponential kinetics, the initial 20 s delay does not affect the measured dwell time because only particles that remained in the pentameric state during this period were included in the analysis. However, transitions occurring after the end of the observation window are not observed. The average observed dwell time within this finite observation window is therefore given by,*

$$< \boldsymbol{\tau_{observed}} > = \frac{\int_0^{t_{max}} \frac{t}{\tau} exp\left(-\frac{t}{\tau}\right) dt}{\int_0^{t_{max}} \frac{1}{\tau} exp\left(-\frac{t}{\tau}\right) dt}.$$

where $\boldsymbol{t_{max}}$ *correspond to the beginning and end of the observation window, respectively.*
The difference between the true dwell time, $\boldsymbol{\tau}$*, and the theoretically observed* $\boldsymbol{\tau_{observed}}$ *therefore becomes significant for dwell times approaching* $\boldsymbol{t_{max}}$*. The grey horizontal line indicates the experimentally measured pentamer dwell time, demonstrating that the measured value represents a lower estimate of the true lifetime. (b) We can also estimate the upper bound by calculating the probability of observing a transition from the pentameric ring to a higher-order intermediate during the measurement window as a function of the dwell time. Because approximately 90% of pentameric rings were observed to grow beyond the pentameric state during the experiment, the corresponding analysis places an upper bound of approximately 125 sec on the pentamer dwell time at a mi3-monomer concentration of 88–100 nM.*

Changes to the text starting from line 334:

"*When evaluating the lifetimes associated with different intermediates (**Figs. S19-20**), the pentameric ring exhibited the longest dwell time, while other species showed broadly similar dwell times (**Fig. 4c** and **Extended Data Fig. 7a**). This is readily explained from a molecular perspective: Transitioning from the pentagonal ring to the next stable state (8-mer) requires binding of three weakly interacting subunits to form an additional topological face before any of them dissociates. The subunit concentration in solution determines the collision frequency, and therefore the probability that three subunits bind in the correct configuration prior to unbinding. By contrast, for subsequent transitions up to the 14-mer, closing each new face requires only two additional subunits, leading to shorter transition times. **Using the measured dwell times (Fig. 4c), we simulated the expected time-dependent oligomeric distribution of the closed configurations at a mi3-monomer concentration of 88-100 nM. The resulting population evolution is in good agreement with the independently measured population-level distribution acquired at comparable concentration (Extended Data Fig. 7b-c), showing a direct connection between the stochastic dynamics of individual assembly trajectories and the behaviour of the ensemble. We note that the reported pentamer dwell time may be affected by the ~20 s gap between subunit addition and the start of data acquisition as well as the maximum observation time of 300 sec (Fig. S22)**. Because the 14 and 15-mers are both closed structures, we report the cumulative dwell time (top of **Fig, 4c**). "*

18. Figure 6, I found part b) to be difficult to parse because the x and y axes are different between the three panels.

Thank you, we have modified the Figure to include a scale bar with uniform time and mass units, this is now changed to Extended Data Fig. 8.

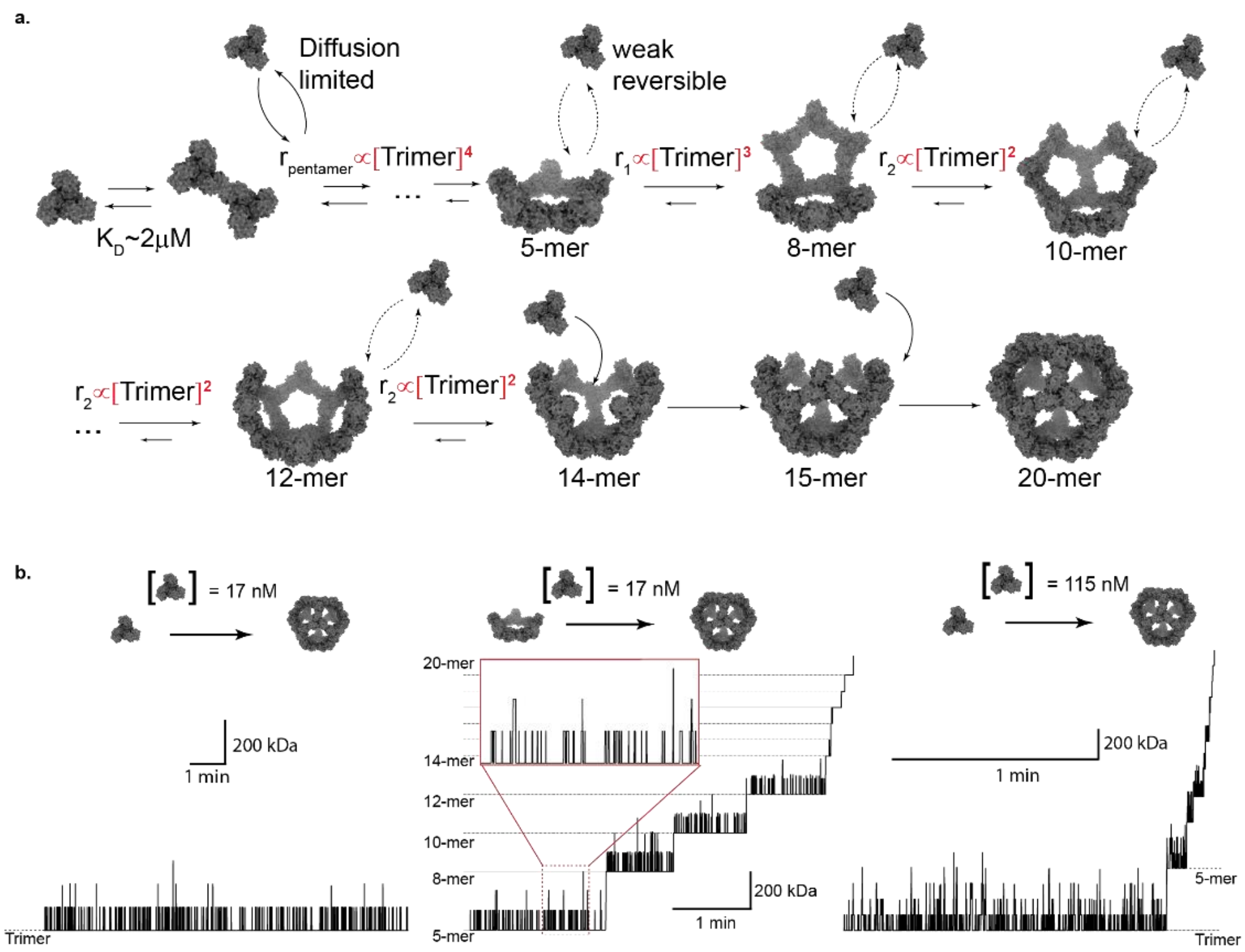

19. Line 348-349, I understand that it is expected that the reaction rates scale in this manner, but I don't believe the authors have measured this. If so, this should be presented as an "expected" dependence, rather than something that has been confirmed.

We agree with the reviewer comment regarding the rates and changed the text accordingly.

Line 395: "***Our measurements (Figs. 2) suggest that the interactions are independent and therefore we expect that the*** *corresponding reaction rates thus scale with the fourth, third and second powers of the trimer concentration, respectively*"

20. SI, Line 739, typo, "of" vs "if"

Thank you, fixed.

Referee #2:

Asor et al. report an elegant and seminal set of experiments to monitor assembly of virus-like particles. This has been a dream experiment of many scientists for decades. Using mass photometry, they are able to monitor and quantify each step, i.e. building block addition, especially neat is the assembly in SLB traps. The manuscript is extremely well written and data clearly presented. The conclusions are fully supported by the data. The results are relevant to a broad readership and impacts multiple fields. The approach will likely be applied to virus assembly or also other hetero-oligomeric machineries in the future. It is recommended to publish the manuscript in Nature after minor revisions addressing the following comments.

We thank the reviewer for their very positive reception of our work, highlighting the experimental achievements and the potential impact of our approach to multiple fields. Below, we address their comments.

1. line 107 - please use the same naming convention for the KDs as in the figures

Thank you, we have corrected the text.

2. supplement shows minor errors such as pixel x pixel is displayed in different layouts, suggested to unify the appearance, line 473 - "10 higher" is 10x higher meant? line 550 - should be Fig. S17b, and would benefit from an additional round of proof-reading

Thank you, we have corrected the errors in the SI text.

3. Fig S1c middle - the plot seems skewed with a dip in the middle of the x axis
-font size in main text figures is very small, sometimes too small

We have corrected the figure, and changed the text in the main figures to be larger where needed.

<u>Referee #3:</u>

1. This study investigates the assembly pathways of an engineered protein into a dodecahedral cage by directly observing the process at the single-particle level. Using mass photometry combined with single-molecule trapping, the authors track the growth of individual protein intermediates in real time and measure their molecular mass throughout the process.

We thank the reviewer for critically reading our paper. The main comments relate to the comparison to a previous paper by Garmann et al and the relevance of the results to the assembly of native capsids. Below we provide a point-by-point response to the comments and questions. Some comments share a common subject, in these cases we combined the responses to prevent repetition.

It may be helpful to provide a brief overarching response to the referee: We agree with all the points made. In an ideal world our work would be of one, or multiple, complex real viruses, not involve a surface, etc. However, as we hope the referee will agree, the kind of ***molecular-level dynamics*** we report in this study have not been reported to date by any method – they have been restricted to computer simulations. To achieve this, which is something that researchers have been attempting for decades, we had to use a simplified system and introduce a surface to capture intermediates that are otherwise invisible. Irrespective, the assembly rules we have learned will be applicable and very similar to some viruses, but will without doubt require additional regulation rules to describe others. What matters is not that we have solved the problem in a universal fashion: The key advance here is that these types of experiments can now be done, and that the information extracted is meaningful in the context of self-assembly – and complex biomolecular interaction dynamics more generally.

It is also important to note that we did not claim to have solved all aspects of virus assembly – our title and abstract are carefully written to describe what we have done and what we claim – and what we do not. We have, however, taken all the feedback from the referee to heart and adjusted the language to further strengthen this differentiation throughout the manuscript.

2. The claim that "no experimental approach has been successful in visualizing capsid assembly" and the limitations of other methods have been overcome by the present work is debatable. Garmann et al (2019, ref. 47) have used a similar approach to observe the kinetics of assembly of single virus capsids around viral RNA. Although that reference lacked the level of detail of this manuscript, it should be emphasized that ref. 47 used a real virus which had an icosahedral cage with 2649 possible intermediate species, while the current manuscript deals with a dodecahedral cage with 73 possible intermediate species.

We have clarified the relevant statement to be more accurate: "… *no experimental approach has been successful in visualizing* ***the molecular mechanism underlying capsid assembly***".

We will address previous studies, including the one by Garman et al. in the next, related comment.

3. The significance of the work for virus assembly is not clear. The construct is not a capsid. Instead, it is a highly engineered, wireframe protein dodecahedral cage that structurally resembles more to a clathrin cage than a virus. There are no dodecahedral viruses, to my knowledge so the title should be revisited. The building blocks of the dodecahedral cage are large and few and that is probably the main reason the same method has produced a clearer picture than Garmann et al in terms of the assembly pathway are the nature of intermediates. In addition, single molecule visualization with time course structural information has been demonstrated on surface supported, flat HIV coat protein assemblies (ref. 50, ACS Nano 2020, 14, 7, 8724–8734). Lower time resolution, but much higher spatial resolution than the current work. Granted, that system was also very engineered and a surface support was needed, not unlike the current work.

1. Significance Overall, we of course agree with the referee that in an ideal world we would have applied our method from day one to a real virus, ideally a complex one. However, one could also argue that this is the equivalent of questioning the importance of angstrom resolution cryo-EM 'because it was just done on apo-ferritin'. What we show is a multi-layered approach with unprecedented capabilities in the context of studying the molecular details of biological self-assembly – applying it to more complex systems is absolutely the next step. Importantly, as we show below, our results very much inform on fundamental set of assembly rules, including those governing natural viral capsids – even if not all!

2. We disagree with the claim that the only reason this manuscript yields a clearer picture than Garman et al is due to a choice of a simpler system:

a. We have not used the same method. Calling mass photometry the same method as iSCAT is equivalent to calling super-resolution imaging the same method as light microscopy or calling iSCAT the same as phase contrast microscopy. They share the same principle, but they provide very different information. They are thus not the same methods.

b. We have now added a more explicit version of the data originally shown in Fig. 2c. It shows that we can detect mass changes corresponding to individual 25–40 kDa protein subunits on the 100 ms temporal scale with 30x higher signal-to-noise ratio (resulting in an SNR of 3-10 for 25 kDa subunits) compared to the iSCAT work by Garman et al. This is a ~300-fold difference in combined temporal resolution and sensitivity, and solely responsible for the molecular level detail shown in this manuscript, which forms the entire basis of this study.

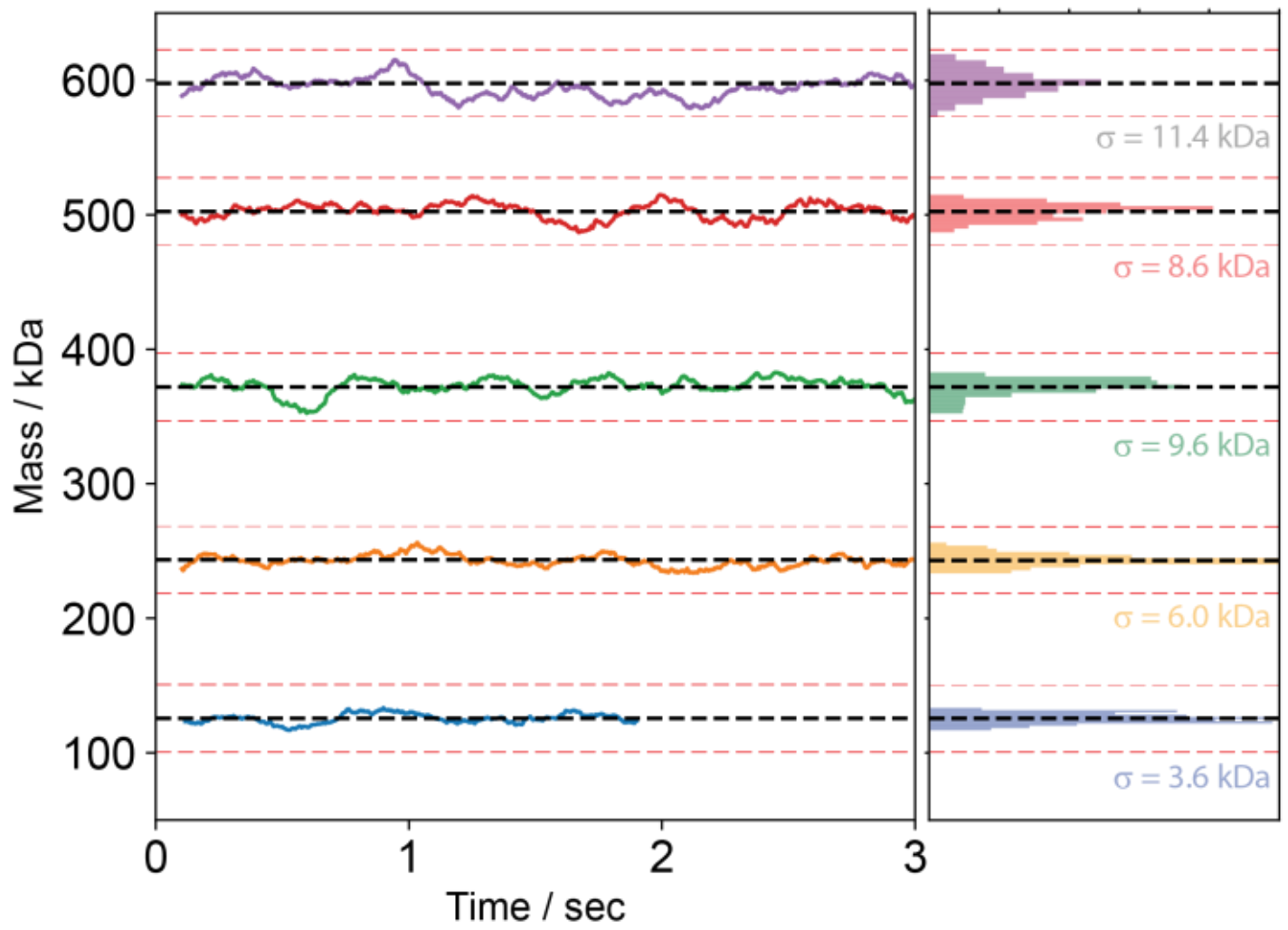

Figure S7: Mass traced on supported lipid bilayers of oligomeric species between 120 and 600 kDa. Mass traces were acquired at 270 Hz and are presented following further averaging with a final sampling rate of 10 Hz. Red horizontal dashed lines indicate a 25 kDa deviation around the average. The traces histograms and standard deviations are shown to the right.

3. Comparison with Valbuena et al. We are not sure spatial resolution is critical in the context of capsid assembly, especially for well-understood structures (of which there are many!). What matters is the ability to resolve the different assembly intermediates. No capsid assembly was observed in that manuscript, so we are not sure it is a valid comparison.

Nevertheless, we have made the following changes to the text to address the reviewer comment to avoid similar sentiments by a general reader:

Introduction, Line 78:

*“**In terms of monitoring capsid assembly, interferometric scattering microscopy has been used to monitor the formation of capsids on an RNA template[46], yielding information on nucleation and elongation at the single particle level. This study, however, did not possess the temporal resolution and sensitivity required to reveal molecular level details of the assembly process.**”*

We also added the above figure to the supplementary material and added a reference in the caption of figure 2.

Fig 2 caption:

*“(c) Representative mass traces of different oligomeric states, measured at the raw instrument frame rate (270 Hz), **and after 28 frame averaging (~10 Hz), showing the achievable mass resolution at ~100 ms integration time (Fig. S7)**”*

Line 104:

“*Although the geometry is dodecahedral rather than the triangulated icosahedral lattice found in **viruses**, **we expect that** the fundamental biophysical principles governing the assembly process **of empty capsids** are conserved. **The exact capsid and assembly mechanism**, **symmetry and local conformations, however, will** govern the specific pattern of locally stable intermediates (Fig. 1a), the degeneracy **values of** microstates, and **the exact dynamics of** how assembly pathways branch[33,40].*”

Line 76: “…***while atomic force spectroscopy, reveals structure and interactions at the protein subunit scale only in 2D, where full capsids cannot assemble[48,49]***”

To further address the reviewer's concerns, we have revised the relevant sections of the manuscript to better acknowledge previous work (See also response to comment 11) and clarify the rationale for using this model system (see also response to comment 4). We have also modified the statement in the Introduction to more precisely describe the missing capabilities of existing approaches.

4. The system under study is an engineered protein cage under surface-confined conditions, with no nucleic acid involvement. As such, additional controls and comparisons are needed to establish how well the results translate to natural virus assembly, particularly given differences in geometry, molecular interactions, and the absence of nucleic acids or other viral components. Several aspects of the experimental design, analysis, and interpretation limit the generalizability of the conclusions.

9. The authors extract key thermodynamic parameters (e.g., dissociation constants and pseudo-critical concentration), but these values are not directly compared to those reported for well-characterized viral systems such as HBV, MS2, CCMV or BMV or consistent with other, established ensemble-averaging methods.
As a result, it is unclear whether:
- The interaction strengths fall within physiologically-relevant ranges
- The observed assembly pathways are representative of viral capsids
- The inferred mechanisms are broadly applicable or system-specific
- The observed kinetics are realistic
These omissions weaken the claim that the study reveals general principles of virus assembly.

We combined Comments 4 and 9, as both concern the extent to which the mechanistic insights obtained from our engineered system are relevant to native virus assembly.

We will attempt to address the many implicit and explicit points raised by the referee:

1. Surface confinement. The system is not studied under surface-confined conditions. *Some* aspects of the work are under surface confined conditions, others are in free solution. The choice is made depending on what we are attempting to observe. Measurements are made under both conditions, and the surface is used because it is experimentally unavoidable if intermediates are to be observed. In fact, the mechanistic information that we extract proves exactly this point. Specifically, Figure 4 and Extended Figure 5 show directly that the distribution we observe on the SLB follows exactly the free energy landscape calculated for the solution. The key question really is: has there been a substantial advance in visualising, quantifying and thereby understanding capsid-like assembly? We strongly believe that this is the case here (even if imperfect!) *because of the molecular dynamics we observe, which is the main claim of the manuscript*.

2. Claim of generality. We do not believe that our paper makes such a claim. We could not find any such statements in the abstract, for example. What we do claim is that we observe molecular level detail of self-assembly, and that this observation enables us to understand fundamental self-assembly principles and how the system navigates the assembly space.

3. Translation to natural viruses. We agree - and do not claim otherwise - that our system is not a native capsid and lacks several features such as conformational regulation, and the specific geometries of some viral species (here the focus is assembly without a template). We have clarified this point early in the manuscript and explained the rationale behind it. Following the reviewer comments, we have also expanded the discussion section to better explain how the measured mechanistic findings translate to a native viral capsid assembly. We also agree that comparison with well-characterised viral systems is valuable and have expanded the discussion accordingly. However, direct quantitative comparison of thermodynamic parameters between different assembly systems is not straightforward and may also not be informative, because quantities measured in the bulk do not necessarily report on the same microscopic processes.

We have not and do not claim that the *specific* thermodynamic or kinetic numerical values measured here are directly transferable to all viral systems. Rather, we aim to measure and identify fundamental mechanistic physical principles that govern robust self-assembly across self-limiting systems, such as viral capsids and virus-like systems. In particular, our results provide a critical layer of information that was previously beyond experimental reach, and supports a framework in which: (i) Assembly is driven by weak and reversible pairwise interactions, allowing annealing and avoiding kinetic traps through nucleation-type assembly. (ii) Stability emerges from stochastic selection of a restricted set of highly coordinated, productive intermediates that guide assembly along preferred pathways and minimise kinetic trapping. (iii) Stochastic selection results in a separation of timescales between the first stable state and the rest of the assembly process, which is the origin of the nucleation timescale in this system. (iv) Revealing the connection between the stochasticity of single-complex dynamics, population level observation and the connection to the calculated free energy landscape.

Critically, our conclusions are consistent with both suggested mechanisms from computational and experimental studies of viral capsids – thus, insofar it is possible, the existing evidence supports the direct relevance of our results for native viruses. Early molecular dynamic simulations (https://doi.org/10.1103/PhysRevLett.101.186101) suggested that simple icosahedral capsid assembly proceeds through reversible association events and that only a small subset of strongly bonded intermediates contributes substantially to productive assembly. These arguments were also supported by kinetic simulations (see multiple works by Zlotnick et al) and more recent coarse-grained simulations (multiple works by Hagan et al). Similarly, recent multiscale simulations of HBV assembly identified weak reversible interactions combined with a limited number of stabilising configurations that control successful capsid formation despite the enormous number of possible assembly states. These assembly hubs are topologically closed and appear to be long-lived although the pairwise interactions are reversible (https://doi.org/10.1021/acsnano.2c02119, as well as https://doi.org/10.64898/2026.05.25.727729). Experimental observations are also broadly consistent with this picture. Ceres and Zlotnick (https://doi.org/10.1021/bi0261645) showed that **HBV** capsid assembly can be driven by relatively weak protein–protein interactions. Studies of **CCMV** (https://doi.org/10.1006/viro.2000.0619) suggested small oligomeric nuclei, including a closed pentamer of dimers, as productive assembly intermediate (or nucleus). Similar observations were reported for simple T=1 **MVM** capsids, comparable to our assembly system (https://doi.org/10.1021/jacs.6b07663) and **MS2** bacteriophage (https://doi.org/10.1002/rcm.4732). A widely accepted review (https://doi.org/10.1146/annurev-physchem-040214-121637) has highlighted the fact that for empty capsids the critical nucleus often corresponds to a small closed polygon that maximises the coordination number per subunit. Lastly, maximising the coordination number per subunit was also shown to be important for the assembly path of HBV (DOI: 10.1126/sciadv.abg0811). Taken together, thus, both in silico and experimental evidence is in close agreement with the observations reported here! However, as we are sure the referee agrees, this manuscript is not the place to provide a literature overview of this magnitude.

Finally, our results demonstrate that long-lived and effectively irreversible assembly states can emerge from the collective stabilisation provided by multiple independent interactions, even in the absence of an explicit conformational transition. While conformational regulation may alter the quantitative relationship between concentration and assembly kinetics, it does not alter the fundamental requirement for bond formation and stabilisation, and the emergence of a restricted set of productive assembly intermediates. More generally, we view the simplified nature of the system not as a limitation with respect to biological complexity, but instead as an experimental advantage that allows isolation of the fundamental physical interactions governing self-assembly. For instance, very recent studies using engineered particles have similarly demonstrated that simplified model systems can reproduce key features of capsid assembly (https://doi.org/10.1038/s41586-026-10554-z), including polymorphism and triangulation-number selection, while providing mechanistic insight that is difficult to obtain directly from native viruses.

We have revised the manuscript to make these considerations clearer and to emphasise that the significance of our findings lies in the experimental observation and quantification of the physical mechanisms of assembly revealed by the measurements, rather than in a one-to-one correspondence between the measured parameters and those of any viral capsid system, as well as addressing additional factors that will provide another layer of regulations in the case of native viruses.

Changes to the text:

End of the discussion:

"***Our work provides experimental access to fundamental assembly mechanisms of empty virus-like particles, showing how weak, reversible, multivalent interactions give rise to nucleation-like behaviour through a restricted set of highly coordinated, productive intermediates. Additional regulatory factors, including conformational regulation and shell elasticity, are nevertheless expected to further shape assembly pathways of native capsids and capsid-like structures. These have been proposed to play important roles in capsid polymorphism[66] and in affecting assembly outcomes in response to small-molecule assembly modulators[27] and will be subject of future work using our approach.*** *Beyond the implications for our understanding of the fundamental principles driving and controlling self-assembly, our approach represents a novel framework for studying biomolecular assembly and dynamics more broadly. Characterising pairwise interactions, quantifying equilibrium distributions and monitoring individual assembly events and dynamics is general: Here, it was applied to a simple, homo-oligomeric, analyte, but* ***owing to the universality of mass measurements, our approach can be applied to a broad range of multi-molecular systems,*** *opening up new avenues for studying biomolecular dynamics and mechanisms in the context of biological function and regulation.*"

Line 96 at the beginning of the results section:

"*To enable an analytical understanding of the stoichiometries and topologies involved in the assembly pathway of* ***empty VLPs****, we selected an engineered, self-assembling virus-like particle (mi3-VLP[55], see Methods), currently under evaluation as a multivalent vaccine platform[9,65-67]. The mi3-VLP was derived from i3-01[12,56] . The mi3-VLP consists of 60 identical mi3-monomers, hierarchically assembled into a dodecahedron from 20 homotrimeric building blocks (****mi3-trimers****), yielding a particle with 12 pentagonal faces, sharing molecular similarity with T=1icosahedral native viral capsid (Fig. 1a). Although the geometry is dodecahedral rather than the triangulated icosahedral lattice of native viral capsids,* ***we expect that*** *the fundamental biophysical principles governing the assembly process* ***of empty capsids*** *are conserved.* ***The exact virus and assembly mechanism****,* ***symmetry and local conformations, however, will*** *govern the specific pattern of locally stable intermediates (Fig. 1a), the degeneracy* ***values of*** *microstates, and* ***the exact dynamics of*** *how assembly pathways branch[33,40].*"

6. Reducing dimensionality from 3d to 2d allows mass photometry to access oligomerization dynamics, but also increases the artificial nature of the study.

We have covered this in point 1 in the previous comment.

5. The approach and the chosen system are perfectly matched to maximize the amount of information that could be extracted by using the mass photometry method. The quality of data is mostly high, with some exceptions where statistics are questionable. Specifically, Fig. 1 b: fully assembled VLPs representing 15 +/- 4% is a conclusion drawn from 1-2 counts. Describing the statistics as insufficient would be an understatement.

We are not sure what is meant by this comment, it seems only natural to study a system that can be studied and validated at the point in time where a new methodology is developed. We have already moved beyond the capabilities shown here – we believe this is completely normal.

The concern relating to the data: We believe that the above concern arises from considering a single data point in isolation rather than the complete dataset that was actually used for thermodynamic analysis. Importantly, neither the pairwise interaction energies nor any other thermodynamic parameters reported in the manuscript are inferred from individual measurements. *The analysis is based on a global fit to the entire titration series, comprising seven concentrations and three technical replicates per concentration.* The robustness of the extracted parameters therefore depends on the measured behaviour of the full dataset rather than on any individual point. As illustrated in Figure 1d, even substantial variation in the occupancy of the lowest-concentration sample results in only minor changes to the extracted interaction energies (approximately 0.1–0.3 $k_B T$). Furthermore, the reported quantity corresponds to mass-fraction occupancy, where each complete VLP contains many subunits and therefore contributes significantly to the overall population balance. As a result, even relatively rare complete assemblies provide meaningful thermodynamic information.

For these reasons, we do not believe that the thermodynamic analysis suffers from insufficient statistics. In Fig. 1, we choose to include the lowest and highest concentrations to give the reader a better qualitative understanding of the expected distributions along the titration curve. The lowest concentration, as expected, is close to the pseudo-critical concentration and therefore the number of complete (or close to complete) VLPs is low.

Nevertheless, to avoid confusion, we have revised the text to explicitly state that the low abundance of complete VLPs near the pseudo-critical concentration is expected.

Changes to the text, line 122:

*"**By globally fitting the law of mass action across multiple total protein concentrations, we can convert these relative abundances of the three dominant molecular states (mi3-monomer, mi3-trimer and VLP) into the affinities governing the assembly process (Fig. 1d and Supplementary note 2),**[36,52]"*

7. Mass photometry provides information on particle mass but not structure. Consequently, identical masses may correspond to distinct structural states and intermediate assignments rely on assumed geometries rather than direct observation. While the mi3-cage structure is known from prior work, the study does not include any structural validation (e.g., EM, AFM) of the assembled species under the experimental conditions. Perhaps, for mi3 subunits which are very rigid conformation is not supposed to change, but for viruses the ability to switch between conformers is a characteristic required by quasi-symmetry.

The reviewer is correct, in mass photometry we are almost exclusively sensitive to the total mass and not the structure. In figure 4-6 we provide structures that corresponds to each mass, but here we meant to address the topology, rather than the specific arrangement. For the 12-mer and the 14-mer there could be more than one configuration with exactly the same stoichiometry (but with identical topology). The degeneracy does not change our results or interpretation but it is important to state it in the text. We therefore change the text accordingly. Figure 3 caption:

*"…(c) Time-averaged mass distribution (between 4 and 15 minutes) and comparison with the calculated grand-canonical free energy landscape using interaction parameters from Fig. 1. Solid red lines: Topologically closed structures based on PDB ID: 7b3y. Dashed lines: Unstable configurations. **The structures presented for the 12-mer and 14-mer represent the topological***

***state. Different specific molecular arrangements (with identical topology) are possible, due to their overall similar mass**…"*

Showing the structure of the VLPs will contribute little to the conclusion of the paper, since the structure was well characterised in previous studies (https://doi.org/10.1038/nature18010, https://doi.org/10.1021/nl062449h). Of course, this is <u>also</u> true for the majority of well-studied viruses: the capsid structure is well characterised, it is the assembly path that is not well understood. We agree with the reviewer that to study polymorphism and assembly conditions that lead to malformed or polydisperse structures that deviate from the known structure, such a combination is required. This is exactly the kind of work we are currently undertaking using MP in combination with computational and structural approaches to understand the basis of polymorphism (https://www.nature.com/articles/s41594-025-01490-z)!

8. The analysis assumes that landing events faithfully reflect solution-phase concentrations. Is there an experimental basis for this assumption? Surface-based detection may introduce biases due to differential surface affinity of species and possible distortion upon surface interaction. These factors could affect the relative abundance of oligomeric states, particularly for weak or transient intermediates. The manuscript does not explicitly address or control for such biases.

Mass photometry has now been used for about a decade and to date in thousands of experiments and protein systems there has been no evidence for significant surface-induced bias, especially not in homogeneous solutions such as those used here. There is also no physical basis for such a difference between weak and transient intermediates and stable ones, because it would require very different surface interaction properties for trimer and dimers of trimers, for example. We have investigated this in detail in our original publication (DOI: 10.1126/science.aar5839, SIFig. 4).

To nevertheless address the reviewer's concern, we have added a concentration-linearity analysis to the Supplementary Information and clarified these points in the manuscript.

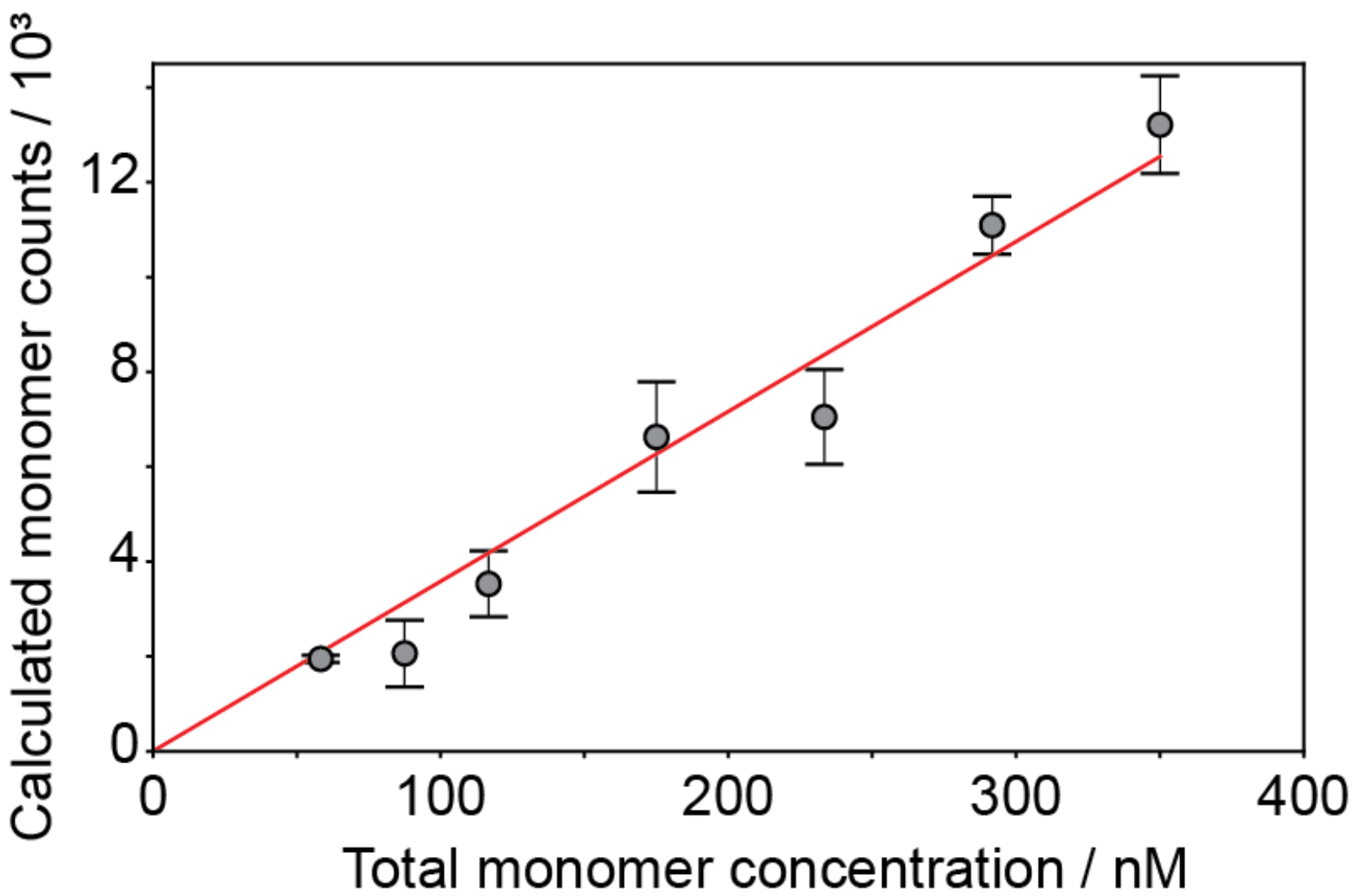

Figure S4: Calculated total mi3-monomer detected during 60 sec acquisition MP movie as a function of total mi3-monomer concentration. The calculated values were extracted by fitting a gaussian peak for the mi3-monomers, trimers and complete capsids. The area under each peak corresponds to the number of detected particles. The total counts of each oligomeric state were multiplied by the aggregation number (1, 3 or 60) and the total weighted counts were summed, and presented as a function of the incubated concentration of the samples.

Changes in the text, Fig. 1d caption:

"…d) Experimental mass fractions for the mi3-monomer (green), mi3-trimer (orange) and assembled VLP (blue) as a function of the total protein concentration. ***The expected linear relation between the total mi3-monomer concentration and the total detected mi3-monomers (Fig. S4) is maintained, suggesting similar detection probability for all oligomeric species****."*

10. Generalizing the pairwise-additive interaction assumption might be a stretch. Is there support for this assumption? There have been suggestions in the literature that this is not the case for viruses.

We agree with the reviewer that strictly pairwise-additive interactions are not a generalised description of <u>all</u> viral capsid assembly processes. We are not aware of having generalised this claim, but state it as a measurable of our system. Several viral systems have been suggested to exhibit additional layers of regulation, including conformational switching, quasi-equivalent subunit environments, RNA-mediated effects, and other state-dependent interactions that can introduce non-additive contributions to the assembly free energy landscape. In particular, viruses that assemble into quasi-equivalent structures often require proteins to adopt multiple conformational states, and the energetic cost of these conformational transitions may contribute to the overall assembly thermodynamics.

Our objective is not to establish pairwise additivity as a universal feature of virus assembly. Rather, our results demonstrate that pairwise-additive interactions are sufficient to generate many of the key features commonly associated with capsid assembly, including nucleation, stochastic pathway selection, the emergence of highly stabilised intermediates, and effectively irreversible assembly despite individually reversible bonds. In this context, the significance of our findings is not that all viral capsids are governed exclusively by pairwise-additive interactions, but rather that pairwise-additive interactions alone are sufficient to generate the core assembly behaviour observed in many capsid systems. More complex viral architectures may incorporate additional regulatory mechanisms on top of this foundation, but the physical principles identified here remain relevant to understanding how local interactions give rise to robust assembly pathways. We have modified the discussion accordingly to emphasis additional contributions for the regularisation of the assembly process that were previously suggested. See response to comment 9.

11. The discussion of prior literature occasionally emphasizes limitations of previous approaches without highlighting their contributions. A more balanced framing would improve context.

Thank you for the comment, we have revised the Introduction to more clearly acknowledge the contributions of earlier experimental approaches while also discussing the specific limitations that motivated the development of the present method.

Starting from line 76:

"Second, the involvement of 10s to 1000s of subunits results in an exponentially increasing number of molecular assembly paths and a high degree of heterogeneity in solution[39,40]. As a result, ensemble-based approaches have struggled with this heterogeneity, relying on complex analysis or modelling[33,34,39,41-43]. ***Single-molecule approaches have been applied to overcome such heterogeneity: Charge detection mass spectrometry (CDMS) and resistive-pulse sensing provide ensemble-averaged snapshot observations of a subset of stable intermediates, but struggle with the temporal resolution required to capture dynamics and, in the case of CDMS, does not operate in solution[42-44] . Single-molecule fluorescence yields dynamics but cannot quantify the degree of oligomerisation[45,46,47], while atomic force spectroscopy, reveals structure and interactions at the protein subunit scale in 2D, where full capsids cannot assemble[48,49]. In terms of monitoring capsid assembly, interferometric scattering microscopy has been used to monitor the formation of capsids on an RNA template[46],***

yielding information on nucleation and elongation at the single particle level. This study, however, did not possess the temporal resolution and sensitivity required to reveal the molecular-level detail of the assembly process. As a result, despite decades of dedicated study, no experimental approach has been successful in visualising the molecular mechanism underlying capsid assembly[13]. "

12. The introduction is prone to generalizations, e.g. L 53 on assembly efficiency: Depending on what is understood by "efficient", this generalization might be a stretch. There are several well-studied spherical viruses with symmetrical capsids for which assembly also produces non-infectious particles including sub-viral particles a defective particle. Examples: HBV, Influenza, Paramyxoviruses (Senday, Measles), and a few retroviruses. Judging from the reference list, it would seem that HBV is the canonical icosahedral virus. However, a few of the features of assembly that are mentioned as general, have been identified much earlier in other systems, e.g. Prevelige, P. E., Jr., Thomas, D., & King, J. (1993). Nucleation and growth in bacteriophage P22 assembly. Biophysical Journal.

This section in the introduction is important for contextualising our work, we therefore thank the reviewer for their comment. The word efficient is indeed too general in the context of the listed sentence and we have therefore modified this part of the introduction.

Changes to the text, line 56:

"*This process showcasing self-assembly dynamics finely tuned to distinct phases of the viral life cycle,* ***yet capable of proceeding spontaneously with high yield, enabling its recapitulation in relatively simple in-vitro experimental systems across multiple viruses[13]***"

We agree that there are multiple viral systems that show similar assembly principles, which is why we believe that understanding the process on the molecular level is so exciting. Here, the references focus on HBV capsid assembly because in terms of the assembly of empty capsids (similar to the focus of our work) the thermodynamics of this virus was highly studied using state of the art analytical and biophysical techniques as well as theory and simulations. These kinetic and thermodynamic results are central to the field of capsid assembly. We also agree with the reviewer that the suggested paper should have been included in this section as it shows/identifies the nucleation and growth mechanism of capsid assembly. We added a reference in the introduction and discussion (see also answer to the next comment).

13. A generalization of the accepted mechanism for empty capsid formation is on L 63: "key intermediates are short-lived and of very low abundance". It should be clear, this is not true for viruses: See the tobacco mosaic virus - classic textbook example, 34-subunit coat protein disks (the key intermediate) form first, then viral RNA binds to a specific site on a disk, and the ring rearranges into a helical intermediate. Several experimental and theoretical works of in vitro co-assembly involving the encapsulation of cargo (cognate or not) have shown that there are alternate pathways to the "low-abundance key intermediates" one. References to these works are conspicuously absent. There are some very strong claims in conclusion - if there are no limits why not study a real virus?

We thank the reviewer for this thoughtful comment and for highlighting the important distinction between spherical capsid assembly and other classes of viral assembly, such as the assembly of helical viruses exemplified by tobacco mosaic virus. We agree that our original wording may appear too general. Our intention was specifically to describe the assembly of simple spherical capsids not viruses in general. We have therefore revised the text to clarify this point. The revised sentence now reads:

"The fundamental challenge to visualise the molecular details of ***simple spherical*** *capsid assembly comes down to two factors. First, the* ***self-limiting*** *free energy landscape* ***results in a well-defined, finite and closed complex, and*** *involves weak and reversible pairwise interactions[31-34], meaning that assembly is stochastic, while key intermediates are* ***typically short-lived and of low abundance[31-35]****."*

For spherical capsids, this picture is well supported by both theory and experiment. Thermodynamic and kinetic models of capsid assembly predict a nucleation-and-growth mechanism in which the equilibrium population is dominated by free subunits and complete capsids, while intermediate states occupy higher free-energy regions of the landscape and therefore remain transient and sparsely populated (https://doi.org/10.1146/annurev-physchem-040214-121637, DOI: 10.1016/j.bpj.2009.11.023, doi: 10.1529/biophysj.105.072975, https://doi.org/10.1016/S0006-3495(02)75245-4, https://doi.org/10.1103/RevModPhys.93.025008). Experimental studies on HBV and CCMV assembly have likewise reported low-abundance intermediates consistent with these predictions *(*https://doi.org/10.1021/acsnano.9b00648 , https://doi.org/10.1021/jacs.0c01092(HBV), https://doi.org/10.1039/c6sm01060a (CCMV)*).*

This idea is also suggested by the early paper of capsid assembly that the reviewer referred to "Prevelige, P. E., Jr., Thomas, D., & King, J. (1993). Nucleation and growth in bacteriophage P22 assembly. Biophysical Journal", as a consequence of the suggested nucleation and growth model. As pointed by the reviewer, this fundamental work was indeed missing in our introduction and discussion, and we now include it.

Our motivation for focusing on the assembly of an empty, homo-oligomeric shell is that the protein-protein assembly reaction represents a fundamental component of capsid formation whose molecular details remain incompletely understood due to the difficulty of directly observing assembly intermediates. We view the present work as establishing an experimental framework for resolving these intermediates at molecular-state resolution. Future applications of the method to native viral capsids and to nucleic-acid-directed assembly systems will allow the respective contributions of protein-protein as well as protein-cargo interactions to be investigated directly.

We also agree with the reviewer that assembly around a nucleic acid template can proceed through additional mechanism that is not captured exactly by the description of empty capsids/VLPs. In particular, previous theoretical and experimental studies have identified both nucleation and growth pathways and "en masse" assembly pathways, where a large number of proteins first associate with the cargo before structural reorganisation into the final capsid architecture. The relative importance of these mechanisms depends on the balance between protein-protein interactions, protein-nucleic-acid interactions, and cargo properties. We attempted to keep the focus of the current work on the assembly of empty VLPs, naturally, the next step is to combine assembly of empty particles with the assembly around nucleic acid to reveal how the template properties affect the molecular mechanism of assembly.

Regarding the claims in the conclusion section, we believe that this stems from the chosen language rather than that there is 'no experimental limitation'. We have therefore revised the relevant text to clarify that our claim concerns the broad applicability of the approach beyond virus assembly, rather than the absence of experimental challenges in more complex biological systems.

We have changed the corresponding sentence as follows:

"Beyond the implications for our understanding of the fundamental principles driving and controlling self-assembly, our approach represents a novel framework for studying biomolecular assembly and dynamics more broadly. The approach of characterising pairwise interactions, quantifying equilibrium distributions and monitoring individual assembly events and dynamics is general: Here, it was applied to a simple, homo-oligomeric, analyte, but ***owing to the universality of mass measurements our approach can be applied to a broad range of multi-molecular systems,***

opening up new avenues for studying biomolecular dynamics and mechanisms in the context of biological function and regulation."
